# Global Distribution, Ecotoxicity, and Treatment Technologies of Emerging Contaminants in Aquatic Environments: A Recent Five-Year Review

**DOI:** 10.3390/toxics13080616

**Published:** 2025-07-24

**Authors:** Yue Li, Yihui Li, Siyuan Zhang, Tianyi Gao, Zhaoyi Gao, Chin Wei Lai, Ping Xiang, Fengqi Yang

**Affiliations:** 1 Nanotechnology & Catalysis Research Centre (NANOCAT), Institute for Advanced Studies (IAS), University of Malaya (UM), Kuala Lumpur 50603, Malaysia; liyue@hhtc.edu.cn (Y.L.); 23104481@siswa.um.edu.my (S.Z.); 23069422@siswa.um.edu.my (T.G.); 2 Institute of Art and Design, Huaihua University, Huaihua 418000, China; liyihui@hhtc.edu.cn; 3 Faculty of Business and Management, City University Malaysia, Petaling Jaya 46100, Malaysia; gaozhaoyi874@gmail.com; 4 Institute of Environmental Remediation and Human Health, School of Ecology and Environment, Southwest Forestry University, Kunming 650224, China; xiangping@swfu.edu.cn

**Keywords:** ECs, aquatic environments, global distribution, ecotoxicity, removal method

## Abstract

With the rapid progression of global industrialization and urbanization, emerging contaminants (ECs) have become pervasive in environmental media, posing considerable risks to ecosystems and human health. While multidisciplinary evidence continues to accumulate regarding their environmental persistence and bioaccumulative hazards, critical knowledge gaps persist in understanding their spatiotemporal distribution, cross-media migration mechanisms, and cascading ecotoxicological consequences. This review systematically investigates the global distribution patterns of ECs in aquatic environments over the past five years and evaluates their potential ecological risks. Furthermore, it examines the performance of various treatment technologies, focusing on economic cost, efficiency, and environmental sustainability. Methodologically aligned with PRISMA 2020 guidelines, this study implements dual independent screening protocols, stringent inclusion–exclusion criteria (n = 327 studies). Key findings reveal the following: (1) Occurrences of ECs show geographical clustering in highly industrialized river basins, particularly in Asia (37.05%), Europe (24.31%), and North America (14.01%), where agricultural pharmaceuticals and fluorinated compounds contribute disproportionately to environmental loading. (2) Complex transboundary pollutant transport through atmospheric deposition and oceanic currents, coupled with compound-specific partitioning behaviors across water–sediment–air interfaces. (3) Emerging hybrid treatment systems (e.g., catalytic membrane bioreactors, plasma-assisted advanced oxidation) achieve > 90% removal for recalcitrant ECs, though requiring 15–40% cost reductions for scalable implementation. This work provides actionable insights for developing adaptive regulatory frameworks and advancing green chemistry principles in environmental engineering practice.

## 1. Introduction

In recent decades, the rapid expansion of global industrialization has driven an exponential surge in the production of synthetic chemicals, with petrochemical products (25.7%), specialty chemicals (26.2%), and polymer materials (19.2%) dominating the market [1]. These anthropogenic chemicals are persistently released into the environment through diverse pathways. Their sources include pharmaceuticals, agriculture, food processing, and electronics manufacturing, leading to the emergence of a novel category of ECs [2]. Unlike conventional pollutants, ECs represent a complex and diverse class of substances identified in recent years due to advancements in analytical techniques [3]. These ECs commonly encompass personal care products (PCPs), endocrine-disrupting chemicals (EDCs), antibiotics, MPs (MPs), per- and polyfluoroalkyl substances (PFASs), and disinfection by-products [4]. Although present in low concentrations, ECs exhibit pronounced bioaccumulation, persistence, and toxicity [5]. Their chemical diversity and variability render their migration and transformation processes highly intricate, thereby complicating ecological risk assessment and remediation strategies [6]. Furthermore, these contaminants pose significant threats to both ecosystems and human health, being linked to neurological disorders, endocrine disruption, cardiovascular diseases, and reproductive dysfunction, thus presenting considerable challenges to environmental safety and public well-being [7,8].

ECs enter the environment through multiple pathways. Direct emissions from healthcare facilities often result in elevated pollutant concentrations, with 58% of pharmaceutical residues resistant to removal by conventional treatment methods [9]. Agricultural non-point source pollution is particularly pronounced during heavy rainfall, with surface runoff concentrations of ECs reaching 0.11–25 ppm [10]. Atmospheric deposition contributes to over 30 types of ECs, with marked concentration increases observed during the rainy season [11]. Advances in detection technologies have significantly enhanced the identification of ECs, improving accuracy, sensitivity, and specificity [12]. Fang et al. [13] validated a dual-enzyme electrode sensor capable of detecting methyl salicylate with a detection limit of 2.3 μM. Similarly, optical sensors have achieved notable advancements in real-time monitoring, as demonstrated by Chen et al. [14], who developed a carbon dot-based trichannel optical detection method for PFOS with a detection limit of 10.8 ppb. Moreover, integrating multiple sensing technologies has further improved detection efficacy. Li et al. [15] engineered a molecularly imprinted Fe-doped porous carbon composite sensor with a detection limit of 0.2 nM for lomefloxacin. These innovations underscore the transformative role of novel sensing technologies in enhancing environmental contaminant detection’s precision, selectivity, and responsiveness.

The presence and adverse effects of ECs in ecosystems present significant threats to ecological integrity and human health [5,16]. Vera-Chang et al. [17] demonstrated that the antidepressant fluoxetine reduces reproductive success and induces embryonic deformities in zebrafish. Morales et al. [18] found that EDCs, such as BPA, disrupt freshwater snails’ growth, reproduction, and survival by mimicking natural hormones like estrogen, thereby interfering with normal hormonal processes. Additionally, the cumulative ecological risks of ECs have been substantiated, as illustrated by Li et al. [19], who examined the persistence and bioaccumulation potential of perfluorooctane sulfonate. MPs have been detected in various human tissues, including the lungs, bloodstream, and placenta [20]. Research indicates that MPs induce oxidative stress, disrupt metabolic processes, impair gut microbiota and gastrointestinal function, and adversely affect the liver, cardiopulmonary, and immune systems, ultimately diminishing reproductive health [21]. Niu et al. [22] highlighted the role of extensive antibiotic use in propagating antibiotic resistance genes (ARGs) in the environment, potentially facilitating the emergence of superbugs. Moreover, the interaction between MPs and antibiotics exacerbates the persistence of antibiotics in the environment, compounding the challenge of antibiotic resistance. Addressing ECs through practical measures and technologies is thus critical to ensuring environmental safety [23].

To date, the occurrence and migration of ECs in aquatic environments have been extensively investigated. Nevertheless, most studies remain confined to specific pollutant types or geographically restricted areas, lacking a comprehensive and systematic assessment of the global distribution patterns and migration mechanisms of diverse EC categories. To bridge this knowledge gap, the present review aims to (1) systematically examine the sources, spatial distributions, and migratory pathways of ECs (PCPs, EDCs, antibiotics, MPs, and PFAS) across global aquatic systems; (2) elucidate their potential ecological and human health risks; and (3) evaluate the feasibility and efficacy of various remediation technologies—including physical, chemical, biological, and integrated approaches—under varying environmental conditions. Ultimately, this review endeavors to offer practical insights for the optimization of management strategies and to promote a more scientific, systematic, and sustainable framework for EC governance.

## 2. Literature Review Methodology

### 2.1. Search Strategy

**Figure 1:**
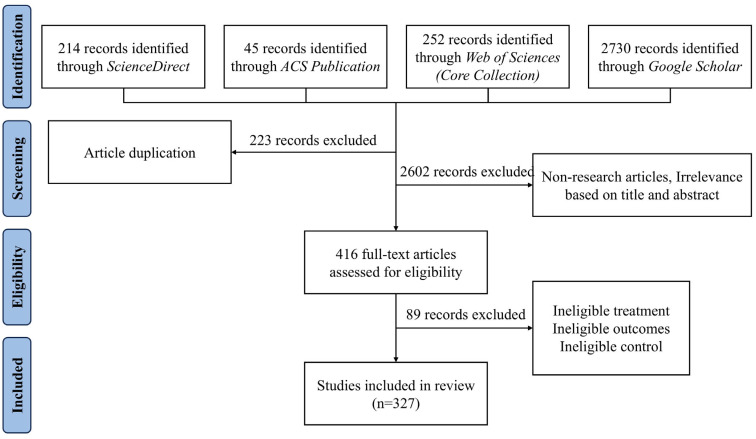
PRISMA flow diagram of the study selection process.

This systematic review complies with the Preferred Reporting Items for Systematic Reviews and Meta-Analyses (PRISMA) guidelines (Figure 1). To identify relevant studies, a comprehensive search was performed across ACS Publications, ScienceDirect, Google Scholar, and Web of Science databases. The search period spanned from January 2020 to December 2024, focusing on recent advances concerning five categories of ECs: PCPs, antibiotics, EDCs, PFAS, and MPs in wastewater, surface water, and groundwater. The rationale for limiting the search to a 5-year period was to provide a comprehensive yet current snapshot of ECs specifically for evaluating recent spatial distribution trends. This 5-year criterion was applied exclusively to the distribution aspects of this review. Foundational and older references beyond the 5-year scope were selectively incorporated in the Introduction and Discussion sections to provide historical context, explain technological evolutions, and establish comparative trends. The detailed search strategy and results are provided in Table S1.

### 2.2. Inclusion and Exclusion Criteria

The studies included in this review met the following criteria: (1) published in English; (2) focused on the five specified categories of ECs; (3) limited to research articles (excluding reviews, book chapters, theses, conference papers, and editorial letters); (4) provided concentration data and sampling information for target compounds in aquatic environments (wastewater, surface water, groundwater); (5) explicitly described analytical methods and quality assurance/quality control (QA/QC) procedures or allowed their inference; (6) reported concentration data including mean values, median values, or concentration ranges to enable comparative analysis.

It is important to note that the geographical distribution of reported ECs is shaped by disparities in regional monitoring efforts and research intensity. The absence of EC reports in certain regions should not be interpreted as a lack of contamination but rather as a reflection of limited environmental surveillance.

Database searches initially yielded 3241 records. After removing 223 duplicates, 3018 records were screened based on title, abstract, and article type, excluding 2602 non-qualifying records. The remaining 416 records underwent full-text screening, where 89 were excluded due to inadequate treatment, insufficient results, or inappropriate controls. Ultimately 327 studies were included in the systematic review (Table S2).

### 2.3. Data Extraction

Two researchers independently extracted and cross-validated the following information from the selected studies: (1) Publication Details: Authors, publication year, and study region (country or specific area); (2) Target Pollutants: Specific categories of ECs (PCPs, antibiotics, EDCs, PFAS, MPs), along with maximum, minimum, and mean/median concentrations (ng/L or items/L), and occurrence frequencies across continents; (3) Sampling and Analytical Methods: Details on water body type (wastewater, surface water, groundwater), sampling dates, geographical coordinates, detection methods, and limits of detection/quantification (LOD/LOQ). For graphical data, numerical values were extracted using GetData Graph Digitizer 2.26, with concentrations below LOD or LOQ recorded as half the respective limits. Measurements were excluded if LOD or LOQ were excessively high; (4) Water Treatment Processes and Removal Efficiencies: Information on treatment processes, including specific wastewater treatment plant units (biological treatment, membrane filtration, disinfection, etc.) and reduction rates for target pollutants. (5) Handling of Overlapping Categories: For chemicals with overlapping classifications, each was categorized based on its primary characteristics and main role within the study. When a compound could fit multiple categories, it was assigned according to its dominant function, with overlaps noted for clarity.

## 3. Distribution of EC Occurrences in Published Studies

**Figure 2:**
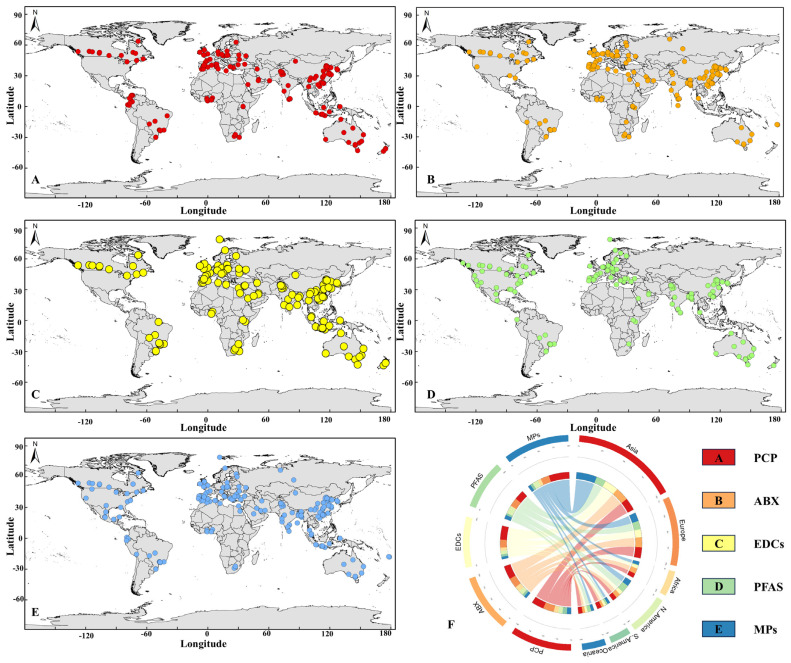
Geographical distribution of literature reports on selected ECs in aquatic environments (based on study occurrence, not concentrations): (**A**) PCPs, (**B**) antibiotics (ABX), (**C**) EDCs, (**D**) PFASs, (**E**) MPs, (**F**) Global distribution of publications on 5 types of ECs.

Advancements in environmental monitoring and analytical technologies have facilitated the detection and reporting of numerous ECs that were previously undetected in natural ecosystems [24]. The spatial distribution of literature reporting on ECs—including PCPs, antibiotics, EDCs, PFAS, and MPs—exhibits considerable heterogeneity (Figure 2F). Asia represents the most frequently reported region, contributing 37.05% of global research outputs on ECs over the past five years, with particular emphasis on MPs and antibiotics. This pattern may reflect the region’s high population density, extensive use of agricultural antibiotics, and ongoing challenges in plastic waste management [25,26]. Europe follows with 24.31% of the reports, where PCPs and EDCs are commonly studied, potentially due to historical industrial activities and high pharmaceutical consumption [27,28]. North America accounts for 14.01% of the documented studies, with PFASs frequently examined, likely linked to the region’s legacy in chemical manufacturing [29]. In contrast, Africa and South America represent 8.92% and 7.32% of reported studies, respectively. Antibiotics are the primary focus in Africa, while MPs dominate in South America, potentially reflecting regional concerns related to agricultural practices, fisheries, and plastic pollution [30,31]. Oceania contributes 8.39% of the global EC-related literature, with MPs and PFASs being the most reported contaminants. This trend may be influenced by the region’s geographic characteristics and exposure to international shipping activities [32,33].

### 3.1. Spatial Distribution of PCPs

PCPs comprise various substances for human application, such as preservatives, disinfectants, insect repellents, fragrances, and ultraviolet (UV) protectants. Their primary functions are cleaning and safeguarding personal hygiene and aesthetic appearance [34]. As illustrated in Figure 2A, PCP contamination is pervasive worldwide. North America and Europe exhibit relatively high occurrences of PCP pollution, likely due to advanced pharmaceutical industries, well-established healthcare systems, and significant PCP consumption [34,35]. In Asia, high detection frequencies are also observed, driven by pressures from large populations, increasing consumption demand, and underdeveloped wastewater treatment and environmental regulatory infrastructure [36]. Monitoring sites in developing regions like South America and Africa remain sparse. Nevertheless, PCPs have been detected in some Latin American countries and South Africa, reflecting a growing recognition of PCP-related environmental risks and underscoring the necessity for a more robust global research network and regulatory framework.

### 3.2. Spatial Distribution of Antibiotics

Antibiotics are compounds with the capacity to inhibit or destroy microorganisms [37]. Figure 2B depicts the global distribution of antibiotics across various regions. Several Asian countries exhibit relatively high incidences of antibiotic contamination, indicative of focused attention and extensive research in these areas. Factors such as high population density, well-developed healthcare infrastructure, and significant antibiotic consumption contribute to this trend [38]. Moreover, intensive livestock farming and aquaculture activities amplify antibiotic discharges into the environment [39,40]. North America and Europe also exhibit high antibiotic distribution frequencies, attributable to the increasing scale of antibiotic use in both medical and agricultural applications [41]. Research and monitoring efforts in South America and Africa are comparatively limited, highlighting resource constraints and insufficient monitoring capacities. Additionally, inadequate regulatory frameworks and enforcement mechanisms in these regions exacerbate antibiotic use and discharge, posing a significant pollution challenge.

### 3.3. Spatial Distribution of EDCs

EDCs include natural hormones, such as estrone (E1), 17β-estradiol (E2), and estriol (E3), as well as synthetic hormones like 17α-ethinylestradiol (EE2) [42]. The global distribution of EDCs is substantial, with notable research attention focused on regions of high industrialization and intensive agricultural activity (Figure 2C). Developed regions in North America and Europe feature comprehensive monitoring systems and high-density distribution records, reflecting the long-term accumulation of chemical production and consumption [43].

In rapidly developing areas of East and South Asia, significant quantities of potential EDCs are discharged through agricultural practices and plastic production, resulting in a complex, multi-center, and multi-pathway pollution pattern [44]. Despite the relatively long history of EDC research, significant gaps remain in both investigation and regulation in underdeveloped regions such as Africa, underscoring the critical need for improved monitoring and governance [45,46].

### 3.4. Spatial Distribution of PFASs

PFASs are synthetic organic fluorinated compounds known for their exceptional chemical stability, resulting in environmental persistence and bioaccumulation [47]. Figure 2D indicates that PFASs have been detected extensively in aquatic environments worldwide, ranging from surface waters in industrial areas (0.11–26.5 ng/L) to snow layers in the Arctic (50 pg/L) [48,49]. Sampling sites in North America and Europe are densely distributed, primarily in regions characterized by high industrialization and urbanization. This prevalence is attributed to the widespread use of PFASs in industrial and consumer applications, including surface protectants, flame retardants, and firefighting foams [47]. In East Asia, particularly along coastal industrial zones, research on PFASs is significant. These areas face increasingly severe PFAS accumulation due to concentrated chemical industries, manufacturing activities, and large populations [50,51]. Conversely, PFAS detections in South America and Africa remain relatively sparse. These regions often lack adequate regulatory frameworks, advanced detection methodologies, and research investments, likely underestimating the true extent of PFAS pollution. These underexplored regions urgently require comprehensive and systematic monitoring studies [52,53].

### 3.5. Spatial Distribution of MPs

MPs, recognized as prominent emerging environmental pollutants, have drawn substantial attention owing to their pervasive distribution and potential ecological risks [54]. MPs are extensively distributed globally (Figure 2E). Research efforts are more pronounced in regions with dense populations, advanced economies, and established industrial systems, such as North America, Europe, and East Asia’s coastal areas. The continuous increase in global plastic production exacerbates MP pollution, with estimates projecting approximately 12 billion tons of plastic waste in landfills or natural environments by 2050. This makes MP contamination one of the most critical environmental challenges of the modern era [55]. During the COVID-19 pandemic, the surge in disposable plastic usage, including an estimated 129 billion masks and 65 billion gloves per month globally, significantly intensified MP pollution [56].

Overall, the distribution patterns of ECs are influenced by a confluence of factors, including population density, levels of economic development, industrial activity, and international trade [57,58,59]. Asia and Europe are major pollution hotspots, driven by industrial processes and consumer demand, while North America’s high contamination levels are largely attributed to historical chemical production. Africa, South America, and Oceania necessitate increased attention to cross-regional pollutant migration and the cumulative effects of local pollution.

## 4. Sources and Migration of ECs

**Figure 3:**
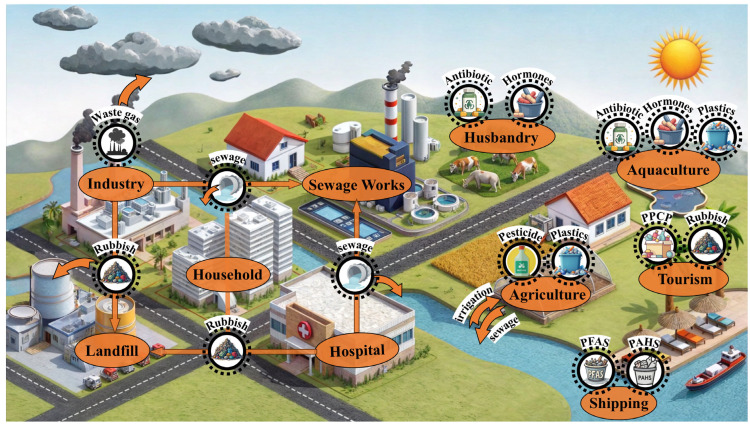
Schematic diagram of the multifaceted sources of EC production, utilization, and environmental release.

**Figure 4:**
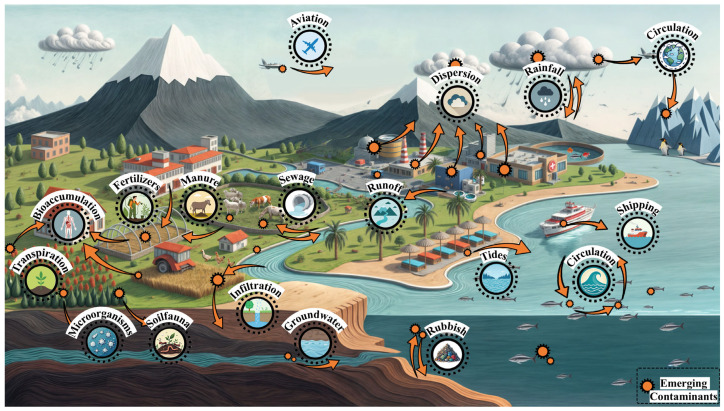
Pathways of ECs entering the environment and its migration.

ECs enter the environment through various pathways, including industrial processes, agricultural activities, healthcare operations, wastewater discharge, and landfill sites [24] (Figure 3). Moreover, their migration and transformation in the environment display substantial multidimensional complexity (Figure 4). Global environmental monitoring data reveal that ECs interact across different media and undergo cross-regional transmission via atmospheric circulation, hydrological cycles, and biological chains, resulting in persistent ecological impacts [7,60,61].

### 4.1. Industrial Activities

The emission of ECs from industrial activities arises from the production characteristics and insufficient treatment technologies in various sectors [62,63]. The pharmaceutical industry reports average concentrations of halogenated hydrocarbons from point sources reaching 18.9 ppm, constituting 28.8% of its total volatile organic compounds (VOCs) emissions. These VOCs directly impair air quality and contribute to the formation of secondary pollutants, such as ozone, through photochemical reactions, thereby aggravating regional air pollution [64]. The electronics manufacturing industry presents significant environmental and health risks, primarily associated with heavy metal contamination, the release of organic pollutants, and the generation of secondary pollutants [65]. These findings underscore the high concentration, significant hazard, and treatment challenges associated with ECs from various industries, highlighting the urgent necessity for technological innovation and policy intervention to enable effective governance.

### 4.2. Agricultural Activities

ECs in agricultural activities predominantly originate from agricultural chemicals, waste, and irrigation water, presenting distinct and far-reaching environmental and health risks. First, these pollutants are derived from various sources, including excessive use of pesticides, insecticides, fertilizers, and soil amendments. These substances not only contaminate soil and water through runoff or leaching but also disrupt ecosystem stability due to their persistence [66]. Lastly, agricultural activities significantly contribute to greenhouse gas emissions, particularly methane and nitrous oxide. These gases exacerbate global warming and alter pollutant migration pathways, thereby heightening ecological and environmental risks [67,68,69].

### 4.3. Emissions from Healthcare Activities

The extensive use and discharge of pharmaceuticals constitute the primary sources of ECs in healthcare. Studies indicate that compounds such as amoxicillin and caffeine are frequently detected in aquatic environments due to widespread usage, with concentrations reaching micrograms per liter or higher, posing considerable threats to aquatic ecosystems [70]. Improper disposal of medical waste is another critical source of pollution. Since the SARS outbreak 2002, medical waste generation has surged by approximately 240%, with projections forecasting a further 50% increase by 2030. This includes mercury-containing medical devices, where mercury emissions in some regions exceed those from domestic wastewater by more than double, leading to long-term soil and water contamination [71]. Hospital wastewater also contains pharmaceutical residues and hazardous substances such as bacteria and radioactive isotopes, which are often discharged into sewer systems without adequate treatment. This practice poses severe environmental and public health risks, as hospital wastewater typically exhibits higher concentrations of pathogens and chemicals compared to municipal wastewater [72].

### 4.4. Wastewater Treatment Plants and Landfills

Wastewater treatment plants (WWTPs) and landfills represent major sources of ECs, harboring substantial quantities of complex and persistent contaminants [73]. These pollutants infiltrate groundwater and soil through leakage, adversely impacting the environment and human health. ECs in wastewater and waste exhibit considerable long-term contamination potential [74]. Huang et al. [75] discovered that persistent pollutants, including pharmaceuticals and personal care products, exert prolonged effects on surface waters well beyond their initial introduction. PFAS concentrations in landfill leachate reached 12.7 μg/L; even landfills closed for over 30 years exhibited high pollutant levels [76]. Over time, the risks associated with groundwater pollution from wastewater and landfill leachate progressively increase [77].

Current research on EC sources and their fate encounters two critical challenges necessitating breakthrough solutions. First, existing research paradigms are constrained, favoring fragmented analyses of individual pollutants, media, or local environments. This limitation hinders the establishment of an integrated research framework incorporating multi-dimensional, multi-scale, and multi-omics approaches. Second, mechanistic understanding and model construction remain inadequate. There is a lack of comprehensive knowledge regarding EC migration, transformation, and bioaccumulation mechanisms across trans-regional, trans-media, and multi-biogeochemical cycles. This deficiency is exacerbated by the absence of high-precision dynamic models and long-term monitoring data.

### 4.5. Co-Occurrence of ECs

ECs are frequently detected concurrently within aquatic environments due to their overlapping sources [78]. PFAS concentrations ranged from several hundred to several thousand ng/L, while antibiotics were present at ng/L levels, and MPs ranged from hundreds to over a thousand particles per liter [79]. Furthermore, the co-existence of PCPs, antibiotics, EDCs, PFAS, and MPs has been consistently reported in urban surface waters and effluents from wastewater treatment plants, highlighting that ECs rarely exist as isolated pollutants in real-world settings, but rather as complex and interactive mixtures.

The coexistence of multiple ECs can significantly influence their individual environmental behaviors (Table S3). Owing to their high specific surface area and hydrophobicity, MPs readily act as carriers for hydrophobic organic pollutants. These substances can adsorb onto MP surfaces, thereby facilitating their mobility and enhancing their dispersion and transport distances in aquatic environments [80]. Recent research has highlighted the strong affinity between MPs and PFASs, noting that PFAS molecules can adhere to MP surfaces, consequently increasing their bioavailability to aquatic organisms [81]. Although studies investigating such sorption/desorption interactions are still relatively limited, growing evidence suggests that these processes play a critical role in shaping the distribution, fate, and transport of coexisting ECs.

## 5. Risks of ECs to Ecosystems and Human Health

### 5.1. Aquatic Ecosystem Risks

**Figure 5:**
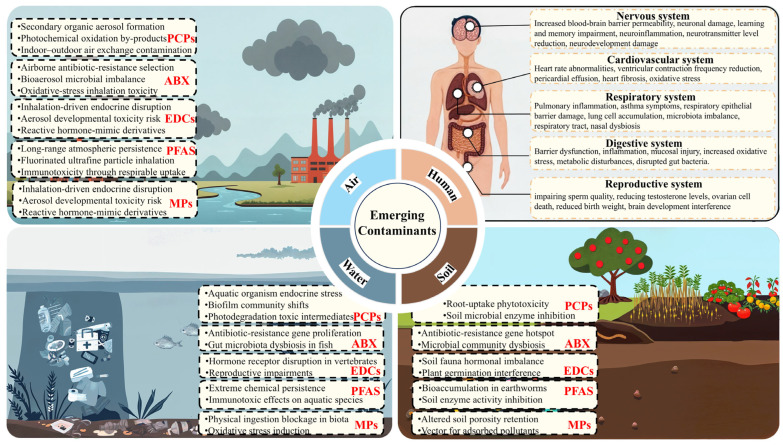
Environmental and health risks of ECs.

ECs pose multifaceted threats to aquatic ecosystems through persistence, diffusion, and bioaccumulation mechanisms, leading to complex ecological challenges [82] (Figure 5). Antibiotics, due to the inefficiencies of conventional wastewater treatment, substantially promote the dissemination of ARGs. The selective proliferation of resistant bacteria further diminishes microbial diversity and destabilizes ecosystem functions [83]. EDCs interfere with hormonal regulation, significantly impacting fish sex differentiation and reproductive capacity. Their accumulation in aquatic environments disrupts lower trophic organisms and amplifies ecological toxicity through bioaccumulation in higher trophic levels [83,84]. MPs exacerbate the toxicity of antibiotics and PFASs due to their high adsorption capacity, while directly impairing metabolism and reproduction in aquatic organisms. At elevated concentrations, algae growth inhibition rates can reach 22.8% [85,86]. PFAS, characterized by chemical stability and long-chain molecular structures, exhibit wastewater treatment removal rates below 48% [87]. These compounds bioaccumulate significantly in aquatic species, with bioconcentration factors in mussels recorded at approximately 120 L/kg for PFDA and 48–81 L/kg for PFOS, leading to prolonged endocrine and immune system dysfunction [88]. Nanomaterials compromise cellular membranes and disrupt redox reactions in plankton, causing metabolic disorders in pathways such as amino acid metabolism, energy metabolism, and substance transport. At high concentrations (50 mg/L), growth inhibition rates can reach 20–30%, accompanied by significant alterations in the expression of protein synthesis-related genes [86]. Collectively, ECs jeopardize the functional balance and biodiversity of aquatic ecosystems through bioaccumulation, ecological toxicity, and metabolic disruption [89].

### 5.2. Atmospheric Environmental Risks

ECs exert escalating impacts on atmospheric systems and associated ecological processes through intricate physical and chemical pathways [7]. Antibiotics transmitted via aerosols intensify the spread of ARGs. Research indicates elevated concentrations of ARGs in aerosols, with their transmission potentially extending thousands of kilometers under favorable wind and humidity conditions, significantly exacerbating regional resistance proliferation [22]. EDCs enter human systems through ingestion, inhalation, and dermal absorption of indoor dust. BPA, widely detected in indoor dust (0.01–32 μg/g), disrupts endocrine functions even at low exposure levels [90]. MPs, capable of long-range atmospheric transport, act as vectors for antibiotics and other pollutants, compounding the toxicity and complexity of atmospheric contamination [91]. PFASs are transported across long distances in both gaseous and particulate phases. Their environmental behavior is influenced by chain length, with long-chain PFASs (C > 6) exhibiting a strong tendency to bind with particles, while ultrashort-chain perfluoroalkyl acids (C2–C4) are more likely to transfer from the atmosphere to surface waters. Persistent atmospheric deposition makes PFASs a critical environmental issue [92]. Due to their ultrafine size, nanomaterials penetrate biological systems via inhalation, inducing cytotoxicity and DNA damage in alveolar epithelial cells. They disrupt lysosomal membrane integrity and increase permeability, potentially leading to inflammation and cell death [93].

### 5.3. Soil Ecosystem Risks

ECs pose significant threats to the health and functionality of soil ecosystems through complex migration, transformation, and accumulation processes, impacting microbial communities, ecological balance, and food chain safety [94]. Research indicates that average antibiotic concentrations in northern China’s soils reach 21.79 µg/kg, with levels in farmlands and orchards being 2–3 times higher than in other land-use types. This contamination, strongly associated with applying organic fertilizers and wastewater irrigation, reduces soil microbial diversity and heightens the risk of ARG dissemination [95]. EDCs represent another pollutant class with profound effects on soil ecosystems. These substances demonstrate strong biotoxicity, potentially disrupting soil microbial metabolic pathways and enzyme activities, thereby diminishing soil ecological functionality [96]. MP particles, characterized by high mobility and persistence in soils, create complex pollution systems in conjunction with other contaminants. Through adsorption and biofilm formation, they impair soil organism functions, inducing reproductive toxicity and DNA damage [97]. Due to their nanoscale size and high surface area, nanomaterials present considerable toxicity risks to soil ecosystems. They alter microbial metabolic pathways, disrupt root-zone environments, and traverse food chains to higher trophic levels, exerting long-term adverse effects on ecosystems and human health [98].

### 5.4. Human Health Risks

ECs infiltrate the human body through various pathways, posing significant health risks. Due to their persistence and bioaccumulation, they cause intricate disruptions to metabolic, immune, endocrine, and gene expression systems [99]. Antibiotics enter the human body via contaminated water sources and the food chain, accelerating the spread of ARGs [100]. Studies reveal that environmental exposure to antibiotics significantly upregulates resistance gene expression in gut microbiota, diminishing the efficacy of antibacterial drugs and heightening the risk of infections [101]. EDCs interfere with hormonal functions by mimicking or blocking endogenous hormone signals, potentially causing health conditions such as obesity, diabetes, reproductive disorders, and cancers [102]. As ubiquitous pollutants, MPs are ingested through inhalation, food, and drinking water, posing health threats, including oxidative stress, inflammatory responses, and metabolic dysregulation [20]. Exposure to PFASs has been associated with a range of adverse health effects, including developmental and reproductive toxicity, immunotoxicity, neurotoxicity, hepatotoxicity, genotoxicity, endocrine disruption, and carcinogenicity [103]. Furthermore, mixed chemical exposures may exhibit additive, antagonistic, or synergistic effects, complicating health risk assessments [104].

Current research on the health risks of ECs in water, air, soil ecosystems, and humans exhibits several critical deficiencies. First, research paradigms remain predominantly focused on individual pollutants’ environmental behavior and toxicity, neglecting the systematic investigation of interactions and synergistic toxicities among multiple pollutants. Second, the limited scope of studies across diverse ecological conditions impedes the development of globally applicable and context-specific risk assessments. Moreover, insufficient attention to nutrient cycling, carbon fixation, and alterations in the structure and functionality of key biological communities in aquatic, atmospheric, and soil ecosystems constrains the understanding of overall ecosystem health and service capacity. In terms of human health, causal research on chronic exposure to ECs remains inadequate. Additionally, uneven regional data distribution, particularly in developing countries, hampers the availability of comprehensive risk assessments and decision-support information. This disparity further restricts the scientific basis for formulating and effectively implementing strategies to manage ECs.

## 6. Removal Methods for ECs

Wastewater treatment technologies can be broadly categorized into four main types based on their mechanisms and processes: physical, chemical, biological, and hybrid [105]. Each method offers distinct advantages and applicability, playing a crucial role in mitigating ECs (Tables S4 and S5).

### 6.1. Physical Removal Methods

Physical remediation techniques exhibit notable advantages and diverse application potential in the treatment of ECs [106]. Studies indicate that during the primary treatment phase of MPs, removal rates for fibrous and granular pollutants range from 79% to 83%, with efficiencies for particles and fragments reaching as high as 91% [107]. However, Monsalvo et al. [108] demonstrated that anaerobic membrane bioreactor processes exhibit limited efficacy for certain persistent pesticides, such as atrazine and linuron, achieving removal rates of only 6.8% and 10.5%, respectively. Similarly, Singer et al. [109] reported that conventional secondary treatment and tertiary sand filtration yielded removal rates below 50% for pesticides like atrazine and diuron, and even negative removal rates for certain compounds such as metolachlor. These pronounced discrepancies underscore the critical influence of the physicochemical properties of pollutants on the effectiveness of treatment processes [110].

#### Adsorption Treatment

Adsorption technology is extensively employed in the removal of ECs [111]. The adsorption efficiency and selectivity are influenced by the physicochemical properties of the pollutants and the functional characteristics of the adsorbent surface [112]. Natural materials are frequently used as adsorbents in water treatment due to their abundance and low cost [113]. Clay minerals, with their high specific surface areas and layered structures, are among the most effective natural adsorbents, demonstrating remarkable adsorption performance for pharmaceutical pollutants [114]. Thiebault et al. [115] demonstrated that sodium montmorillonite achieved adsorption capacities of 223.5 mg/g for tramadol and 263.4 mg/g for dothiepin. Similarly, Huang et al. [116] found that modified β-cyclodextrin materials exhibited excellent adsorption performance and reusability for BPA. Agricultural waste, a cost-effective adsorbent, is increasingly utilized in wastewater treatment [117].

### 6.2. Chemical Removal Methods

Chemical precipitation, coagulation–flocculation, and other chemical processes play pivotal roles in addressing conventional pollutants in water treatment. Advanced oxidation processes (AOPs) have emerged as indispensable technical solutions for the efficient degradation and mineralization of pollutants, effectively addressing new challenges in water pollution. AOPs encompass catalytic ozonation, electrochemical oxidation, photocatalytic oxidation, and ultrasonic oxidation, all exhibiting exceptional removal efficiencies for ECs [118]. Catalytic ozonation generates hydroxyl radicals through the synergistic interaction of ozone and catalysts, facilitating the rapid decomposition of pollutants [119]. Zhang et al. [120] demonstrated that degradation efficiency using a Cu-CuFe_2_O_4_ catalyst is 2.2 times higher than that achieved by ozonation alone. Electrochemical oxidation enables the degradation of recalcitrant pollutants without adding additional chemical reagents. Photocatalytic oxidation employs UV light to activate semiconductor catalysts, generating hydroxyl radicals and achieving significant degradation of various ECs [121]. Ultrasonic oxidation, regarded as an environmentally friendly AOP technology, has garnered attention for its high efficiency in wastewater treatment [122]. Zhang et al. [123] observed that with an ozone flow rate of 80 L/h and an ultrasonic power density of 80 W/L, cyanide removal reached 99.96%, compared to 92% with ozone alone.

### 6.3. Biological Remediation Methods

Aerobic and anaerobic technologies form the foundation of biological removal methods, offering distinct advantages and considerable application potential in wastewater treatment [124]. Activated sludge systems are particularly effective for removing antibiotic pharmaceuticals. Watkinson et al. [125] demonstrated that activated sludge treatment reduced ciprofloxacin, trimethoprim, and lincomycin concentrations from 0.34–4.6 μg/L to 0.05–0.6 μg/L. In anaerobic treatment, Akcal Comoglu et al. [126] reported that upflow anaerobic sludge blanket reactors achieved chemical COD removal efficiencies of 93–97%. Notably, combined anaerobic–aerobic processes often yield superior outcomes; Shi et al. [127] confirmed that such hybrid systems achieved COD removal rates of 94.7% and 91.8%, respectively.

Enzymatic treatment technology, characterized by high efficiency, environmental sustainability, and operational simplicity, shows significant promise for EC remediation [128]. Studies revealed that chloroperoxidase at a concentration of 20 μg/mL degraded over 88% of levofloxacin and rifaximin within 30 min [129]. Furthermore, Das et al. [130] demonstrated that laccase immobilized on magnetic nanoparticles removed over 99% of chlorpyrifos within 12 h under pH 7 and 60 °C conditions. Both peroxidase and laccase effectively treat industrial pollutants, including phenolic compounds [131].

### 6.4. Hybrid Treatment Methods

Biologically activated carbon (BAC) forms biofilms on the surface of activated carbon, enabling biological regeneration of the adsorbent and extending its lifespan, thereby effectively removing ECs. Studies revealed that BAC achieved removal rates of over 85% for 52 organic micropollutants in WWTP effluent. For specific drugs such as ibuprofen, acetaminophen, and simvastatin, the removal efficiency reached 96% [132]. Additionally, BAC systems exhibit stable removal performance for EDCs, with BPA removal rates of 90–95% [133]. Furthermore, studies showed that BAC filtration achieved an 81% removal rate for MPs during drinking water treatment [134].

Constructed wetland (CW) technology, as a nature-friendly water treatment method, has shown significant potential in removing ECs. Horizontal subsurface flow wetlands achieved removal rates of 39–98% for complex organic pollutants, with more than 50% removal efficiency for 12 pollutants, highlighting their potential in low-cost wastewater treatment [135]. Vertical flow wetlands, combining plant root filtration and activated carbon matrix adsorption, achieved over 90% overall removal rates for 27 pollutants, with 18 pollutants meeting national discharge standards, further optimizing system efficiency and stability [136]. Studies further indicated that CW performance is influenced by key parameters such as hydraulic retention time, dissolved oxygen, and oxidation–reduction potential, with vertical flow systems demonstrating optimal performance due to superior oxygen transfer properties [137]. Through system optimization and integrated mechanisms, CW technology offers high efficiency and sustainability and provides viable solutions for low-cost and resource-limited regions.

### 6.5. Interference of Treatment Processes Under Multi-ECs Conditions

The simultaneous presence of multiple ECs frequently leads to competitive interactions during treatment processes. In adsorption-based technologies, contaminants exhibiting higher hydrophobicity, elevated concentrations, or favorable structural attributes often preferentially occupy adsorption sites, thereby diminishing the removal efficiency of coexisting pollutants. Consequently, the actual elimination performance for individual ECs in mixed systems is typically inferior to that observed under single-contaminant scenarios [138]. For instance, studies on ozonation have revealed that varying fractions of organic matter can significantly influence the degradation kinetics of target micropollutants, often suppressing their overall removal efficiency [139]. Such competitive interference underscores the need for process refinement or the incorporation of synergistic treatment approaches, which may, however, increase operational complexity, elevate treatment costs, and lead to the formation of unintended byproducts.

MPs play dual and complex roles in multi-contaminant systems, acting both as pollutant carriers and shielding agents. Their large specific surface area and hydrophobic nature enable them to adsorb a broad spectrum of ECs, thereby reducing the freely dissolved concentrations of these contaminants in the aqueous phase. In the short term, this adsorption may appear beneficial by mitigating interference with biological or oxidative degradation pathways. However, MPs are typically resistant to removal by conventional treatment methods and may be discharged into receiving environments along with the pollutants they carry [140]. This phenomenon becomes particularly concerning when considering the potential for synergistic ecotoxicological effects associated with MP-bound contaminant complexes. Empirical studies have demonstrated that ingestion of such complexes by aquatic organisms can prolong the residence time of toxic substances within biological systems, thereby amplifying their toxic effects [141]. Taken together, these findings highlight the critical role of MPs as “hidden carriers” in multi-contaminant scenarios. Their ability to simultaneously serve as pollutant vectors and protective microenvironments for other ECs presents significant obstacles to effective remediation and poses serious implications for environmental and public health protection.

## 7. Conclusions and Prospection

With the accelerated global industrialization and urbanization, the environmental distribution and potential hazards of ECs have garnered increasing attention. Compared to previous research on emerging contaminants, studies from the past five years have exhibited explosive growth. Research on ECs is undergoing an unparalleled metamorphosis, marked by perpetually shifting distribution patterns, a deepening comprehension of ecotoxicological mechanisms, and rapidly advancing remediation technologies. A meta-analysis of 327 publications (2020–2024) delineates a dynamic landscape wherein global contamination profiles evolve alongside industrial transitions and exogenous shocks—for example, the 240% escalation in medical waste during the COVID-19 pandemic that profoundly reconfigured worldwide microplastic dispersal. Conceptual frameworks have progressed from isolated compound evaluations to intricate assessments of multi-contaminant consortia, underscoring the predominance of synergistic toxicities and the role of microplastics as clandestine vectors amplifying co-occurring pollutants. Concomitantly, remedial innovations have proliferated: hybrid treatment systems now routinely achieve > 90% removal of recalcitrant species, although economic constraints continue to impede large-scale deployment. Future research should strengthen the following aspects:

(1) Shift from the single-pollutant and single-medium studies paradigm to comprehensive research on multi-pollutant interactions and all environmental media. Emphasis should be placed on exploring ECs’ migration and transformation mechanisms across water, soil, and air media, as well as the interactions, synergistic, or antagonistic toxic effects among multiple pollutants. This will help construct multidimensional environmental behavior models, providing a scientific basis for comprehensive environmental risk assessment.
(2) Strengthen research on ECs in developing countries. Enhancing monitoring capabilities can address data gaps in developing regions, contributing to a globally unified database of ECs. This would improve the accuracy and comprehensiveness of global risk assessments.
(3) Develop technologies for the collaborative treatment of multiple pollutants, enabling the efficient removal of complex pollution systems. Additionally, optimize the integration of treatment technologies to enhance overall remediation performance. Focus should also be given to the economic feasibility of treatment technologies, reducing implementation costs, particularly in resource-constrained areas.
(4) Enhance public awareness and education regarding ECs. This will increase societal support for pollution control measures. Furthermore, establish multi-stakeholder collaboration platforms to integrate efforts from governments, research institutions, enterprises, and the public, thereby advancing the implementation of emerging pollutant management and providing a robust social foundation for achieving environmental sustainability.

## Data Availability

Data is contained within the article or Supplementary Materials.

## Supplementary Materials

The following supporting information can be downloaded at: https://www.mdpi.com/article/10.3390/toxics13080616/s1, Table S1: The detailed search strategy and results for each database (From 2020 to December 2024); Table S2: Country wise concentrations (ng/L, particles/L) of selected ECs in different water matrices; Table S3: Interaction effects of representative ECs mixtures on aquatic organisms and microbial communities; Table S4: The operational cost of different treatment technologies utilized for EC removal from wastewater matrix; Table S5: Removal efficiency of ECs in different water treatment technology. References [142,143,144,145,146,147,148,149,150,151,152,153,154,155,156,157,158,159,160,161,162,163,164,165,166,167,168,169,170,171,172,173,174,175,176,177,178,179,180,181,182,183,184,185,186,187,188,189,190,191,192,193,194,195,196,197,198,199,200,201,202,203,204,205,206,207,208,209,210,211,212,213,214,215,216,217,218,219,220,221,222,223,224,225,226,227,228,229,230,231,232,233,234,235,236,237,238,239,240,241,242,243,244,245,246,247,248,249,250,251,252,253,254,255,256,257,258,259,260,261,262,263,264,265,266,267,268,269,270,271,272,273,274,275,276,277,278,279,280,281,282,283,284,285,286,287,288,289,290,291,292,293,294,295,296,297,298,299,300,301,302,303,304,305,306,307,308,309,310,311,312,313,314,315,316,317,318,319,320,321,322,323,324,325,326,327,328,329,330,331,332,333,334,335,336,337,338,339,340,341,342,343,344,345,346,347,348,349,350,351,352,353,354,355,356,357,358,359,360,361,362,363,364,365,366,367,368,369,370,371,372,373,374,375,376,377,378,379,380,381,382,383,384,385,386,387,388,389,390,391,392,393,394,395,396,397,398,399,400,401,402,403,404,405,406,407,408,409,410,411,412,413,414,415,416,417,418,419,420,421,422,423,424,425,426,427,428,429,430,431,432,433,434,435,436,437,438,439,440,441,442,443,444,445,446,447,448,449,450,451,452,453,454,455,456,457,458,459,460,461,462,463,464,465,466,467,468,469,470,471,472,473,474,475,476,477,478,479,480,481,482,483,484,485,486,487,488,489,490,491,492,493,494,495,496,497,498,499,500,501,502,503,504,505,506,507,508,509,510,511,512,513,514,515,516,517,518,519,520,521,522,523,524,525,526,527,528,529,530,531,532,533,534,535,536,537,538,539,540,541,542,543] are cited in the supplementary materials.
