# Supplementary material for: Global Distribution, Ecotoxicity, and Treatment Technologies of Emerging Contaminants in Aquatic Environments: A Recent Five-Year Review
Source: Toxics. 2025 Jul 24;13(8):616. doi: 10.3390/toxics13080616 (PMC12389987; doi:10.3390/toxics13080616)
Supplement: Supplementary file 1 [file toxics-13-00616-s001.zip › toxics-3696114-supplementary.pdf]

Supplementary material

Table captions

Table S1. The detailed search strategy and results for each database (From 2020 to December 2024).

Table S2. Country wise concentrations (ng/L, particles/L) of selected ECs in different water matrices.

Table S3. Interaction effects of representative ECs mixtures on aquatic organisms and microbial communities

Table S4. The operational cost of different treatment technologies utilized for EC removal from wastewater matrix.

Table S5. Removal efficiency of ECs in different water treatment technology.

Table S1. The detailed search strategy and results for each database (From 2020 to December 2024).

**Databases**	**Search strategy**	**Records**
Science Direct	#1 TITLE (("emerging contaminants" OR "emerging pollutants" OR "emerging chemicals" OR "pharmaceuticals and personal care products" OR "PPCPs" OR "Ibuprofen" OR "Triclosan" OR "Sulfamethoxazole" OR "Erythromycin" OR "Tetracycline" OR "Ciprofloxacin" OR "Bisphenol A" OR "17β-Estradiol" OR "microplastics" OR "PFAS" OR "PFOA" OR "PFOS"))	3689
	#2 TITLE (("drinking water" OR "surface water" OR "groundwater" OR "wastewater" OR "marine water" OR "freshwater contamination" OR "river water contamination" OR "lake water pollution" OR "water quality assessment" OR "estuarine water" OR "coastal water" OR "estuaries" OR "river mouth" OR "water pollution" OR "aquatic ecosystems"))	7445
	#3 TITLE(("Algeria" OR "Argentina" OR "Australia" OR "Austria" OR "Bangladesh" OR "Brazil" OR "Canada" OR "China" OR "Colombia" OR "Cyprus" OR "Czech Republic" OR "Ecuador" OR "Egypt" OR "Fiji" OR "Finland" OR "France" OR "Germany" OR "Ghana" OR "Greenland" OR "Iceland" OR "India" OR "Italy" OR "Kenya" OR "Malaysia" OR "Mexico" OR "New Zealand" OR "Nigeria" OR "Norway" OR "Pakistan" OR "Peru" OR "Poland" OR "Portugal" OR "Russia" OR "Saudi Arabia" OR "Slovenia" OR "South Africa" OR "Spain" OR "Sri Lanka" OR "Tanzania" OR "Tunisia" OR "Turkey" OR "UAE" OR "United Kingdom" OR "Ukraine" OR "United States" OR "Zambia"))	1214488
	#4 #1 AND #2 AND #3	**214**
ACS Publication	#1 TITLE-ABS-KEY(("emerging contaminants" OR "emerging pollutants" OR "emerging chemicals" OR "pharmaceuticals and personal care products" OR "PPCPs" OR "Ibuprofen" OR "Triclosan" OR "Sulfamethoxazole" OR "Erythromycin" OR "Tetracycline" OR "Ciprofloxacin" OR "Bisphenol A" OR "17β-Estradiol" OR "microplastics" OR "PFAS" OR "PFOA" OR "PFOS"))	73
	#2 TITLE-ABS- KEY (("drinking water" OR "surface water" OR "groundwater" OR "wastewater" OR "marine water" OR "freshwater contamination" OR "river water contamination" OR "lake water pollution" OR "water quality assessment" OR "estuarine water" OR "coastal water" OR "estuaries" OR "river mouth" OR "water pollution" OR "aquatic ecosystems"))	134
	#3 TITLE-ABS-KEY(("Algeria" OR "Argentina" OR "Australia" OR "Austria" OR "Bangladesh" OR "Brazil" OR "Canada" OR "China" OR "Colombia" OR "Cyprus" OR "Czech Republic" OR "Ecuador" OR "Egypt" OR "Fiji" OR "Finland" OR "France" OR "Germany" OR "Ghana" OR "Greenland" OR "Iceland" OR "India" OR "Italy" OR "Kenya" OR "Malaysia" OR "Mexico" OR "New Zealand" OR "Nigeria" OR "Norway" OR "Pakistan" OR "Peru" OR "Poland" OR "Portugal" OR "Russia" OR "Saudi Arabia" OR "Slovenia" OR "South Africa" OR "Spain" OR "Sri Lanka" OR "Tanzania" OR "Tunisia" OR "Turkey" OR "UAE" OR "United Kingdom" OR "Ukraine" OR "United States" OR "Zambia"))	685
	#4 #1 AND #2 AND #3	**45**
Web of	# 1 TI = ("emerging contaminants" OR "emerging pollutants" OR "emerging	84643

Sciences Core Collection	chemicals" OR "pharmaceuticals and personal care products" OR "PPCPs" OR "Ibuprofen" OR "Triclosan" OR "Sulfamethoxazole" OR "Erythromycin" OR "Tetracycline" OR "Ciprofloxacin" OR "Bisphenol A" OR "17β-Estradiol" OR "microplastics" OR "PFAS" OR "PFOA" OR "PFOS")	
	#2 TI = ("drinking water" OR "surface water" OR "groundwater" OR "wastewater" OR "marine water" OR "freshwater contamination" OR "river water contamination" OR "lake water pollution" OR "water quality assessment" OR "estuarine water" OR "coastal water" OR "estuaries" OR "river mouth" OR "water pollution" OR "aquatic ecosystems")	199469
	#3 TI = ("Algeria" OR "Argentina" OR "Australia" OR "Austria" OR "Bangladesh" OR "Brazil" OR "Canada" OR "China" OR "Colombia" OR "Cyprus" OR "Czech Republic" OR "Ecuador" OR "Egypt" OR "Fiji" OR "Finland" OR "France" OR "Germany" OR "Ghana" OR "Greenland" OR "Iceland" OR "India" OR "Italy" OR "Kenya" OR "Malaysia" OR "Mexico" OR "New Zealand" OR "Nigeria" OR "Norway" OR "Pakistan" OR "Peru" OR "Poland" OR "Portugal" OR "Russia" OR "Saudi Arabia" OR "Slovenia" OR "South Africa" OR "Spain" OR "Sri Lanka" OR "Tanzania" OR "Tunisia" OR "Turkey" OR "UAE" OR "United Kingdom" OR "Ukraine" OR "United States" OR "Zambia")	2745706
	#4 #1 AND #2 AND #3	**252**
Google Scholar	#1 all in title: "emerging contaminants" OR "emerging pollutants" OR "emerging chemicals" OR "pharmaceuticals and personal care products" OR "PPCPs" OR "Ibuprofen" OR "Triclosan" OR "Sulfamethoxazole" OR "Erythromycin" OR "Tetracycline" OR "Ciprofloxacin" OR "Bisphenol A" OR "17β-Estradiol" OR "microplastics" OR "PFAS" OR "PFOA" OR "PFOS"	154000
	#2 all in title: "drinking water" OR "surface water" OR "groundwater" OR "wastewater" OR "marine water" OR "freshwater contamination" OR "river water contamination" OR "lake water pollution" OR "water quality assessment" OR "estuarine water" OR "coastal water" OR "estuaries" OR "river mouth" OR "water pollution" OR "aquatic ecosystems"	153000
	#3 all in title: "Algeria" OR "Argentina" OR "Australia" OR "Austria" OR "Bangladesh" OR "Brazil" OR "Canada" OR "China" OR "Colombia" OR "Cyprus" OR "Czech Republic" OR "Ecuador" OR "Egypt" OR "Fiji" OR "Finland" OR "France" OR "Germany" OR "Ghana" OR "Greenland" OR "Iceland" OR "India" OR "Italy" OR "Kenya" OR "Malaysia" OR "Mexico" OR "New Zealand" OR "Nigeria" OR "Norway" OR "Pakistan" OR "Peru" OR "Poland" OR "Portugal" OR "Russia" OR "Saudi Arabia" OR "Slovenia" OR "South Africa" OR "Spain" OR "Sri Lanka" OR "Tanzania" OR "Tunisia" OR "Turkey" OR "UAE" OR "United Kingdom" OR "Ukraine" OR "United States" OR "Zambia"	1450000
	#4 #1 AND #2 AND #3	**2730**

Table S2. Country wise concentrations (ng/L, particles/L) of selected ECs in different water matrices

Country	Matrix	TCS	IBU	SMX	ERY	TC	NOR	BPA	E2	PFAS	MPs	Ref.
**Algeria**	SW										6.17–71.7	(Bentaallah et al., 2024; Grini et al., 2024; Kheireddine et al., 2024; Setiti et al., 2021; Tata et al., 2020)
**Australia**	TWW		17-191					480-32000		0.22-504		(Ahmed et al., 2023; Fan et al., 2023; Li et al., 2022b; Nguyen et al., 2024; Szabo et al., 2023; Tang et al., 2020; Ziajahromi et al., 2021)
	SW	<0.1–3.1	150	14						<LOD-526	<LOD-1990	(Anim et al., 2020; Leterme et al., 2023; McKenzie et al., 2020; Nan et al., 2020; Paige et al., 2024; Rauert et al., 2022)
	GW		80	78							16-973	(Currell et al., 2024; McKenzie et al., 2020; Nguyen et al., 2022; Samandra et al., 2022a)
	DW										0-80	(Samandra et al., 2022b)
**Austria**	TWW									140–213		(Müller et al., 2023)
	SW									1.2–2.5		(Müller et al., 2023)

	GW											
**Bangladesh**	SW			<LOD-428	<LOD–120	<LOD–190	<LOD–710			1.8–19.0	0.57–2660	(Al Nahian et al., 2022; Al Nahian et al., 2023; Alam et al., 2023; Angeles et al., 2020; Fardullah et al., 2024; Haque et al., 2023; Hossain et al., 2022; Islam et al., 2024; Islam et al., 2022; Islam et al., 2023; Jahan et al., 2024; Mercy et al., 2023; Morales-McDevitt et al., 2022; Nawar et al., 2023; Odora et al., 2024; Parvin et al., 2022; Riya et al., 2024; Salma et al., 2025; Samrat Hossain et al., 2024)
	GW										4-75	(Paray et al., 2024)
	DW										14–56	(Hossain et al., 2023)
**Bulgaria**	SW										0.00062	(Berov and Klayn, 2020)
	DW							11080				(Moid AlAmmari et al., 2020)
**Brazil**	TWW			1374								(de Oliveira Santos et al., 2022)

	SW	2-789	3-759060	0.6-572	0-0.19	0-0.12	0- 292	2.8-2880	0.3-6808	2-50	0.06-330.2	(Arsand et al., 2020; Castro et al., 2020; Chaves et al., 2020; Chaves et al., 2021; de Moraes et al., 2024; de Rezende and Mounteer, 2023; Ferraz et al., 2020; Madeira et al., 2023; Reichert et al., 2020; Sabino et al., 2021; Santos et al., 2020; Santos et al., 2022; Trindade et al., 2023)
	DW							4.0–135	<200–575.9		105.8	(Ferraz et al., 2020; Oliveira et al., 2024; Teixeira et al., 2021)
	GW		<LOQ-458700									(do Nascimento et al., 2023)
**Canada**	TWW		44-15200	34.7-2010	18-21			0-1030	22-86	0.072–1060	0.8-15.4	(Gewurtz et al., 2024; Grbić et al., 2020; Prajapati et al., 2021; Schwartz et al., 2021; Stroski et al., 2020; Vaudreuil et al., 2022; Zhou et al., 2022)
	SW		44-367	2-87	23	15		3.05–1888.51	1.8	8.3-42.1		(Bujaczek et al., 2021; Kleywegt et al., 2020; Lalonde and Garron, 2020; Schwartz et al., 2021; Vaudreuil et al., 2022)
	DW		0.08-0.19	2					1.8	<0.001 - 108		(Kleywegt et al., 2020; Munoz et al., 2023)

**China**	TWW	5.0–11.3 ng/L		<LOQ-250.6	0.5-4.4	<LOQ-1.1	<LOQ-133.1	25.2-41.6	<LOD -108	7.35-342		(Guo et al., 2022; Jiang et al., 2020; Lei et al., 2020; Li et al., 2020; Liu et al., 2023; Lopez-Herguedas et al., 2023; Luo et al., 2023; Qiao et al., 2024; Wang et al., 2021d; Xu et al., 2023; Yang et al., 2022; Zhang et al., 2024; Zheng et al., 2020; Zhong et al., 2022)
	SW	0.002-394	30.9-341.6	0-44.11	0-56.2	2.7-6.4	6.144–382.3	4.12-321	0-12.4		46.7–204	(An et al., 2022; Chen et al., 2020b; Fan et al., 2021a; Fan et al., 2021b; Fan et al., 2022; Huang et al., 2021; Jin et al., 2022; Li et al., 2021; Liang et al., 2022; Lin et al., 2020; Liu et al., 2021; Mo et al., 2024; Pei et al., 2022; Wang et al., 2021c; Wang et al., 2022c; Wu et al., 2020; Xu et al., 2022; Yang et al., 2021; Yang et al., 2020a; Yang et al., 2020b; Yi et al., 2023; Yu et al., 2024; Yu et al., 2023; Yuan et al., 2020; Zhang et al., 2020a; Zhang et al., 2020b)
	GW			1.41-20060			0.44–45.40			282000	87–6832	(Liu et al., 2022; Mu et al., 2022; Qiao et al., 2023; Qin et al., 2020; Shi et al., 2022; Shu et al., 2023; Wei et al., 2024; Wu et al., 2022)

	DW	3.72–7.16		<LOQ-17.0	0.013-3462	<LOQ-96	<LOQ-27	<LOQ–324		<LOQ to 13.4 ng/L	2–23	(Ben et al., 2020; Liu et al., 2020; Wang et al., 2021a; Wang et al., 2021b; Wang et al., 2022b; Zhou et al., 2021)
**Colombia**	TWW		620-1050	50-208760				274.29-1623.94				(Gallego-Ríos and Peñuela, 2021; Hernández-Fernández et al., 2022; Hernández et al., 2024; Rodríguez-Rodríguez et al., 2023; Serna-Galvis et al., 2022)
	SW	100-790	<LOD - 218000								3-1387	(Arregocés-Garcés et al., 2024; Cerón-Vivas and Peñuela Mesa, 2024; Elles-Pérez et al., 2024; Garcés-Ordóñez et al., 2022; Hernández et al., 2024; Pemberthy M et al., 2020; Porras-Rojas et al., 2023; Rojas-Luna et al., 2023; Romero-Murillo et al., 2023; Rusinque-Quintero et al., 2022)
**Czech Republic**	TWW	17	460-500	530	16							(Diaz-Sosa et al., 2020)
	SW		505-12900	33	1360–2600			14-800		2.97		(Datel and Hrabankova, 2020; Dvorakova et al., 2023; Hrkal et al., 2023; Šauer et al., 2021)
	GW										2.5-20	(Brožová et al., 2023)

	DW		≤52		≤28					0.48-90.8	20-180	(Datel and Hrabankova, 2020; Dvorakova et al., 2023; Halfar et al., 2023; Halfar et al., 2024; Jurikova et al., 2022; Kozisek et al., 2025)
**Ecuador**	SW			<LOD						0.07-2.37	3-490	(Capparelli et al., 2021; Cipriani-Avila et al., 2023; Fiedler et al., 2024; López et al., 2023)
	DW			<LOD								(Jara-Negrete et al., 2023)
**Egypt**	SW			41.56–5.95				85500			0.46 ± 0.28	(Abdel Ghani et al., 2022; Emam et al., 2023; Radwan et al., 2020; Sayed et al., 2021)
**Fiji**	SW			13	6.3		23				0.8-2	(Dehm et al., 2021; Vanukon et al., 2025)
**Finland**	TWW	1500-2500	2400-89000	<LOQ-71	5-682	3.9-43			0.52-1.8	3-16000	0-0.15	(Äystö et al., 2023; Kortesmäki et al., 2020; Reinikainen et al., 2022; Uurasjärvi et al., 2020)
	GW									2500		(Reinikainen et al., 2022)
**France**	TWW	10-100		≤5–200	≤100	≤10	≤300					(Aemig et al., 2021; Haenni et al., 2022; Lefebvre et al., 2023)
	SW	5-50	50-200	≤100	≤50	≤5	≤50	≤300	≤5	≤10	0.00015	(Aemig et al., 2021; Azzi et al., 2021; de Carvalho et al., 2021)

	DW							10870-41190				(Moid AlAmmari et al., 2020)
**Germany**	TWW	16000	22000	22.9-34.9	126.2-290.4	<LOD-15.4	43.8-230.6	10–650		<10–650		(Nickel et al., 2021; Rodriguez-Mozaz et al., 2020; Tappert et al., 2024)
	SW	<10000	<LOQ–186	<LOQ–32	<LOQ–26			<LOQ–81	<LOQ–1	<LOQ–7110		(Adomat and Grischek, 2024; Göckener et al., 2023; Nickel et al., 2021; Tappert et al., 2024)
	GW											
	DW							10440		1.5–11.2		(Hron et al., 2024; Moid AlAmmari et al., 2020)
**Ghana**	TWW		19540									(Kodom et al., 2021)
	SW		10.0–319.0	145-840							1.58–4.16	(Blankson et al., 2022)
**Greece**	TWW	0-1.2								<15	2.29	(Ofrydopoulou et al., 2022; Zeri et al., 2021)
	SW	<25								0.3-23.1	8.1-27.7	(Anagnostopoulpou et al., 2022; Ofrydopoulou et al., 2022; Zeri et al., 2021)

	GW										30-35	(Perraki et al., 2024)
**India**	TWW	15000	43510	52000				3100	200			(Saxena et al., 2021; Thalla and Vannarath, 2020)
	SW							40–4460	0.007–4.5		13-760	(Chakraborty et al., 2021; Lechthaler et al., 2021; Mukhopadhyay et al., 2020; Napper et al., 2021)
	GW			0–67						1.2–32.4	2–80	(K et al., 2021; Selvam et al., 2021; Sharma et al., 2024)
	DW										382 ± 205	(Yadav et al., 2022)
**Indonesia**	TWW	470 ± 64	2300					378				(Astuti et al., 2023)
	SW	<LOD-293						<LOD–1070	<LOD–31.2		44.7 -431	(Falahudin et al., 2020; Ismanto et al., 2022; Lestari et al., 2020; Sulistyowati et al., 2022; Suteja et al., 2021; Wicaksono et al., 2021; Yuan et al., 2023)
**Iran**	TWW										0.423-70.66	(Alavian Petroody et al., 2020; Hajiouni et al., 2022; Naji et al., 2021)
	SW	1600±2100									86-362	(Hosseini et al., 2020; Moazeni et al., 2023)

	DW	400-1400						980000				(Moazeni et al., 2023; Parto et al., 2022)
**Ireland**	TWW	7		44					3.5			(Rapp-Wright et al., 2023a)
	GW									1.6–96		(Harrad et al., 2020)
**Italy**	TWW									200-950		(Cantoni et al., 2024)
	SW										0.0009–0.013	(Campanale et al., 2020)
**Kenya**	TWW			7800-20600	<LOD	0-1200			0.61-270	10–470	0.004–0.51	(Chepchirchir et al., 2024; Ngigi et al., 2019; Park et al., 2020; Tanui et al., 2025)
	SW			670-6840	<LOD-1900							(Kandie et al., 2020; Ngigi et al., 2019)
	GW		2–240	<LOQ-258.2		2–154						(K'Oreje et al., 2022; Karimi et al., 2023)
**Korea**	TWW		5988	295.6					14.8	4.5-12	0.6–2.3	(Lee et al., 2020)
**Kuwait**	TWW			212-852			153-865				1-12	(Gevao et al., 2022; Uddin et al., 2022)

**Lebanon**	SW				121-268							(Azzi et al., 2021)
	DW							390-12410				(Moid AlAmmari et al., 2020)
**Malaysia**	SW							0-37000	0-15.5		0.7-3.4	(Moid AlAmmari et al., 2020; Nazifa et al., 2020; Shehab et al., 2020)
	DW										8-22	(Praveena et al., 2022)
**Mexico**	TWW	700						200000–1000000	0.4–15.1	23.5-176.9		(López-Velázquez et al., 2021; Lugo-Bueno et al., 2022; Rodríguez-Varela et al., 2021)
	SW										12–381	(Cooney et al., 2023; Sánchez-Campos et al., 2024)
	DW										510-7100	(Morales-Arredondo et al., 2023)
	GW							5000–25000				(Cruz-López et al., 2020)
**Netherlands**	SW			1–150						0.4-4900		(Gebbink and van Leeuwen, 2020; Sabri et al., 2020; Sadia et al., 2023; Zwart et al., 2020)

	GW									<5–25000		(Gebbink and van Leeuwen, 2020)
	DW									0.1-25.5		(Gebbink and van Leeuwen, 2020; Sadia et al., 2023)
**New Zealand**	TWW	<0.6–429						<0.6–429		0.1–13		(Emnet et al., 2020)
	SW	<0.2–9.4								0.2-0.5		(Emnet et al., 2020)
	GW	1.94–2.03	5.3–175					1.28–423	0.95–5.15			(Close et al., 2021)
**Nigeria**	SW	64	<4-2740	<1-3180	<1-275	<LOD-30.1		31–868			0.20–1.69	(Adeyinka et al., 2022; Ebele et al., 2020; Oni and Sanni, 2022; Ugboka et al., 2022)
	GW		<4-2250	<1-64	<1	<LOD-30.1						(Ebele et al., 2020)
	DW		<4-50	<1-7	<1			17–920				(Ebele et al., 2020; Ugboka et al., 2022)
**Norway**	SW							0.5-1.4		0.1-2.4	47-73	(Ademollo et al., 2021; Herzke et al., 2021)
	DW									0.071–2.7 ng/L		(Grung et al., 2024)

**Pakistan**	TWW	22.6–45.8		318–16009			–2558	324–492	12.46–31.6			(Ashfaq et al., 2023; Zafar et al., 2021)
	SW	7.49–19.3	<LOD-–416	<LOD-76				42.5–71.2	0-5.04	19.9–722	0.025–88	(Ashfaq et al., 2023; Dilshad et al., 2022; Nousheen et al., 2022; Riaz et al., 2023; Schilling Costello et al., 2023)
	GW		<LOD–1961	0.04–29.1		<LOD-83.1	<LOD-13.3					(Khan et al., 2022; Zainab et al., 2021)
**Peru**	TWW			2360-5180	810-1210		2980-4230					(Nieto-Juárez et al., 2021)
	SW			4360							0.004-0.17	(Fabregat-Safont et al., 2023; Larrea Valdivia et al., 2025; Nieto-Juárez et al., 2021)
**Poland**	TWW	220-7000	359-393000	987	<LOD-9.71			>16000			1.6-7	(Ormaniec, 2024; Sekudewicz et al., 2021; Ślósarczyk and Witkowski, 2024; Styszko et al., 2021)
	SW	500-1600	170-7652	103.5-77800	1.57				7.97			(Ślósarczyk and Witkowski, 2024; Styszko et al., 2021)
	GW								2.6–18.7			(Rusiniak et al., 2021)
**Portugal**	TWW		<LOD-7200	34–1300	86-320							(Rodrigues et al., 2021; Silva et al., 2021)

	SW		93–160	43–310	0.14-2819		339-120			82.4-104	0-0.34	(Fernandes et al., 2020; Sá et al., 2022; Silva et al., 2021; Sousa et al., 2020)
**Russia**	TWW											(Chernova et al., 2021)
	SW					0.1-66.2					0.04-11	(Chernova et al., 2021; Frank et al., 2022; Frank et al., 2021)
**Saudi Arabia**	TWW			0.12–37.15	<LOD–4.02		<LOD–541.79					(Semerjian et al., 2023)
	SW	<LOD –33.5	<LOD–2407	0.16–3.18	<LOD–0.94		17.11–109.74	<LOD–484.9		<LOQ–956		(Ali et al., 2021; Mostafa et al., 2023; Picó et al., 2020; Picó et al., 2021; Semerjian et al., 2023)
	DW							290–41190			0.99–26	(Almaiman et al., 2021; Picó et al., 2020)
**Slovenia**	TWW							4.7–34,921	6.3–69.4			(Grobin et al., 2024)
	SW							0.12–278	0.16–1.4		0.44-60	(Valentić et al., 2022)
	GW							17–6551	<LOD–0.25			(Durcik et al., 2023)

**South Africa**	TWW	1732 - 6980	2504	20-9890	0-100	0 -100	0-80		1.08–53.8			(Bakare and Adeyinka, 2022; Mhlongo et al., 2023; Mhuka et al., 2020; Oharisi et al., 2023)
	SW	<LOQ	<LOQ–0.03	<LOQ–53828	<LOQ–2700				0.25–14.8		0.1 ± 0.6	(Oke, 2024; Saad et al., 2024; Verlicchi and Grillini, 2020)
	DW							<LOQ–181		3920-4950		(Onipe et al., 2021; Verlicchi and Grillini, 2020)
**Spain**	TWW	890–26990	1-1100	<1-33000	2-78				0-0.4		16.40± 7.9	(Alexa et al., 2022; Castaño-Trias et al., 2024; Franco et al., 2021; Llamas-Dios et al., 2021; Lopez et al., 2022; Martínez-Alcalá et al., 2021; Sol et al., 2022; Solaun et al., 2022)
	SW	0-0.2	<1-160	<1-3180	11–130	11	940			28-180		(Castaño-Trias et al., 2024; Fonseca et al., 2020; Lopez et al., 2022; Montes et al., 2023; Royano et al., 2023; Sol et al., 2022)
	GW	435–3130										(Llamas-Dios et al., 2021; Lopez et al., 2022)
**Sri Lanka**	TWW											
	SW		1-84	1-934							2–36	(Bandara et al., 2023; Quyen et al., 2021; Shafi et al., 2023)

	GW		15	8.4						3		(Chandrajith et al., 2025)
Sweden	TWW									50–1124		(Fredriksson et al., 2022; Gobelius et al., 2023)
	SW									<LOD-15		(Sörengård et al., 2022)
	GW									1–1000		(Mussabek et al., 2023; Sörengård et al., 2022)
	DW									1-8.2		(Mussabek et al., 2023; Säve-Söderbergh et al., 2024)
Tunisia	TWW			494.5		3.33-60	43-484.67					(Nasri et al., 2024)
	GW		43–7384	0.4–9.32	11.3–54.2			5–8				(Khezami et al., 2024)
Turkey	TWW	342–565	38–735								637.5–2274	(Erdem et al., 2024; Üstün-Odabaşı et al., 2020; Üstün et al., 2022)
	SW									394–24754	4.42–55.67	(Akdemir and Gedik, 2023; Akdogan et al., 2023; Aydin et al., 2024; Terzi et al., 2022)
	DW							290-11660			3.2 ± 4.0	(Kılıç et al., 2024; Moid AlAmmari et al., 2020; Sönmez et al., 2023)

**UAE**	TWW			14.68	1.28		244.25					(Semerjian et al., 2023)
	SW			1.53	0.44		60.02					(Semerjian et al., 2023)
**UK**	TWW			30.2-123.4	48-361.8	<LOD-231.2	6.7-69.4				37.7-129.13	(Cunsolo et al., 2021; Harley-Nyang et al., 2022; Rodriguez-Mozaz et al., 2020)
	SW	76	3.3-298	6	0.02–12.80				4.21–17.47		0.26–0.68	(Ebele et al., 2020; Higgins and Turner, 2023; Rapp-Wright et al., 2023b; Vane et al., 2022)
	GW		32	<1								(Ebele et al., 2020)
	DW		12	<1							6-100	(Al-Mansoori et al., 2025)
**Ukraine**	SW	3100		<400	<400	<700		526	80		0-0.289	(Ho et al., 2020; Nikolopoulou et al., 2022; Snigirova et al., 2024)
	GW							100				(Nikolopoulou et al., 2022)
**USA**	TWW			<5	<2	39.8	94-102			<LOD-677		(Bai and Son, 2021; Chen et al., 2023; da Silva et al., 2022; Sinkway et al., 2024)

	SW									2300-97000	180.1–315.3	(Bai and Son, 2021; Hain et al., 2023; Haque et al., 2024; Pétré et al., 2021; Ridall et al., 2023; Schwichtenberg et al., 2020; Stovall and Bratton, 2022; Xiong et al., 2022)
	GW									20–4773		(Chen et al., 2023; McMahon et al., 2022; Pétré et al., 2021; Silver et al., 2023)
	DW									1.0–100		(Andrews and Naidenko, 2020; Babayev et al., 2022; Duru et al., 2024; Johnson, 2022; Liddie et al., 2024; Von Behren et al., 2024)
**Zambia**	TWW			80–30040								(Ngumba et al., 2020)
	SW			<LOQ–11,800								(Ngumba et al., 2020)
	GW			<LOQ–880								(Ngumba et al., 2020)

TWW: Treated wastewater, SW: Surface water, DW: Drinking Water, GW: Ground water, LOD: Limit of Detection, LOQ: Limit of Quantification, TCS：Triclosan，IBU：Ibuprofen，SMX：Sulfamethoxazole，ERY：Erythromycin，TC：Tetracycline，NOR：Norfloxacin，BPA：Bisphenol A，E2：Estradiol，PFAS：Per- and Polyfluoroalkyl Substances，MPs：Microplastics

Table S3. Interaction effects of representative ECs mixtures on aquatic organisms and microbial communities

ECs combination	Test organism	Effect	Concentration	Interaction type	Ref
**TCS + PE-MPs**	Marine diatom	Growth-inhibition rate	TCS: 0–200 μg L^{-1} ;	Antagonistic: adsorption of TCS to MPs lowered algal toxicity	(Khan et al., 2022; Zhu et al., 2019)
**TCS+ aPE-MPs**	African clawed frog; tadpoles	SOD, CAT	TCS: 2 μg L^{-1}; aPE-MPs: 1 mg L^{-1}	Synergistic: co-exposure intensified oxidative stress and neuro-toxicity	(Lin et al., 2024)
**TCS+ MPs**	Fresh-water cyanobacterium Anabaena sp.	Decline in growth rate and chlorophyll-a	TCS adsorbed: 18–105 μg g^{-1}	Synergistic: MPs acted as carriers, markedly suppressing algal growth	(Verdú et al., 2021)
**SMX+ TMP**	Mixed marine microalgal assemblage	Cell-density growth inhibition	SMX: 1 μg L^{-1}; TMP: 0.2 μg L^{-1}	Synergistic: mixture reduced algal growth far more than single antibiotics	(Sharma et al., 2021)
**SMX+ ERY**	Green alga Raphidocelis subcapitata	96 h growth-inhibition rate; antioxidant-enzyme variation	SMX: 0.5–8 mg L^{-1}; ERY: 0.05–0.8 mg L^{-1} (10:1)	Additive/Synergistic: SOD	(Zhang et al., 2021)
**NOR+ TYL**	Green alga Raphidocelis subcapitata	96 h growth rate	NOR: 0.1–1 mg L^{-1}; TYL: 0.1–1 mg L^{-1}	Antagonistic: combined algal inhibition lower than predicted	(Yang et al., 2008)
**BPA+ PS-MPs**	Zebrafish larvae (Danio rerio)	96 h cumulative mortality; swimming behaviour	BPA: 10 μg L^{-1}; PS-MPs: 0.1–10 mg L^{-1}	Synergistic: co-exposure raised mortality 6.7–30 % and sharply decreased activity	(Jian et al., 2024)

**PFOS+ PS-MPs**	Adult female zebrafish	Hepatic oxidative-stress gut-microbiota diversity	PFOS: 10 µg L^{-1}; PS-MPs: 50 µg L^{-1}	Synergistic: co-exposure aggravated liver immunotoxicity and dysbiosis vs. single-exposure	(Jian et al., 2024)
**PFOS + PFOA**	Zebrafish embryos	96 h lethality	PFOS: 0.2–5 mg L^{-1}; PFOA mixed at various ratios	Ratio-dependent: synergy at PFOS:PFOA = 1: 6 or 1:1, antagonism at 1:3	(Ding et al., 2013)
**TCS+ multiple antibiotics**	Model bacteria	Inhibition-zone diameter	TCS: 0.2 mg L^{-1}; antibiotics at MIC gradients	Synergy / antagonism: synergistic toward *Bacillus* spp., antagonistic toward *Pseudomonas aeruginosa*	(Shrestha et al., 2020)
**IBU + TCS + SMX + CBZ[3]**	Anaerobic-digestion sludge microbiota	Cell viability; methane & CO_2 production	Each pollutant ≈ 100 µg L^{-1}	Synergy / additive: mixture caused higher cell death and digestion inhibition than single compounds (except TCS)	(Díaz-Cubilla et al., 2022)

TCS = triclosan; IBU = ibuprofen; SMX = sulfamethoxazole; TMP = trimethoprim; ERY = erythromycin; TC = tetracycline; NOR = norfloxacin; BPA = bisphenol A; E2 = 17β-estradiol; PFAS = per- and poly-fluoroalkyl substances; PFOS = perfluorooctane sulfonate; PFOA = perfluorooctanoic acid; IBU = ibuprofen; CBZ = carbamazepine; TYL = tylosin; MIC = minimum inhibitory concentration; MPs = microplastics; PE-MPs = polyethylene microplastics; aPE-MPs = UV-aged polyethylene microplastics; PS-MPs = polystyrene microplastics; SOD = superoxide dismutase; CAT = catalase.

Table S4. The operational cost of different treatment technologies utilized for EC removal from wastewater matrix.

Treatment Methods	Types	Operational Cost	Advantages	Disadvantages	Ref.
**Physical Methods**	Adsorption	\$0.98 / m^3	Simplicity, low energy consumption, regenerability	Selectivity issues	(Moreira et al., 2021)
	Membrane Technique				
**Chemical Methods**	Ozonation	€300,000 /year	High degradation efficiency	High energy consumption	(Mainardis et al., 2020)
	Electrochemical oxidation	\$0.85 / m^3	Strong controllability	High maintenance costs	(Cui et al., 2017)
	Photocatalytic oxidation	€0.85 −10.36 / m^3	Low energy consumption	Catalyst recovery difficulty	(Segura et al., 2021)
	Ultrasonic oxidation	\$89-15,536 /1000 gallons	Strong penetration	Equipment complexity	(Mahamuni and Adewuyi, 2010)
**Biological Methods**	Aerobic, Anaerobic, Bacteria etc.	€0.17 −0.53/ m^3	Less harmful byproducts	Time consuming	(Moral Pajares et al., 2019; Watkinson et al., 2007)
**Hybrid Methods**	Advanced Oxidation	\$2.2 −4.4/ m^3	Effective for EC	Operating complexity and High operating costs	(Gupta et al., 2021)
	Constructed Wetlands	\$0.02-0.60/ m^3	Low operating costs and Low maintenance	Large land requirement and Long startup period	(Bai et al., 2020; Hunter et al., 2019)

Table S5. Removal efficiency of ECs in different water treatment technology.

**ECs**	**Compounds**	**Water treatment technology**	**Removal efficiency**	**Ref.**
**PPCP**	Paracetamol, diclofenac, naproxen, ibuprofen, benzophenone-3, methylparaben	Ecological filtration	70–99 %	(Pompei et al., 2019)
	Azithromycin, sertraline, tolfenamic acid, and diphenhydramine	Constructed wetlands	>88 %	(Bayati et al., 2021)
	Anti-inflammatory drugs	Anaerobic-anoxic-oxic (A^2/O) wastewater treatment plant	79.4 %	(Ashfaq et al., 2017)
	Diclofenac, ibuprofen	Carbon-based adsorbents	89-96 %	(Guillossou et al., 2020)
	Diclofenac, ibuprofen, paracetamol	Membrane	99.7 %	(Maryam et al., 2020)
	Estrogens	Microalgae	90–95%	(Vassalle et al., 2020)
	17β-estradiol	Microalgae	91%	(Wu et al., 2021)
	SN	Catalytic ozonation	74 %	(Huang et al., 2023)
	SMX	Catalytic ozonation	93.7 ± 1.9 %	(Zhan et al., 2023)
	Ceftiofur Sodium	Photocatalyst	92 %	(Pugazhenthiran et al., 2022)
	Acetaminophen	Photocatalyst	100 %	(Khavar et al., 2023)
	Sulfonamide, Tetracycline	Constructed wetlands	>95 %	(Du et al., 2020)
	Sulfadiazine, Sulfamethoxazole, Sulfadimidine	Constructed wetlands	26.42–84.05 %	(Chen et al., 2020a)

	Sulfamethoxazole, Dimethyl phthalate	Constructed wetlands	94.7 %, 91.8 %	(Li et al., 2023)
**ABX**	CIP, ERY, MET, NOR, SMX, TMP	Adsorption. activated carbon.	88-100%	(Kovalova et al., 2013)
	SMX, TMP	Advanced oxidation processes (AOPs)	98-99%	(Gerrity et al., 2011)
	TMP	Membrane technology	50%	(Sui et al., 2010)
	ERY, SMX, TMP, ERY, SMX, TMP	Membrane technology	93.2-99.3%	(Sahar et al., 2011)
	Anionic surfactants, bisphenol A	Anaerobic digestion	78, 25 and 80 %	(Abril et al., 2020)
**EDCs**	BPA	Adsorption	99.11 %	(Heo et al., 2019)
	E1	Photocatalytic oxidation	94.90 %	(Zhu et al., 2020)
	BPA	Photocatalytic oxidation	91.12 %	(Zhai et al., 2020)
	BPA	Nanofiltration	80%	(Yüksel et al., 2013)
	PCT	Nanofiltration	92-96%	(Nayak et al., 2022)
	E2	Persulfate catalytic oxidation	87 %	(Zhang et al., 2019)
	BPA	Persulfate catalytic oxidation	99.50 %	(Gao et al., 2022)
	BPA, DMP	Fenton-like oxidation	83-85 %	(Luo et al., 2022)
	PCBs	Fenton-like oxidation	77–98 %	(Wang et al., 2022a)

**PFAS**	PFOA, PFBS	Iron-carbon micro-electrolysis	78.12 %, 80.60 %	(Zheng et al., 2023)
	PFOA, PFOS	UV/O_3	27.1 %	(Huang et al., 2016)
	PFOA, PFOS	UV/$NaIO_4$	70 %	(Cao et al., 2010)
	PFOA, PFOS	Coagulation sedimentation	36.12 %	(Sun et al., 2018)
	PFCAs	Composting	80 %	(Abril et al., 2020)
	PFOA	GO	60%	(Lath et al., 2018)
	PFOA	membrane	74.3%	(El Meragawi et al., 2020)
	PFOA, PFBA, PFBS	Bovine serum albumin	23-92%	(Hernandez et al., 2022)
	PFOS	membrane	95%	(Kasula et al., 2024)
**MPs**	Microplastics	Preliminary treatment	6.0 %–58.6 %	(Cheng et al., 2021)
		Primary treatment	19.1 %–99.0 %	(Cheng et al., 2021)
		Secondary treatment	66.7 %–92.6 %	(Cheng et al., 2021)
		Tertiary treatment	72.7 %–99.9 %	(Cheng et al., 2021)
	PS MPs	Electrochemical degradation	89%	(Kiendrebeogo et al., 2021)
	PVC MPs	Electro-Fenton -like system	dechlorination rate 75%	(Miao et al., 2020)
	PS NPs	Ozonation	99.9%	(Li et al., 2022a)

	Mixed MPs	Thermal Fenton system	95.9%	(Hu et al., 2022)
	PS NPs	Photocatalytic degradation	Degradation rate 23.5%	(Domínguez-Jaimes et al., 2021)
	LDPE MPs	Biological degradation	Weight loss 47.22%	(Khan et al., 2023)

Abbreviations: Paracetamol: PCM; Diclofenac: DCF; Naproxen: NPX; Ibuprofen: IBU; Benzophenone-3: BP-3; Methylparaben: MP; Azithromycin: AZM; Sertraline: SER; Tolfenamic acid: TFA; Diphenhydramine: DPH; 17β-estradiol: E2; Sulfonamide: SA; Tetracycline: TC; Sulfadiazine: SDZ; Sulfamethoxazole: SMX; Sulfadimidine: SDM; Dimethyl phthalate: DMP; Ciprofloxacin: CIP; Erythromycin: ERY; Metronidazole: MET; Norfloxacin: NOR; Trimethoprim: TMP; Bisphenol A: BPA; Estrone: E1; Polychlorinated biphenyls: PCBs; Perfluorooctanoic acid: PFOA; Perfluorobutanesulfonic acid: PFBS; Perfluorinated carboxylic acids: PFCA; Perfluorooctanesulfonic acid: PFOS; Low-density polyethylene microplastics: LDPE MPs; Polystyrene microplastics: PS MPs; Polyvinyl chloride microplastics: PVC MPs; Polystyrene nanoparticles: PS NPs; Mixed microplastics: Mixed MPs.

Reference

Abdel Ghani, S.A., El-Sayed, A.A.M., Ibrahim, M.I.A., Ghobashy, M.M., Shreadah, M.A., Shabaka, S., 2022. Characterization and distribution of plastic particles along Alexandria beaches, Mediterranean Coast of Egypt, using microscopy and thermal analysis techniques. Sci. Total Environ. 834, 155363. https://doi.org/10.1016/j.scitotenv.2022.155363.

Abril, C., Santos, J.L., Martín, J., Aparicio, I., Alonso, E., 2020. Occurrence, fate and environmental risk of anionic surfactants, bisphenol A, perfluorinated compounds and personal care products in sludge stabilization treatments. Sci. Total Environ. 711, 135048. https://doi.org/10.1016/j.scitotenv.2019.135048.

Ademollo, N., Spataro, F., Rauseo, J., Pescatore, T., Fattorini, N., Valsecchi, S., et al., 2021. Occurrence, distribution and pollution pattern of legacy and emerging organic pollutants in surface water of the Kongsfjorden (Svalbard, Norway): Environmental contamination, seasonal trend and climate change. Mar. Pollut. Bull. 163, 111900. https://doi.org/10.1016/j.marpolbul.2020.111900.

Adeyinka, G.C., Afolabi, F., Bakare, B.F., 2022. Evaluating the fate and potential health risks of organochlorine pesticides and triclosan in soil, sediment, and water from Asa Dam River, Ilorin Kwara State, Nigeria. Environ. Monit. Assess. 195, 189. https://doi.org/10.1007/s10661-022-10783-5.

Adomat, Y., Grischek, T., 2024. Occurrence, fate and potential risks of pharmaceuticals and personal care products (PPCPs) in Elbe river water during water treatment in Dresden, Germany. Environ. Challenges 15, 100938. https://doi.org/10.1016/j.envc.2024.100938.

Aemig, Q., Hélias, A., Patureau, D., 2021. Impact assessment of a large panel of organic and inorganic

micropollutants released by wastewater treatment plants at the scale of France. Water Res. 188, 116524. https://doi.org/10.1016/j.watres.2020.116524.

Ahmed, F., Tscharke, B., O'Brien, J.W., Hall, W.D., Cabot, P.J., Sowa, P.M., et al., 2023. National Wastewater Reconnaissance of Analgesic Consumption in Australia. Environ. Sci. Technol. 57, 1712–1720. https://doi.org/10.1021/acs.est.2c06691.

Akdemir, T., Gedik, K., 2023. Microplastic emission trends in Turkish primary and secondary municipal wastewater treatment plant effluents discharged into the Sea of Marmara and Black Sea. Environ. Res. 231, 116188. https://doi.org/10.1016/j.envres.2023.116188.

Akdogan, Z., Guven, B., Kideys, A.E., 2023. Microplastic distribution in the surface water and sediment of the Ergene River. Environ. Res. 234, 116500. https://doi.org/10.1016/j.envres.2023.116500.

Al-Mansoori, M., Stephenson, M., Harrad, S., Abdallah, M.A.-E., 2025. Synthetic microplastics in UK tap and bottled water; Implications for human exposure. Emerg. Contam. 11, 100417. https://doi.org/10.1016/j.emcon.2024.100417.

Al Nahian, S., Rakib, M.R.J., Haider, S.M.B., Kumar, R., Mohsen, M., Sharma, P., et al., 2022. Occurrence, spatial distribution, and risk assessment of microplastics in surface water and sediments of Saint Martin Island in the Bay of Bengal. Mar. Pollut. Bull. 179, 113720. https://doi.org/10.1016/j.marpolbul.2022.113720.

Al Nahian, S., Rakib, M.R.J., Kumar, R., Haider, S.M.B., Sharma, P., Idris, A.M., 2023. Distribution, characteristics, and risk assessments analysis of microplastics in shore sediments and surface water of Moheshkhali channel of Bay of Bengal, Bangladesh. Sci. Total Environ. 855, 158892. https://doi.org/10.1016/j.scitotenv.2022.158892.

Alam, M.J., Shammi, M., Tareq, S.M., 2023. Distribution of microplastics in shoreline water and

sediment of the Ganges River Basin to Meghna Estuary in Bangladesh. Ecotoxicol. Environ. Saf. 266, 115537. https://doi.org/10.1016/j.ecoenv.2023.115537.

Alavian Petroody, S.S., Hashemi, S.H., van Gestel, C.A.M., 2020. Factors affecting microplastic retention and emission by a wastewater treatment plant on the southern coast of Caspian Sea. Chemosphere 261, 128179. https://doi.org/10.1016/j.chemosphere.2020.128179.

Alexa, E.T., Bernal-Romero del Hombre Bueno, M.D., González, R., Sánchez, A.V., García, H., Prats, D., 2022. Occurrence and removal of priority substances and contaminants of emerging concern at the WWTP of Benidorm (Spain). Water 14, 3284. https://doi.org/10.3390/w14244129.

Ali, A.M., Higgins, C.P., Alarif, W.M., Al-Lihaibi, S.S., Ghandourah, M., Kallenborn, R., 2021. Per- and polyfluoroalkyl substances (PFASs) in contaminated coastal marine waters of the Saudi Arabian Red Sea: A baseline study. Environ. Sci. Pollut. Res. 28, 2791–2803. https://doi.org/10.1007/s11356-020-09897-5.

Almaiman, L., Aljomah, A., Bineid, M., Aljeldah, F.M., Aldawsari, F., Liebmann, B., et al., 2021. The occurrence and dietary intake related to the presence of microplastics in drinking water in Saudi Arabia. Environ. Monit. Assess. 193, 390. https://doi.org/10.1007/s10661-021-09132-9.

An, L., Cui, T., Zhang, Y., Liu, H., 2022. A case study on small-size microplastics in water and snails in an urban river. Sci. Total Environ. 847, 157461. https://doi.org/10.1016/j.scitotenv.2022.157461.

Anagnostopoulpou, K., Nannou, C., Aschonitis, V.G., Lambropoulou, D.A., 2022. Screening of pesticides and emerging contaminants in eighteen Greek lakes by using target and non-target HRMS approaches: Occurrence and ecological risk assessment. Sci. Total Environ. 849, 157887. https://doi.org/10.1016/j.scitotenv.2022.157887.

Andrews, D.Q., Naidenko, O.V., 2020. Population-wide exposure to per- and polyfluoroalkyl substances

from drinking water in the United States. Environ. Sci. Technol. Lett. 7, 931–936. https://doi.org/10.1021/acs.estlett.0c00713.

Angeles, L.F., Islam, S., Aldstadt, J., Saqeeb, K.N., Alam, M., Khan, M.A., et al., 2020. Retrospective suspect screening reveals previously ignored antibiotics, antifungal compounds, and metabolites in Bangladesh surface waters. Sci. Total Environ. 712, 136285. https://doi.org/10.1016/j.scitotenv.2019.136285.

Anim, A.K., Thompson, K., Duodu, G.O., Tscharke, B., Birch, G., Goonetilleke, A., et al., 2020. Pharmaceuticals, personal care products, food additive and pesticides in surface waters from three Australian east coast estuaries (Sydney, Yarra and Brisbane). Mar. Pollut. Bull. 153, 111014. https://doi.org/10.1016/j.marpolbul.2020.111014.

Arregocés-Garcés, R., Garcés-Ordóñez, O., Vivas-Aguas, L.-J., Canals, M., 2024. Microplastics transfer from a malfunctioning municipal wastewater oxidation pond into a marine protected area in the Colombian Caribbean. Reg. Stud. Mar. Sci. 69, 103361. https://doi.org/10.1016/j.rsma.2023.103361.

Arsand, J.B., Hoff, R.B., Jank, L., Bussamara, R., Dallegrave, A., Bento, F.M., et al., 2020. Presence of antibiotic resistance genes and its association with antibiotic occurrence in Dilúvio River in southern Brazil. Sci. Total Environ. 738, 139781. https://doi.org/10.1016/j.scitotenv.2020.139781.

Ashfaq, M., Li, Y., Wang, Y., Chen, W., Wang, H., Chen, X., et al., 2017. Occurrence, fate, and mass balance of different classes of pharmaceuticals and personal care products in an anaerobic-anoxic-oxic wastewater treatment plant in Xiamen, China. Water Res. 123, 655–667. https://doi.org/10.1016/j.watres.2017.07.014.

Ashfaq, M., Li, Y., Zubair, M., Ur Rehman, M.S., Sumrra, S.H., Nazar, M.F., et al., 2023. Occurrence and risk evaluation of endocrine-disrupting chemicals in wastewater and surface water of Lahore, Pakistan. Environ. Geochem. Health 45, 4837–4851. https://doi.org/10.1007/s10653-023-01527-6.

Astuti, M.P., Notodarmojo, S., Priadi, C.R., Padhye, L.P., 2023. Contaminants of emerging concerns (CECs) in a municipal wastewater treatment plant in Indonesia. Environ. Sci. Pollut. Res. 30, 21512–21532. https://doi.org/10.1007/s11356-022-23567-8.

Aydin, S., Ulvi, A., Aydin, M.E., 2024. Occurrence, characteristics, and risk assessment of microplastics and polycyclic aromatic hydrocarbons associated with microplastics in surface water and sediments of the Konya Closed Basin, Turkey. Environ. Sci. Pollut. Res. 31, 57989–58009. https://doi.org/10.1007/s11356-024-35029-4.

Äystö, L., Vieno, N., Fjäder, P., Mehtonen, J., Nystén, T., 2023. Hospitals and households as primary emission sources for risk-posing pharmaceuticals in municipal wastewater. Ecotoxicol. Environ. Saf. 262, 115149. https://doi.org/10.1016/j.ecoenv.2023.115149.

Azzi, M., Ravier, S., Elkak, A., Coulomb, B., Boudenne, J.-L., 2021. Fast UHPLC-MS/MS for the simultaneous determination of azithromycin, erythromycin, fluoxetine and sotalol in surface water samples. Appl. Sci. 11, 2456.

Babayev, M., Capozzi, S.L., Miller, P., McLaughlin, K.R., Medina, S.S., Byrne, S., et al., 2022. PFAS in drinking water and serum of the people of a southeast Alaska community: A pilot study. Environ. Pollut. 305, 119246. https://doi.org/10.1016/j.envpol.2022.119246.

Bai, X., Son, Y., 2021. Perfluoroalkyl substances (PFAS) in surface water and sediments from two urban watersheds in Nevada, USA. Sci. Total Environ. 751, 141622.

https://doi.org/10.1016/j.scitotenv.2020.141622.

Bai, X., Zhu, X., Jiang, H., Wang, Z., He, C., Sheng, L., et al., 2020. Purification effect of sequential constructed wetland for the polluted water in urban river. *Water* 12, 3564. https://doi.org/10.3390/w12041054.

Bakare, B.F., Adeyinka, G.C., 2022. Occurrence and fate of triclosan and triclocarban in selected wastewater systems across Durban Metropolis, KwaZulu-Natal, South Africa. *Int. J. Environ. Res. Public Health* 19, 5246. https://doi.org/10.3390/ijerph19116769.

Bandara, R.M.L.S., Perera, M.D.D., Gomes, P.I.A., Yan, X.-F., 2023. Profiling microplastic pollution in surface water bodies in the most urbanized city of Sri Lanka and its suburbs to understand the underlying factors. Water Air Soil Pollut. 234, 157. https://doi.org/10.1007/s11270-023-06168-0.

Bayati, M., Ho, T.L., Vu, D.C., Wang, F., Rogers, E., Cuvellier, C., et al., 2021. Assessing the efficiency of constructed wetlands in removing PPCPs from treated wastewater and mitigating the ecotoxicological impacts. Int. J. Hyg. Environ. Health 231, 113664. https://doi.org/10.1016/j.ijheh.2020.113664.

Ben, Y., Hu, M., Zhang, X., Wu, S., Wong, M.H., Wang, M., et al., 2020. Efficient detection and assessment of human exposure to trace antibiotic residues in drinking water. Water Res. 175, 115699. https://doi.org/10.1016/j.watres.2020.115699.

Bentaallah, M.E.A., Baghdadi, D., Gündoğdu, S., Megharbi, A., Taibi, N.-E., Büyükdeveci, F., 2024. Assessment of microplastic abundance and impact on recreational beaches along the western Algerian coastline. Mar. Pollut. Bull. 199, 116007. https://doi.org/10.1016/j.marpolbul.2023.116007.

Berov, D., Klayn, S., 2020. Microplastics and floating litter pollution in Bulgarian Black Sea coastal waters. Mar. Pollut. Bull. 156, 111225. https://doi.org/10.1016/j.marpolbul.2020.111225.

Blankson, E.R., Tetteh, P.N., Oppong, P., Gbogbo, F., 2022. Microplastics prevalence in water, sediment and two economically important species of fish in an urban riverine system in Ghana. PLoS ONE 17, e0263196. https://doi.org/10.1371/journal.pone.0263196.

Brožová, K., Halfar, J., Čabanová, K., Motyka, O., Drabinová, S., Hanus, P., et al., 2023. The first evidence of microplastic occurrence in mine water: The largest black coal mining area in the Czech Republic. Water Res. 244, 120538. https://doi.org/10.1016/j.watres.2023.120538.

Bujaczek, T., Kolter, S., Locky, D., Ross, M.S., 2021. Characterization of microplastics and anthropogenic fibers in surface waters of the North Saskatchewan River, Alberta, Canada. FACETS 6, 26–43. https://doi.org/10.1139/facets-2020-0057.

Campanale, C., Stock, F., Massarelli, C., Kochleus, C., Bagnuolo, G., Reifferscheid, G., et al., 2020. Microplastics and their possible sources: The example of Ofanto river in southeast Italy. Environ. Pollut. 258, 113284. https://doi.org/10.1016/j.envpol.2019.113284.

Cantoni, B., Bergna, G., Baldini, E., Malpei, F., Antonelli, M., 2024. PFAS in textile wastewater: An integrated scenario analysis for interventions prioritization to reduce environmental risk. Process Saf. Environ. Prot. 183, 437–445. https://doi.org/10.1016/j.psep.2024.01.005.

Cao, M.H., Wang, B.B., Yu, H.S., Wang, L.L., Yuan, S.H., Chen, J., 2010. Photochemical decomposition of perfluorooctanoic acid in aqueous periodate with VUV and UV light irradiation. J. Hazard. Mater. 179, 1143–1146. https://doi.org/10.1016/j.jhazmat.2010.02.030.

Capparelli, M.V., Molinero, J., Moulatlet, G.M., Barrado, M., Prado-Alcívar, S., Cabrera, M., et al., 2021. Microplastics in rivers and coastal waters of the province of Esmeraldas, Ecuador. Mar. Pollut.

Bull. 173, 113067. https://doi.org/10.1016/j.marpolbul.2021.113067.

Castaño-Trias, M., Rodríguez-Mozaz, S., Verlicchi, P., Buttiglieri, G., 2024. Selection of pharmaceuticals of concern in reclaimed water for crop irrigation in the Mediterranean area. J. Hazard. Mater. 466, 133538. https://doi.org/10.1016/j.jhazmat.2024.133538.

Castro, R.O., Silva, M.L.d., Marques, M.R.C., Araújo, F.V.d., 2020. Spatio-temporal evaluation of macro, meso and microplastics in surface waters, bottom and beach sediments of two embayments in Niterói, RJ, Brazil. Mar. Pollut. Bull. 160, 111537. https://doi.org/10.1016/j.marpolbul.2020.111537.

Cerón-Vivas, A., Peñuela Mesa, G.A., 2024. Environmental risk assessment of pharmaceutical pollutants in the Oro River Sub-basin (Colombia). Environ. Res. 252, 118951. https://doi.org/10.1016/j.envres.2024.118951.

Chakraborty, P., Shappell, N.W., Mukhopadhyay, M., Onanong, S., Rex, K.R., Snow, D., 2021. Surveillance of plasticizers, bisphenol A, steroids and caffeine in surface water of River Ganga and Sundarban wetland along the Bay of Bengal: Occurrence, sources, estrogenicity screening and ecotoxicological risk assessment. Water Res. 190, 116668. https://doi.org/10.1016/j.watres.2020.116668.

Chandrajith, R., Zwiener, C., Daniel, C., Amann, K., Nanayakkara, N., Barth, J.A.C., 2025. Screening of micro-organic compounds in groundwater from areas with chronic kidney disease of unclear aetiology (CKDu) in the dry zone of Sri Lanka. Exposure Health 17, 167–176. https://doi.org/10.1007/s12403-024-00651-7.

Chaves, M.d.J.S., Barbosa, S.C., Malinowski, M.d.M., Volpato, D., Castro, Í.B., Franco, T.C.R.d.S., et al., 2020. Pharmaceuticals and personal care products in a Brazilian wetland of international

importance: Occurrence and environmental risk assessment. Sci. Total Environ. 734, 139374. https://doi.org/10.1016/j.scitotenv.2020.139374.

Chaves, M.d.J.S., Barbosa, S.C., Primel, E.G., 2021. Emerging contaminants in Brazilian aquatic environment: Identifying targets of potential concern based on occurrence and ecological risk. Environ. Sci. Pollut. Res. 28, 67528–67543. https://doi.org/10.1007/s11356-021-15245-y.

Chen, J., Tong, T., Jiang, X., Xie, S., 2020a. Biodegradation of sulfonamides in both oxic and anoxic zones of vertical flow constructed wetland and the potential degraders. Environ. Pollut. 265, 115040. https://doi.org/10.1016/j.envpol.2020.115040.

Chen, P., Zhong, Y., Chen, K., Guo, C., Gong, J., Wang, D., et al., 2020b. The impact of discharge reduction activities on the occurrence of contaminants of emerging concern in surface water from the Pearl River. Environ. Sci. Pollut. Res. 27, 30378–30389. https://doi.org/10.1007/s11356-020-09295-x.

Chen, Y., Zhang, H., Liu, Y., Bowden, J.A., Tolaymat, T.M., Townsend, T.G., et al., 2023. Evaluation of per- and polyfluoroalkyl substances (PFAS) in leachate, gas condensate, stormwater and groundwater at landfills. Chemosphere 318, 137903. https://doi.org/10.1016/j.chemosphere.2023.137903.

Cheng, Y.L., Kim, J.-G., Kim, H.-B., Choi, J.H., Fai Tsang, Y., Baek, K., 2021. Occurrence and removal of microplastics in wastewater treatment plants and drinking water purification facilities: A review. Chem. Eng. J. 410, 128381. https://doi.org/10.1016/j.cej.2020.128381.

Chepchirchir, R., Mwalimu, R., Tanui, I., Kiprop, A., Krauss, M., Brack, W., et al., 2024. Occurrence, removal and risk assessment of chemicals of emerging concern in selected rivers and wastewater treatment plants in western Kenya. Sci. Total Environ. 948,

174982. https://doi.org/10.1016/j.scitotenv.2024.174982.

Chernova, E., Zhakovskaya, Z., Berezina, N., 2021. Occurrence of pharmaceuticals in the Eastern Gulf of Finland (Russia). Environ. Sci. Pollut. Res. 28, 68871–68884. https://doi.org/10.1007/s11356-021-15250-1.

Cipriani-Avila, I., Molinero, J., Cabrera, M., Medina-Villamizar, E.J., Capparelli, M.V., Jara-Negrete, E., et al., 2023. Occurrence of emerging contaminants in surface water bodies of a coastal province in Ecuador and possible influence of tourism decline caused by COVID-19 lockdown. Sci. Total Environ. 866, 161340. https://doi.org/10.1016/j.scitotenv.2022.161340.

Close, M.E., Humphries, B., Northcott, G., 2021. Outcomes of the first combined national survey of pesticides and emerging organic contaminants (EOCs) in groundwater in New Zealand 2018. Sci. Total Environ. 754, 142005. https://doi.org/10.1016/j.scitotenv.2020.142005.

Cooney, J., Lenczewski, M., Leal-Bautista, R.M., Tucker, K., Davis, M., Rodriguez, J., 2023. Analysis of sunscreens and antibiotics in groundwater during the COVID-19 pandemic in the Riviera Maya, Mexico. Sci. Total Environ. 894, 164820. https://doi.org/10.1016/j.scitotenv.2023.164820.

Cruz-López, A., Dávila-Pórcel, R.A., de León-Gómez, H., Rodríguez-Martínez, J.M., Suárez-Vázquez, S.I., Cardona-Benavides, A., et al., 2020. Exploratory study on the presence of bisphenol A and bis(2-ethylhexyl) phthalate in the Santa Catarina River in Monterrey, N.L., Mexico. Environ. Monit. Assess. 192, 488. https://doi.org/10.1007/s10661-020-08446-4.

Cui, T., Zhang, Y., Han, W., Li, J., Sun, X., Shen, J., et al., 2017. Advanced treatment of triazole fungicides discharged water in pilot scale by integrated system: Enhanced electrochemical oxidation, upflow biological aerated filter and electrodialysis. Chem. Eng. J. 315, 335–

344. https://doi.org/10.1016/j.cej.2017.01.039.
Cunsolo, S., Williams, J., Hale, M., Read, D.S., Couceiro, F., 2021. Optimising sample preparation for FTIR-based microplastic analysis in wastewater and sludge samples: Multiple digestions. Anal. Bioanal. Chem. 413, 3789–3799. https://doi.org/10.1007/s00216-021-03331-6.
Currell, M., Northby, N., Netherway, P., 2024. Examining changes in groundwater PFAS contamination from legacy landfills over a three-year period at Australia's largest urban renewal site. Chemosphere 352, 141345. https://doi.org/10.1016/j.chemosphere.2024.141345.
da Silva, B.F., Aristizabal-Henao, J.J., Aufmuth, J., Awkerman, J., Bowden, J.A., 2022. Survey of per- and polyfluoroalkyl substances (PFAS) in surface water collected in Pensacola, FL. Heliyon 8, e10239. https://doi.org/10.1016/j.heliyon.2022.e10239.
Datel, J.V., Hrabankova, A., 2020. Pharmaceuticals load in the Svihov Water Reservoir (Czech Republic) and impacts on quality of treated drinking water. Water 12, 2020. https://doi.org/10.3390/w12051387.
de Carvalho, A.R., Garcia, F., Riem-Galliano, L., Tudesque, L., Albignac, M., ter Halle, A., et al., 2021. Urbanization and hydrological conditions drive the spatial and temporal variability of microplastic pollution in the Garonne River. Sci. Total Environ. 769, 144479. https://doi.org/10.1016/j.scitotenv.2020.144479.
de Moraes, N.G., Olivatto, G.P., Lourenço, F.M.d.O., Lourenço, A.L.A., Garcia, G.M., Pimpinato, R.F., et al., 2024. Contamination by microplastics and sorbed organic pollutants in the surface waters of the Tietê River, São Paulo-SP, Brazil. Heliyon 10, e36047. https://doi.org/10.1016/j.heliyon.2024.e36047.
de Oliveira Santos, A.D., do Nascimento, M.T.L., Sanson, A.L., dos Santos, R.F., Felix, L.C., da Silva

de Freitas, A., et al., 2022. Pharmaceuticals, natural and synthetic hormones and phenols in sediments from an eutrophic estuary, Jurujuba Sound, Guanabara Bay, Brazil. Mar. Pollut. Bull. 184, 114176. https://doi.org/10.1016/j.marpolbul.2022.114176.

de Rezende, A.T., Mounteer, A.H., 2023. Ecological risk assessment of pharmaceuticals and endocrine disrupting compounds in Brazilian surface waters. Environ. Pollut. 338, 122628. https://doi.org/10.1016/j.envpol.2023.122628.

Dehm, J., Singh, S., Ferreira, M., Piovano, S., Fick, J., 2021. Screening of pharmaceuticals in coastal waters of the southern coast of Viti Levu in Fiji, South Pacific. Chemosphere 276, 130161. https://doi.org/10.1016/j.chemosphere.2021.130161.

Diaz-Sosa, V.R., Tapia-Salazar, M., Wanner, J., Cardenas-Chavez, D.L., 2020. Monitoring and ecotoxicity assessment of emerging contaminants in wastewater discharge in the City of Prague (Czech Republic). Water 12, 2020. https://doi.org/10.3390/w12041079.

Díaz-Cubilla, M., Letón, P., Luna-Vázquez, C., Marrón-Romera, M., Boltes, K., 2022. Effect of carbamazepine, ibuprofen, triclosan and sulfamethoxazole on anaerobic bioreactor performance: Combining cell damage, ecotoxicity and chemical information. Toxics 10, 137. https://doi.org/10.3390/toxics10040137

Ding, G., Zhang, J., Chen, Y., Wang, L., Wang, M., Xiong, D., et al., 2013. Combined effects of PFOS and PFOA on zebrafish (Danio rerio) embryos. Arch. Environ. Contam. Toxicol. 64, 668–675. https://doi.org/10.1007/s00244-012-9864-2

Dilshad, A., Taneez, M., Younas, F., Jabeen, A., Rafiq, M.T., Fatimah, H., 2022. Microplastic pollution in the surface water and sediments from Kallar Kahar wetland, Pakistan: Occurrence, distribution, and characterization by ATR-FTIR. Environ. Monit. Assess. 194,

511. https://doi.org/10.1007/s10661-022-10171-z.

do Nascimento, R.F., de Carvalho Filho, J.A.A., Napoleão, D.C., Ribeiro, B.G., da Silva Pereira Cabral, J.J., de Paiva, A.L.R., 2023. Presence of non-steroidal anti-inflammatories in Brazilian semiarid waters. Water Air Soil Pollut. 234, 225. https://doi.org/10.1007/s11270-023-06239-2.

Domínguez-Jaimes, L.P., Cedillo-González, E.I., Luévano-Hipólito, E., Acuña-Bedoya, J.D., Hernández-López, J.M., 2021. Degradation of primary nanoplastics by photocatalysis using different anodized TiO2 structures. J. Hazard. Mater. 413, 125452. https://doi.org/10.1016/j.jhazmat.2021.125452.

Du, L., Zhao, Y., Wang, C., Zhang, H., Chen, Q., Zhang, X., et al., 2020. Removal performance of antibiotics and antibiotic resistance genes in swine wastewater by integrated vertical-flow constructed wetlands with zeolite substrate. Sci. Total Environ. 721, 137765. https://doi.org/10.1016/j.scitotenv.2020.137765.

Durcik, M., Grobin, A., Roškar, R., Trontelj, J., Peterlin Mašič, L., 2023. Estrogenic potency of endocrine disrupting chemicals and their mixtures detected in environmental waters and wastewaters. Chemosphere 330, 138712. https://doi.org/10.1016/j.chemosphere.2023.138712.

Duru, C.I., Kang, D.H., Sherchan, S.P., 2024. The trends of per- and polyfluoroalkyl substances (PFAS) in drinking water systems in Maryland, United States. Sci. Total Environ. 957, 177152. https://doi.org/10.1016/j.scitotenv.2024.177152.

Dvorakova, D., Jurikova, M., Svobodova, V., Parizek, O., Kozisek, F., Kotal, F., et al., 2023. Complex monitoring of perfluoroalkyl substances (PFAS) from tap drinking water in the Czech Republic. Water Res. 247, 120764. https://doi.org/10.1016/j.watres.2023.120764.

Ebele, A.J., Oluseyi, T., Drage, D.S., Harrad, S., Abou-Elwafa Abdallah, M., 2020. Occurrence, seasonal

variation and human exposure to pharmaceuticals and personal care products in surface water, groundwater and drinking water in Lagos State, Nigeria. Emerg. Contam. 6, 124–132. https://doi.org/10.1016/j.emcon.2020.02.004.

El Meragawi, S., Akbari, A., Hernandez, S., Mirshekarloo, M.S., Bhattacharyya, D., Tanksale, A., et al., 2020. Enhanced permselective separation of per-fluorooctanoic acid in graphene oxide membranes by a simple PEI modification. J. Mater. Chem. A 8, 24800–24811. https://doi.org/10.1039/D0TA06523D.

Elles-Pérez, C., Guzman-Tordecilla, M., Ramos, Y., Castillo-Ramírez, M., Moreno-Ríos, A., Garzón-Rodríguez, C., et al., 2024. Assessment of water quality and emerging pollutants in two fish species from the Mallorquín Swamp in the Colombian Caribbean. Heliyon 10, e39005. https://doi.org/10.1016/j.heliyon.2024.e39005.

Emam, T.E., Souaya, E.R., Ibrahim, M.B.M., Mahmoud, S.A., 2023. Advanced removal of pesticides, herbicides, and pharmaceutical residues from surface water. Environ. Technol. 44, 3466–3478. https://doi.org/10.1080/09593330.2022.2064234.

Emnet, P., Mahaliyana, A.S., Northcott, G., Gaw, S., 2020. Organic micropollutants in wastewater effluents and the receiving coastal waters, sediments, and biota of Lyttelton Harbour (Te Whakaraupō), New Zealand. Arch. Environ. Contam. Toxicol. 79, 461–477. https://doi.org/10.1007/s00244-020-00760-9.

Erdem, İ.Ç., Yurtsever, M., Şahin, F., 2024. Determination of microplastics in drinking water treatment plants and tap water in Kocaeli, Turkey. Urban Water J. 21, 941–952. https://doi.org/10.1080/1573062X.2024.2395814.

Fabregat-Safont, D., Botero-Coy, A.M., Nieto-Juárez, J.I., Torres-Palma, R.A., Hernández, F., 2023.

Searching for pharmaceutically active products and metabolites in environmental waters of Peru by HRMS-based screening: Proposal for future monitoring and environmental risk assessment. Chemosphere 337, 139375. https://doi.org/10.1016/j.chemosphere.2023.139375.

Falahudin, D., Cordova, M.R., Sun, X., Yogaswara, D., Wulandari, I., Hindarti, D., et al., 2020. The first occurrence, spatial distribution and characteristics of microplastic particles in sediments from Banten Bay, Indonesia. Sci. Total Environ. 705, 135304. https://doi.org/10.1016/j.scitotenv.2019.135304.

Fan, D., Yin, W., Gu, W., Liu, M., Liu, J., Wang, Z., et al., 2021a. Occurrence, spatial distribution and risk assessment of high concern endocrine-disrupting chemicals in Jiangsu Province, China. Chemosphere 285, 131396. https://doi.org/10.1016/j.chemosphere.2021.131396.

Fan, J., Zou, L., Zhao, G., 2021b. Microplastic abundance, distribution, and composition in the surface water and sediments of the Yangtze River along Chongqing City, China. J. Soils Sediments 21, 1840–1851. https://doi.org/10.1007/s11368-021-02902-5.

Fan, L., Mohseni, A., Schmidt, J., Evans, B., Murdoch, B., Gao, L., 2023. Efficiency of lagoon-based municipal wastewater treatment in removing microplastics. Sci. Total Environ. 876, 162714. https://doi.org/10.1016/j.scitotenv.2023.162714.

Fan, Y., Zheng, J., Deng, L., Rao, W., Zhang, Q., Liu, T., et al., 2022. Spatiotemporal dynamics of microplastics in an urban river network area. Water Res. 212, 118116. https://doi.org/10.1016/j.watres.2022.118116.

Fardullah, M., Hossain, M.T., Islam, M.S., Islam, M.R., Rahman, M.R., Akther, K., et al., 2024. Occurrence and spatial distribution of microplastics in water and sediments of Hatiya Island, Bangladesh and their risk assessment. J. Environ. Manag. 370, 122697.

https://doi.org/10.1016/j.jenvman.2024.122697.

Fernandes, M.J., Paíga, P., Silva, A., Llaguno, C.P., Carvalho, M., Vázquez, F.M., et al., 2020. Antibiotics and antidepressants occurrence in surface waters and sediments collected in the north of Portugal. Chemosphere 239, 124729. https://doi.org/10.1016/j.chemosphere.2019.124729.

Ferraz, M., Bauer, A.L., Valiati, V.H., Schulz, U.H., 2020. Microplastic concentrations in raw and drinking water in the Sinos River, Southern Brazil. Water 12, 2020. https://doi.org/10.3390/w12113115.

Fiedler, H., Vega-Bustillos, L., Arias-Pastrano, J., Pérez-Aldás, L.V., Castro-Díaz, J., 2024. Snapshot survey of the presence of perfluoroalkyl substances in products, articles, and the environment in Ecuador. Eng. 37, 49–61. https://doi.org/10.1016/j.eng.2024.01.013.

Fonseca, E., Hernández, F., Ibáñez, M., Rico, A., Pitarch, E., Bijlsma, L., 2020. Occurrence and ecological risks of pharmaceuticals in a Mediterranean river in Eastern Spain. Environ. Int. 144, 106004. https://doi.org/10.1016/j.envint.2020.106004.

Franco, A.A., Arellano, J.M., Albendín, G., Rodríguez-Barroso, R., Quiroga, J.M., Coello, M.D., 2021. Microplastic pollution in wastewater treatment plants in the city of Cádiz: Abundance, removal efficiency and presence in receiving water body. Sci. Total Environ. 776, 145795. https://doi.org/10.1016/j.scitotenv.2021.145795.

Frank, Y., Ershova, A., Batasheva, S., Vorobiev, E., Rakhmatullina, S., Vorobiev, D., et al., 2022. Microplastics in freshwater: A focus on the Russian inland waters. Water 14, 2022.

Frank, Y.A., Vorobiev, E.D., Vorobiev, D.S., Trifonov, A.A., Antsiferov, D.V., Soliman Hunter, T., et al., 2021. Preliminary screening for microplastic concentrations in the surface water of the Ob and Tom Rivers in Siberia, Russia. Sustainability 13, 2021.

Fredriksson, F., Eriksson, U., Kärrman, A., Yeung, L.W.Y., 2022. Per- and polyfluoroalkyl substances (PFAS) in sludge from wastewater treatment plants in Sweden—First findings of novel fluorinated copolymers in Europe including temporal analysis. Sci. Total Environ. 846, 157406. https://doi.org/10.1016/j.scitotenv.2022.157406.

Gallego-Ríos, S.E., Peñuela, G.A., 2021. Evaluation of ibuprofen and diclofenac in the main rivers of Colombia and striped catfish *Pseudoplatystoma magdaleniatum*. Environ. Monit. Assess. 193, 210. https://doi.org/10.1007/s10661-021-08922-5.

Gao, Y., Chen, Y., Song, T., Su, R., Luo, J., 2022. Activated peroxymonosulfate with ferric chloride-modified biochar to degrade bisphenol A: Characteristics, influencing factors, reaction mechanism and reuse performance. Sep. Purif. Technol. 300, 121857. https://doi.org/10.1016/j.seppur.2022.121857.

Garcés-Ordóñez, O., Saldarriaga-Vélez, J.F., Espinosa-Díaz, L.F., Patiño, A.D., Cusba, J., Canals, M., et al., 2022. Microplastic pollution in water, sediments and commercial fish species from Ciénaga Grande de Santa Marta lagoon complex, Colombian Caribbean. Sci. Total Environ. 829, 154643. https://doi.org/10.1016/j.scitotenv.2022.154643.

Gebbink, W.A., van Leeuwen, S.P.J., 2020. Environmental contamination and human exposure to PFASs near a fluorochemical production plant: Review of historic and current PFOA and GenX contamination in the Netherlands. Environ. Int. 137, 105583. https://doi.org/10.1016/j.envint.2020.105583.

Gerrity, D., Gamage, S., Holady, J.C., Mawhinney, D.B., Quiñones, O., Trenholm, R.A., et al., 2011. Pilot-scale evaluation of ozone and biological activated carbon for trace organic contaminant mitigation and disinfection. Water Res. 45, 2155–2165.

https://doi.org/10.1016/j.watres.2010.12.031.

Gevao, B., Uddin, S., Krishnan, D., Rajagopalan, S., Habibi, N., 2022. Antibiotics in wastewater: Baseline of the influent and effluent streams in Kuwait. Toxics 10, 2022.

Gewurtz, S.B., Auyeung, A.S., De Silva, A.O., Teslic, S., Smyth, S.A., 2024. Per- and polyfluoroalkyl substances (PFAS) in Canadian municipal wastewater and biosolids: Recent patterns and time trends 2009 to 2021. Sci. Total Environ. 912, 168638. https://doi.org/10.1016/j.scitotenv.2023.168638.

Gobelius, L., Glimstedt, L., Olsson, J., Wiberg, K., Ahrens, L., 2023. Mass flow of per- and polyfluoroalkyl substances (PFAS) in a Swedish municipal wastewater network and wastewater treatment plant. Chemosphere 336, 139182. https://doi.org/10.1016/j.chemosphere.2023.139182.

Göckener, B., Fliedner, A., Weinfurtner, K., Rüdel, H., Badry, A., Koschorreck, J., 2023. Tracking down unknown PFAS pollution—The direct TOP assay in spatial monitoring of surface waters in Germany. Sci. Total Environ. 898, 165425. https://doi.org/10.1016/j.scitotenv.2023.165425.

Grbić, J., Helm, P., Athey, S., Rochman, C.M., 2020. Microplastics entering northwestern Lake Ontario are diverse and linked to urban sources. Water Res. 174, 115623. https://doi.org/10.1016/j.watres.2020.115623.

Grini, H., Metallaoui, S., Rangel-Buitrago, N., Hadef, A., González-Fernández, D., Bensouilah, M., 2024. Persistence and potential increasing accumulation of microplastic pollution on the Skikda coast (northeastern Algeria). Mar. Pollut. Bull. 209, 117314. https://doi.org/10.1016/j.marpolbul.2024.117314.

Grobin, A., Roškar, R., Trontelj, J., 2024. The environmental occurrence, fate, and risks of 25 endocrine

disruptors in Slovenian waters. Sci. Total Environ. 906, 167245. https://doi.org/10.1016/j.scitotenv.2023.167245.

Grung, M., Hjermann, D.Ø., Rundberget, T., Bæk, K., Thomsen, C., Knutsen, H.K., et al., 2024. Low levels of per- and polyfluoroalkyl substances (PFAS) detected in drinking water in Norway, but elevated concentrations found near known sources. Sci. Total Environ. 947, 174550. https://doi.org/10.1016/j.scitotenv.2024.174550.

Guillossou, R., Le Roux, J., Mailler, R., Pereira-Derome, C.S., Varrault, G., Bressy, A., et al., 2020. Influence of dissolved organic matter on the removal of 12 organic micropollutants from wastewater effluent by powdered activated carbon adsorption. Water Res. 172, 115487. https://doi.org/10.1016/j.watres.2020.115487.

Guo, W., Li, J., Luo, M., Mao, Y., Yu, X., Elskens, M., et al., 2022. Estrogenic activity and ecological risk of steroids, bisphenol A and phthalates after secondary and tertiary sewage treatment processes. Water Res. 214, 118189. https://doi.org/10.1016/j.watres.2022.118189.

Gupta, S., Gomaa, H., Ray, M.B., 2021. Performance characterization of a hybrid adsorptive-photocatalytic (APC) oscillatory membrane reactor for micropollutant removal. Sep. Purif. Technol. 279, 119706. https://doi.org/10.1016/j.seppur.2021.119706.

Haenni, M., Dagot, C., Chesneau, O., Bibbal, D., Labanowski, J., Vialette, M., et al., 2022. Environmental contamination in a high-income country (France) by antibiotics, antibiotic-resistant bacteria, and antibiotic resistance genes: Status and possible causes. Environ. Int. 159, 107047. https://doi.org/10.1016/j.envint.2021.107047.

Hain, E., He, K., Batista-Andrade, J.A., Feerick, A., Tarnowski, M., Timm, A., et al., 2023. Geospatial and co-occurrence analysis of antibiotics, hormones, and UV filters in the Chesapeake Bay

(USA) to confirm inputs from wastewater treatment plants, septic systems, and animal feeding operations. J. Hazard. Mater. 460, 132405. https://doi.org/10.1016/j.jhazmat.2023.132405.

Hajiouni, S., Mohammadi, A., Ramavandi, B., Arfaeinia, H., De-la-Torre, G.E., Tekle-Röttering, A., et al., 2022. Occurrence of microplastics and phthalate esters in urban runoff: A focus on the Persian Gulf coastline. Sci. Total Environ. 806, 150559. https://doi.org/10.1016/j.scitotenv.2021.150559.

Halfar, J., Brožová, K., Placová, K., Kyncl, M., 2023. Determining the presence of micro-particles in drinking water in the Czech Republic—An exploratory study focusing on microplastics and additives. Eng. Proc. 57, 2023. https://doi.org/10.3390/engproc2023057016.

Halfar, J., Heviánková, S., Brožová, K., Čabanová, K., Valigůrová, A., Motyka, O., 2024. Microplastic contamination in Czech drinking water: Insights from comprehensive monitoring. Environ. Sci. Eur. 36, 213. https://doi.org/10.1186/s12302-024-01036-y.

Haque, A., Holsen, T.M., Baki, A.B.M., 2024. Distribution and risk assessment of microplastic pollution in a rural river system near a wastewater treatment plant, hydro-dam, and river confluence. Sci. Rep. 14, 6006. https://doi.org/10.1038/s41598-024-56730-x.

Haque, M.R., Ali, M.M., Ahmed, W., Siddique, M.A.B., Akbor, M.A., Islam, M.S., et al., 2023. Assessment of microplastics pollution in aquatic species (fish, crab, and snail), water, and sediment from the Buriganga River, Bangladesh: An ecological risk appraisal. Sci. Total Environ. 857, 159344. https://doi.org/10.1016/j.scitotenv.2022.159344.

Harley-Nyang, D., Memon, F.A., Jones, N., Galloway, T., 2022. Investigation and analysis of microplastics in sewage sludge and biosolids: A case study from one wastewater treatment works in the UK. Sci. Total Environ. 823, 153735.

https://doi.org/10.1016/j.scitotenv.2022.153735.

Harrad, S., Drage, D.S., Sharkey, M., Berresheim, H., 2020. Perfluoroalkyl substances and brominated flame retardants in landfill-related air, soil, and groundwater from Ireland. Sci. Total Environ. 705, 135834. https://doi.org/10.1016/j.scitotenv.2019.135834.

Heo, J., Yoon, Y., Lee, G., Kim, Y., Han, J., Park, C.M., 2019. Enhanced adsorption of bisphenol A and sulfamethoxazole by a novel magnetic CuZnFe2O4–biochar composite. Bioresour. Technol. 281, 179–187. https://doi.org/10.1016/j.biortech.2019.02.091.

Hernández-Fernández, J., Cano-Cuadro, H., Puello-Polo, E., 2022. Emission of bisphenol A and four new analogs from industrial wastewater treatment plants in the production processes of polypropylene and polyethylene terephthalate in South America. Sustainability 14, 2022. https://doi.org/10.3390/su141710919.

Hernandez, E.T., Koo, B., Sofen, L.E., Amin, R., Togashi, R.K., Lall, A.I., et al., 2022. Proteins as adsorbents for PFAS removal from water. Environ. Sci.: Water Res. Technol. 8, 1188–1194. https://doi.org/10.1039/D1EW00501D.

Hernández, F., Ibáñez, M., Portoles, T., Hidalgo-Troya, A., Ramírez, J.D., Paredes, M.A., et al., 2024. High-resolution mass spectrometry-based screening for the comprehensive investigation of organic micropollutants in surface water and wastewater from Pasto city, Colombian Andean highlands. Sci. Total Environ. 922, 171293. https://doi.org/10.1016/j.scitotenv.2024.171293.

Herzke, D., Ghaffari, P., Sundet, J.H., Tranang, C.A., Halsband, C., 2021. Microplastic fiber emissions from wastewater effluents: Abundance, transport behavior and exposure risk for biota in an Arctic fjord. Front. Environ. Sci. 9. https://doi.org/10.3389/fenvs.2021.662168.

Higgins, C., Turner, A., 2023. Microplastics in surface coastal waters around Plymouth, UK, and the

contribution of boating and shipping activities. Sci. Total Environ. 893, 164695. https://doi.org/10.1016/j.scitotenv.2023.164695.

Ho, K.T., Konovets, I.M., Terletskaya, A.V., Milyukin, M.V., Lyashenko, A.V., Shitikova, L.I., et al., 2020. Contaminants, mutagenicity and toxicity in the surface waters of Kyiv, Ukraine. Mar. Pollut. Bull. 155, 111153. https://doi.org/10.1016/j.marpolbul.2020.111153.

Hossain, M.B., Yu, J., Banik, P., Noman, M.A., Nur, A.-A.U., Haque, M.R., et al., 2023. First evidence of microplastics and their characterization in bottled drinking water from a developing country. Front. Environ. Sci. 11, 1232931. https://doi.org/10.3389/fenvs.2023.1232931.

Hossain, M.J., AftabUddin, S., Akhter, F., Nusrat, N., Rahaman, A., Sikder, M.N.A., et al., 2022. Surface water, sediment, and biota: The first multi-compartment analysis of microplastics in the Karnafully River, Bangladesh. Mar. Pollut. Bull. 180, 113820. https://doi.org/10.1016/j.marpolbul.2022.113820.

Hosseini, R., Sayadi, M.H., Aazami, J., Savabieasfehani, M., 2020. Accumulation and distribution of microplastics in the sediment and coastal water samples of Chabahar Bay in the Oman Sea, Iran. Mar. Pollut. Bull. 160, 111682. https://doi.org/10.1016/j.marpolbul.2020.111682.

Hrkal, Z., Adomat, Y., Rozman, D., Grischek, T., 2023. Efficiency of micropollutant removal through artificial recharge and riverbank filtration: Case studies of Káraný, Czech Republic, and Dresden-Hosterwitz, Germany. Environ. Earth Sci. 82, 155. https://doi.org/10.1007/s12665-023-10785-7.

Hron, L.M.C., Wöckner, M., Fuchs, V., Fembacher, L., Aschenbrenner, B., Herr, C., et al., 2024. Monitoring of per- and polyfluoroalkyl substances (PFAS) in human blood samples collected in three regions with known PFAS releases in the environment and three control regions in South

Germany. Arch. Toxicol. 98, 3727–3738. https://doi.org/10.1007/s00204-024-03843-x.

Hu, K., Zhou, P., Yang, Y., Hall, T., Nie, G., Yao, Y., et al., 2022. Degradation of microplastics by a thermal Fenton reaction. ACS ES&T Eng. 2, 110–120. https://doi.org/10.1021/acsestengg.1c00323.

Huang, D., Li, X., Ouyang, Z., Zhao, X., Wu, R., Zhang, C., et al., 2021. The occurrence and abundance of microplastics in surface water and sediment of the West River downstream, in the south of China. Sci. Total Environ. 756, 143857. https://doi.org/10.1016/j.scitotenv.2020.143857.

Huang, J., Wang, X., Pan, Z., Li, X., Ling, Y., Li, L., 2016. Efficient degradation of perfluorooctanoic acid (PFOA) by photocatalytic ozonation. Chem. Eng. J. 296, 329–334. https://doi.org/10.1016/j.cej.2016.03.116.

Huang, Y., Yu, L., Ma, L., Zhang, D., Xu, J., Zhang, S., et al., 2023. FeS combined ozonation to remove p-aminobenzenesulfonamide from water: Density functional theory insights into the mechanism. Chemosphere 311, 137158. https://doi.org/10.1016/j.chemosphere.2022.137158.

Hunter, R.G., Day, J.W., Wiegman, A.R., Lane, R.R., 2019. Municipal wastewater treatment costs with an emphasis on assimilation wetlands in the Louisiana coastal zone. Ecol. Eng. 137, 21–25. https://doi.org/10.1016/j.ecoleng.2018.09.020.

Islam, A.R.M.T., Hasan, M., Sadia, M.R., Mubin, A.-N., Ali, M.M., Senapathi, V., et al., 2024. Unveiling microplastics pollution in a subtropical rural recreational lake: A novel insight. Environ. Res. 250, 118543. https://doi.org/10.1016/j.envres.2024.118543.

Islam, M.S., Islam, Z., Hasan, M.R., 2022. Pervasiveness and characteristics of microplastics in surface water and sediment of the Buriganga River, Bangladesh. Chemosphere 307, 135945. https://doi.org/10.1016/j.chemosphere.2022.135945.

Islam, M.S., Islam, Z., Jamal, A.H.M.S.I.M., Momtaz, N., Beauty, S.A., 2023. Removal efficiencies of microplastics of the three largest drinking water treatment plants in Bangladesh. Sci. Total Environ. 895, 165155. https://doi.org/10.1016/j.scitotenv.2023.165155.

Ismanto, A., Hadibarata, T., Kristanti, R.A., Maslukah, L., Safinatunnajah, N., Sathishkumar, P., 2022. The abundance of endocrine-disrupting chemicals (EDCs) in downstream of the Bengawan Solo and Brantas rivers located in Indonesia. Chemosphere 297, 134151. https://doi.org/10.1016/j.chemosphere.2022.134151.

Jahan, I., Chowdhury, G., Baquero, A.O., Couetard, N., Hossain, M.A., Mian, S., et al., 2024. Microplastics pollution in the Surma River, Bangladesh: A rising hazard to upstream water quality and aquatic life. J. Environ. Manag. 360, 121117. https://doi.org/10.1016/j.jenvman.2024.121117.

Jara-Negrete, E., Cipriani-Avila, I., Molinero, J., Pinos-Vélez, V., Acosta-López, S., Cabrera, M., et al., 2023. Pharmaceutical compounds in urban drinking waters of Ecuador. Front. Environ. Sci. 11, 1232405. https://doi.org/10.3389/fenvs.2023.1232405.

Jiang, R., Liu, J., Huang, B., Wang, X., Luan, T., Yuan, K., 2020. Assessment of the potential ecological risk of residual endocrine-disrupting chemicals from wastewater treatment plants. Sci. Total Environ. 714, 136689. https://doi.org/10.1016/j.scitotenv.2020.136689.

Jian, M., Chen, X., Liu, S., Liu, Y., Liu, Y., Wang, Q., et al., 2024. Combined exposure with microplastics increases the toxic effects of PFOS and its alternative F53B in adult zebrafish. Sci. Total Environ. 920, 170948. https://doi.org/10.1016/j.scitotenv.2024.170948

Jin, Y., Yuan, T., Li, J., Shen, Z., Tian, Y., 2022. Occurrence, health risk assessment and water quality criteria derivation of six personal care products (PCPs) in Huangpu River, China. Environ.

Monit. Assess. 194, 577. https://doi.org/10.1007/s10661-022-10271-w.

Johnson, G.R., 2022. PFAS in soil and groundwater following historical land application of biosolids. Water Res. 211, 118035. https://doi.org/10.1016/j.watres.2021.118035.

Jurikova, M., Dvorakova, D., Pulkrabova, J., 2022. The occurrence of perfluoroalkyl substances (PFAS) in drinking water in the Czech Republic: A pilot study. Environ. Sci. Pollut. Res. 29, 60341–60353. https://doi.org/10.1007/s11356-022-20156-7.

K'Oreje, K., Okoth, M., Van Langenhove, H., Demeestere, K., 2022. Occurrence and point-of-use treatment of contaminants of emerging concern in groundwater of the Nzoia River basin, Kenya. Environ. Pollut. 297, 118725. https://doi.org/10.1016/j.envpol.2021.118725.

K, M.B., Natesan, U., R, V., R, P.K., R, R., S, S., 2021. Spatial distribution of microplastic concentration around landfill sites and its potential risk on groundwater. Chemosphere 277, 130263. https://doi.org/10.1016/j.chemosphere.2021.130263.

Kandie, F.J., Krauss, M., Beckers, L.-M., Massei, R., Fillinger, U., Becker, J., et al., 2020. Occurrence and risk assessment of organic micropollutants in freshwater systems within the Lake Victoria South Basin, Kenya. Sci. Total Environ. 714, 136748. https://doi.org/10.1016/j.scitotenv.2020.136748.

Karimi, K.J., Ngumba, E., Ahmad, A., Duse, A.G., Olago, D., Ndwigah, S.N., et al., 2023. Contamination of groundwater with sulfamethoxazole and antibiotic resistant *Escherichia coli* in informal settlements in Kisumu, Kenya. PLOS Water 2, e0000076. https://doi.org/10.1371/journal.pwat.0000076.

Kasula, M., Pala, J., Esfahani, M.R., 2024. Designing super fine activated carbon-functionalized thin-film nanocomposite membranes for adsorptive removal of per- and poly-fluoroalkyl substances.

ACS Appl. Eng. Mater. 2, 143–155. https://doi.org/10.1021/acsaenm.3c00670.

Khan, H.K., Rehman, M.Y.A., Junaid, M., Lv, M., Yue, L., Haq, I.-u., et al., 2022. Occurrence, source apportionment and potential risks of selected PPCPs in groundwater used as a source of drinking water from key urban-rural settings of Pakistan. Sci. Total Environ. 807, 151010. https://doi.org/10.1016/j.scitotenv.2021.151010.

Khan, S., Ali, S.A., Ali, A.S., 2023. Biodegradation of low density polyethylene (LDPE) by mesophilic fungus *Penicillium citrinum* isolated from soils of plastic waste dump yard, Bhopal, India. Environ. Technol. 44, 2300–2314. https://doi.org/10.1080/09593330.2022.2027025.

Khavar, A.H.C., Khedri, N., Rizo, R., Feliu Martínez, J.M., Mahjoub, A.R., Doolabi, M., et al., 2023. A novel Ga(III) coordination complex as an efficient sensitizer for enhancing photocatalytic activity of TiO2/rGO nanocomposite. J. Rare Earths 41, 1004–1013. https://doi.org/10.1016/j.jre.2022.05.015.

Kheireddine, O., Chanez, L., Rania, D., Fouzia, T., Faouzi, S.J.A., 2024. Evaluation of sediment contamination by macro and microplastics in coastal waters of Southern Mediterranean: A case study of Annaba, Algeria, before and after the COVID-19 pandemic. *Arch. Environ. Pollut.* 21, 31. https://doi.org/10.24425/aep.2024.150549.

Khezami, F., Gómez-Navarro, O., Barbieri, M.V., Khiari, N., Chkirbene, A., Chiron, S., et al., 2024. Occurrence of contaminants of emerging concern and pesticides and relative risk assessment in Tunisian groundwater. Sci. Total Environ. 906, 167319. https://doi.org/10.1016/j.scitotenv.2023.167319.

Khan, F.R., Patsiou, D., Catarino, A.I., 2022. Pollutants bioavailability and toxicological risk from microplastics. In: RochaSantos, T., Costa, M., Mouneyrac, C.(Eds.), Handbook of Microplastics

in the Environment. Springer International Publishing, Cham, pp. 122. https://doi.org/10.1007/978-3-030-39041-9_19

Kiendrebeogo, M., Karimi Estahbanati, M.R., Khosravanipour Mostafazadeh, A., Drogui, P., Tyagi, R.D., 2021. Treatment of microplastics in water by anodic oxidation: A case study for polystyrene. Environ. Pollut. 269, 116168. https://doi.org/10.1016/j.envpol.2020.116168.

Kılıç, E., Yücel, N., Bengil, F., Bengil, E.G.T., Şahutoğlu, S.M., 2024. Microplastic pollution levels in the surface water and sediment of Orontes basin: Urgent risk for endangered species. Mar. Pollut. Bull. 208, 116945. https://doi.org/10.1016/j.marpolbul.2024.116945.

Kleywegt, S., Raby, M., McGill, S., Helm, P., 2020. The impact of risk management measures on the concentrations of per- and polyfluoroalkyl substances in source and treated drinking waters in Ontario, Canada. Sci. Total Environ. 748, 141195. https://doi.org/10.1016/j.scitotenv.2020.141195.

Kodom, K., Attiogbe, F., Kuranchie, F.A., 2021. Assessment of removal efficiency of pharmaceutical products from wastewater in sewage treatment plants: A case of the Sewerage Systems Ghana Limited, Accra. Heliyon 7, e08385. https://doi.org/10.1016/j.heliyon.2021.e08385.

Kortesmäki, E., Östman, J.R., Meierjohann, A., Brozinski, J.M., Eklund, P., Kronberg, L., et al., 2020. Occurrence of antibiotics in influent and effluent from three major wastewater-treatment plants in Finland. Environ. Toxicol. Chem. 39, 1774–1789. https://doi.org/10.1002/etc.4805.

Kovalova, L., Siegrist, H., von Gunten, U., Eugster, J., Hagenbuch, M., Wittmer, A., et al., 2013. Elimination of micropollutants during post-treatment of hospital wastewater with powdered activated carbon, ozone, and UV. Environ. Sci. Technol. 47, 7899–7908. https://doi.org/10.1021/es400708w.

Kozisek, F., Dvorakova, D., Kotal, F., Jeligova, H., Mayerova, L., Svobodova, V., et al., 2025. Assessing PFAS in drinking water: Insights from the Czech Republic's risk-based monitoring approach. Chemosphere 370, 143969. https://doi.org/10.1016/j.chemosphere.2024.143969.

Lalonde, B., Garron, C., 2020. Spatial and temporal distribution of BPA in the Canadian freshwater environment. Arch. Environ. Contam. Toxicol. 78, 568–578. https://doi.org/10.1007/s00244-020-00721-2.

Larrea Valdivia, A.E., Larico, J.R., Valenzuela Huillca, C., Arias, A.H., 2025. First evidence of microplastics in the Quilca-Vítor-Chili river basin, Arequipa region, Peru. J. Contam. Hydrol. 269, 104484. https://doi.org/10.1016/j.jconhyd.2024.104484.

Lath, S., Navarro, D.A., Losic, D., Kumar, A., McLaughlin, M.J., 2018. Sorptive remediation of perfluorooctanoic acid (PFOA) using mixed mineral and graphene/carbon-based materials. Environ. Chem. 15, 472–480. https://doi.org/10.1071/EN18156.

Lechthaler, S., Waldschläger, K., Sandhani, C.G., Sannasiraj, S.A., Sundar, V., Schwarzbauer, J., et al., 2021. Baseline study on microplastics in Indian rivers under different anthropogenic influences. Water 13, 2021.

Lee, Y.-M., Lee, J.-Y., Kim, M.-K., Yang, H., Lee, J.-E., Son, Y., et al., 2020. Concentration and distribution of per- and polyfluoroalkyl substances (PFAS) in the Asan Lake area of South Korea. J. Hazard. Mater. 381, 120909. https://doi.org/10.1016/j.jhazmat.2019.120909.

Lefebvre, C., Le Bihanic, F., Jalón-Rojas, I., Dusacre, E., Chassaigne--Viscaïno, L., Bichon, J., et al., 2023. Spatial distribution of anthropogenic particles and microplastics in a meso-tidal lagoon (Arcachon Bay, France): A multi-compartment approach. Sci. Total Environ. 898, 165460. https://doi.org/10.1016/j.scitotenv.2023.165460.

Lei, K., Lin, C.-Y., Zhu, Y., Chen, W., Pan, H.-Y., Sun, Z., et al., 2020. Estrogens in municipal wastewater and receiving waters in the Beijing-Tianjin-Hebei region, China: Occurrence and risk assessment of mixtures. J. Hazard. Mater. 389, 121891. https://doi.org/10.1016/j.jhazmat.2019.121891.

Lestari, P., Trihadiningrum, Y., Wijaya, B.A., Yunus, K.A., Firdaus, M., 2020. Distribution of microplastics in Surabaya River, Indonesia. Sci. Total Environ. 726, 138560. https://doi.org/10.1016/j.scitotenv.2020.138560.

Leterme, S.C., Tuuri, E.M., Drummond, W.J., Jones, R., Gascooke, J.R., 2023. Microplastics in urban freshwater streams in Adelaide, Australia: A source of plastic pollution in the Gulf St Vincent. Sci. Total Environ. 856, 158672. https://doi.org/10.1016/j.scitotenv.2022.158672.

Li, G., Huang, Z., Li, H., Zhang, Z., Cui, L., 2023. Synergistic removal of sulfamethoxazole and dimethyl phthalate by five constructed wetland substrates. Chemosphere 318, 137936. https://doi.org/10.1016/j.chemosphere.2023.137936.

Li, J., Ouyang, Z., Liu, P., Zhao, X., Wu, R., Zhang, C., et al., 2021. Distribution and characteristics of microplastics in the basin of Chishui River in Renhuai, China. Sci. Total Environ. 773, 145591. https://doi.org/10.1016/j.scitotenv.2021.145591.

Li, Y., Li, J., Ding, J., Song, Z., Yang, B., Zhang, C., et al., 2022a. Degradation of nano-sized polystyrene plastics by ozonation or chlorination in drinking water disinfection processes. Chem. Eng. J. 427, 131690. https://doi.org/10.1016/j.cej.2021.131690.

Li, Y., Liu, S., Wang, C., Ying, Z., Huo, M., Yang, W., 2020. Effective column adsorption of triclosan from pure water and wastewater treatment plant effluent by using magnetic porous reduced graphene oxide. J. Hazard. Mater. 386, 121942. https://doi.org/10.1016/j.jhazmat.2019.121942.

Li, Y., Thompson, J., Wang, Z., Bräunig, J., Zheng, Q., Thai, P.K., et al., 2022b. Transformation and fate of pharmaceuticals, personal care products, and per- and polyfluoroalkyl substances during aerobic digestion of anaerobically digested sludge. Water Res. 219, 118568. https://doi.org/10.1016/j.watres.2022.118568.

Liang, Y., Song, H., Wu, Y., Gao, S., Zeng, X., Yu, Z., 2022. Occurrence and distribution of triclosan and its transformation products in Taihu Lake, China. Environ. Sci. Pollut. Res. 29, 84787–84797. https://doi.org/10.1007/s11356-022-21568-1.

Liddie, J.M., Bind, M.-A., Karra, M., Sunderland, E.M., 2024. County-level associations between drinking water PFAS contamination and COVID-19 mortality in the United States. J. Expo. Sci. Environ. Epidemiol. https://doi.org/10.1038/s41370-024-00723-5.

Lin, D., Cen, Z., Zhang, C., Lin, X., Liang, T., Xu, Y., et al., 2024. Triclosanloaded aged microplastics exacerbate oxidative stress and neurotoxicity in *Xenopus tropicalis* tadpoles via increased bioa ccumulation. Sci. Total Environ. 935, 173457. https://doi.org/10.1016/j.scitotenv.2024.173457

Lin, X., Xu, J., Keller, A.A., He, L., Gu, Y., Zheng, W., et al., 2020. Occurrence and risk assessment of emerging contaminants in a water reclamation and ecological reuse project. Sci. Total Environ. 744, 140977. https://doi.org/10.1016/j.scitotenv.2020.140977.

Liu, J., Zhao, Z., Li, J., Hua, X., Zhang, B., Tang, C., et al., 2023. Emerging and legacy perfluoroalkyl and polyfluoroalkyl substances (PFAS) in surface water around three international airports in China. Chemosphere 344, 140360. https://doi.org/10.1016/j.chemosphere.2023.140360.

Liu, M., Munoz, G., Vo Duy, S., Sauvé, S., Liu, J., 2022. Per- and polyfluoroalkyl substances in contaminated soil and groundwater at airports: A Canadian case study. Environ. Sci. Technol. 56, 885–895. https://doi.org/10.1021/acs.est.1c04798.

Liu, S., Wang, C., Wang, P., Chen, J., Wang, X., Yuan, Q., 2021. Anthropogenic disturbances on distribution and sources of pharmaceuticals and personal care products throughout the Jinsha River Basin, China. Environ. Res. 198, 110449. https://doi.org/10.1016/j.envres.2020.110449.

Liu, Y., Feng, M., Wang, B., Zhao, X., Guo, R., Bu, Y., et al., 2020. Distribution and potential risk assessment of antibiotic pollution in the main drinking water sources of Nanjing, China. Environ. Sci. Pollut. Res. 27, 21429–21441. https://doi.org/10.1007/s11356-020-08516-7.

Llamas-Dios, M.I., Vadillo, I., Jiménez-Gavilán, P., Candela, L., Corada-Fernández, C., 2021. Assessment of a wide array of contaminants of emerging concern in a Mediterranean water basin (Guadalhorce river, Spain): Motivations for an improvement of water management and pollutants surveillance. Sci. Total Environ. 788, 147822. https://doi.org/10.1016/j.scitotenv.2021.147822.

Lopez-Herguedas, N., Irazola, M., Alvarez-Mora, I., Orive, G., Lertxundi, U., Olivares, M., et al., 2023. Comprehensive micropollutant characterization of wastewater during COVID-19 crisis in 2020: Suspect screening and environmental risk prioritization strategy. Sci. Total Environ. 873, 162281. https://doi.org/10.1016/j.scitotenv.2023.162281.

López-Velázquez, K., Guzmán-Mar, J.L., Saldarriaga-Noreña, H.A., Murillo-Tovar, M.A., Hinojosa-Reyes, L., Villanueva-Rodríguez, M., 2021. Occurrence and seasonal distribution of five selected endocrine-disrupting compounds in wastewater treatment plants of the Metropolitan Area of Monterrey, Mexico: The role of water quality parameters. Environ. Pollut. 269, 116223. https://doi.org/10.1016/j.envpol.2020.116223.

López, C., Soto, L.M., Acosta, V., Santana-Piñeros, A.M., Cruz-Quintana, Y., Gomes-Barbosa, L., et al., 2023. A first step to assess suspended microplastics in a freshwater wetland from the coastal

region of Ecuador. Front. Environ. Sci. 11, 1028970. https://doi.org/10.3389/fenvs.2023.1028970.

Lopez, F.J., Pitarch, E., Botero-Coy, A.M., Fabregat-Safont, D., Ibáñez, M., Marin, J.M., et al., 2022. Removal efficiency for emerging contaminants in a WWTP from Madrid (Spain) after secondary and tertiary treatment and environmental impact on the Manzanares River. Sci. Total Environ. 812, 152567. https://doi.org/10.1016/j.scitotenv.2021.152567.

Lugo-Bueno, S.F., García-Morales, R., Coronel, R., Aguilar-Hernandez, I., Becerril-Bravo, J.E., Barrios-Perez, J.A., et al., 2022. Biocatalysis assisted by electrochemical processes for the removal of bisphenol A and triclosan in wastewater. Environ. Technol. Innov. 28, 102921. https://doi.org/10.1016/j.eti.2022.102921.

Luo, Y., Xie, H., Xu, H., Zhou, C., Wang, P., Liu, Z., et al., 2023. Wastewater treatment plant serves as a potentially controllable source of microplastic: Association of microplastic removal and operational parameters and water quality data. J. Hazard. Mater. 441, 129974. https://doi.org/10.1016/j.jhazmat.2022.129974.

Luo, Z., Liu, M., Tang, D., Xu, Y., Ran, H., He, J., et al., 2022. High H2O2 selectivity and enhanced Fe2+ regeneration toward an effective electro-Fenton process based on a self-doped porous biochar cathode. Appl. Catal. B Environ. 315, 121523. https://doi.org/10.1016/j.apcatb.2022.121523.

Madeira, C.L., Acayaba, R.D.A., Santos, V.S., Villa, J.E.L., Jacinto-Hernández, C., Azevedo, J.A.T., et al., 2023. Uncovering the impact of agricultural activities and urbanization on rivers from the Piracicaba, Capivari, and Jundiaí basin in São Paulo, Brazil: A survey of pesticides, hormones, pharmaceuticals, industrial chemicals, and PFAS. Chemosphere 341, 139954.

https://doi.org/10.1016/j.chemosphere.2023.139954.

Mahamuni, N.N., Adewuyi, Y.G., 2010. Advanced oxidation processes (AOPs) involving ultrasound for wastewater treatment: A review with emphasis on cost estimation. Ultrason. Sonochem. 17, 990–1003. https://doi.org/10.1016/j.ultsonch.2009.09.005.

Mainardis, M., Buttazzoni, M., De Bortoli, N., Mion, M., Goi, D., 2020. Evaluation of ozonation applicability to pulp and paper streams for a sustainable wastewater treatment. J. Clean. Prod. 258, 120781. https://doi.org/10.1016/j.jclepro.2020.120781.

Martínez-Alcalá, I., Guillén-Navarro, J.M., Lahora, A., 2021. Occurrence and fate of pharmaceuticals in a wastewater treatment plant from southeast of Spain and risk assessment. J. Environ. Manag. 279, 111565. https://doi.org/10.1016/j.jenvman.2020.111565.

Maryam, B., Buscio, V., Odabasi, S.U., Buyukgungor, H., 2020. A study on behavior, interaction and rejection of Paracetamol, Diclofenac and Ibuprofen (PhACs) from wastewater by nanofiltration membranes. Environ. Technol. Innov. 18, 100641. https://doi.org/10.1016/j.eti.2020.100641.

McKenzie, T., Holloway, C., Dulai, H., Tucker, J.P., Sugimoto, R., Nakajima, T., et al., 2020. Submarine groundwater discharge: A previously undocumented source of contaminants of emerging concern to the coastal ocean (Sydney, Australia). Mar. Pollut. Bull. 160, 111519. https://doi.org/10.1016/j.marpolbul.2020.111519.

McMahon, P.B., Tokranov, A.K., Bexfield, L.M., Lindsey, B.D., Johnson, T.D., Lombard, M.A., et al., 2022. Perfluoroalkyl and polyfluoroalkyl substances in groundwater used as a source of drinking water in the eastern United States. Environ. Sci. Technol. 56, 2279–2288. https://doi.org/10.1021/acs.est.1c04795.

Mercy, F.T., Alam, A.K.M.R., Akbor, M.A., 2023. Abundance and characteristics of microplastics in

major urban lakes of Dhaka, Bangladesh. Heliyon 9, e14587. https://doi.org/10.1016/j.heliyon.2023.e14587.

Mhlongo, S.A., Sibali, L.L., Ndibewu, P.P., 2023. Occurrence, quantification and removal of triclosan in wastewater of Umbogintwini Industrial Complex in KwaMakhutha, South Africa. *S. Afr. J. Sci.* 119. https://doi.org/10.17159/sajs.2023/14743.

Mhuka, V., Dube, S., Nindi, M.M., 2020. Occurrence of pharmaceutical and personal care products (PPCPs) in wastewater and receiving waters in South Africa using LC-Orbitrap™ MS. Emerg. Contam. 6, 250–258. https://doi.org/10.1016/j.emcon.2020.07.002.

Miao, F., Liu, Y., Gao, M., Yu, X., Xiao, P., Wang, M., et al., 2020. Degradation of polyvinyl chloride microplastics via an electro-Fenton-like system with a TiO2/graphite cathode. J. Hazard. Mater. 399, 123023. https://doi.org/10.1016/j.jhazmat.2020.123023.

Mo, L., Fu, H., Lu, Q., Chen, S., Liu, R., Xiang, J., et al., 2024. Characteristics and ecological risks of microplastic pollution in a tropical drinking water source reservoir in Hainan province, China. Environ. Sci.: Process. Impacts 26, 451–460. https://doi.org/10.1039/D3EM00528C.

Moazeni, M., Ebrahimpour, K., Mohammadi, F., Heidari, Z., Ebrahimi, A., 2023. Human health risk assessment of triclosan in water: Spatial analysis of a drinking water system. Environ. Monit. Assess. 195, 1171. https://doi.org/10.1007/s10661-023-11789-3.

Moid AlAmmari, A., Rizwan Khan, M., Aqel, A., 2020. Trace identification of endocrine-disrupting bisphenol A in drinking water by solid-phase extraction and ultra-performance liquid chromatography-tandem mass spectrometry. J. King Saud Univ. Sci. 32, 1634–1640. https://doi.org/10.1016/j.jksus.2019.12.022.

Montes, R., Méndez, S., Cobas, J., Carro, N., Neuparth, T., Alves, N., et al., 2023. Occurrence of

persistent and mobile chemicals and other contaminants of emerging concern in Spanish and Portuguese wastewater treatment plants, transnational river basins and coastal water. Sci. Total Environ. 885, 163737. https://doi.org/10.1016/j.scitotenv.2023.163737.

Moral Pajares, E., Gallego Valero, L., Román Sánchez, I.M., 2019. Cost of urban wastewater treatment and ecotaxes: Evidence from municipalities in southern Europe. Water 11, 2019. https://doi.org/10.3390/w11030423.

Morales-Arredondo, J.I., Armienta-Hernández, M.A., Lugo-Dorantes, A.E., Barrera-Arrazola, A.P., Flores-Ocampo, I.Z., Flores-Vargas, R., 2023. Fluoride presence in drinking water along the southeastern part of El Bajío Guanajuatense, Guanajuato, Mexico: Sources and health effects. Environ. Geochem. Health 45, 3715–3742. https://doi.org/10.1007/s10653-022-01426-2.

Morales-McDevitt, M.E., Dunn, M., Habib, A., Vojta, S., Becanova, J., Lohmann, R., 2022. Poly- and perfluorinated alkyl substances in air and water from Dhaka, Bangladesh. Environ. Toxicol. Chem. 41, 334–342. https://doi.org/10.1002/etc.5255.

Moreira, V.R., Lebron, Y.A.R., Santos, L.V.d.S., Amaral, M.C.S., 2021. Dead-end ultrafiltration as a cost-effective strategy for improving arsenic removal from high turbidity waters in conventional drinking water facilities. Chem. Eng. J. 417, 128132. https://doi.org/10.1016/j.cej.2020.128132.

Mostafa, A., Shaaban, H., Alqarni, A., Al-Ansari, R., Alrashidi, A., Al-Sultan, F., et al., 2023. Multi-class determination of pharmaceuticals as emerging contaminants in wastewater from Eastern Province, Saudi Arabia using eco-friendly SPE-UHPLC-MS/MS: Occurrence, removal and environmental risk assessment. Microchem. J. 187, 108453. https://doi.org/10.1016/j.microc.2023.108453.

Mu, H., Wang, Y., Zhang, H., Guo, F., Li, A., Zhang, S., et al., 2022. High abundance of microplastics in

groundwater in Jiaodong Peninsula, China. Sci. Total Environ. 839, 156318. https://doi.org/10.1016/j.scitotenv.2022.156318.

Mukhopadhyay, M., Sampath, S., Muñoz-Arnanz, J., Jiménez, B., Chakraborty, P., 2020. Plasticizers and bisphenol A in Adyar and Cooum riverine sediments, India: Occurrences, sources and risk assessment. Environ. Geochem. Health 42, 2789–2802. https://doi.org/10.1007/s10653-020-00516-3.

Müller, V., Kindness, A., Feldmann, J., 2023. Fluorine mass balance analysis of PFAS in communal waters at a wastewater plant from Austria. Water Res. 244, 120501. https://doi.org/10.1016/j.watres.2023.120501.

Munoz, G., Liu, M., Vo Duy, S., Liu, J., Sauvé, S., 2023. Target and nontarget screening of PFAS in drinking water for a large-scale survey of urban and rural communities in Québec, Canada. Water Res. 233, 119750. https://doi.org/10.1016/j.watres.2023.119750.

Mussabek, D., Söderman, A., Imura, T., Persson, K.M., Nakagawa, K., Ahrens, L., et al., 2023. PFAS in the drinking water source: Analysis of the contamination levels, origin and emission rates. Water 15. https://doi.org/10.3390/w15010137.

Naji, A., Azadkhah, S., Farahani, H., Uddin, S., Khan, F.R., 2021. Microplastics in wastewater outlets of Bandar Abbas city (Iran): A potential point source of microplastics into the Persian Gulf. Chemosphere 262, 128039. https://doi.org/10.1016/j.chemosphere.2020.128039.

Nan, B., Su, L., Kellar, C., Craig, N.J., Keough, M.J., Pettigrove, V., 2020. Identification of microplastics in surface water and Australian freshwater shrimp *Paratya australiensis* in Victoria, Australia. Environ. Pollut. 259, 113865. https://doi.org/10.1016/j.envpol.2019.113865.

Napper, I.E., Baroth, A., Barrett, A.C., Bhola, S., Chowdhury, G.W., Davies, B.F.R., et al., 2021. The

abundance and characteristics of microplastics in surface water in the transboundary Ganges River. Environ. Pollut. 274, 116348. https://doi.org/10.1016/j.envpol.2020.116348.

Nasri, E., de la Vega, A.C.S., Martí, C.B., Ben Mansour, H., Diaz-Cruz, M.S., 2024. Pharmaceuticals and personal care products in Tunisian hospital wastewater: Occurrence and environmental risk. Environ. Sci. Pollut. Res. 31, 2716–2731. https://doi.org/10.1007/s11356-023-31220-1.

Kumar, R., Sinha, R., Rakib, M. R. J., Padha, S., Ivy, N., Bhattacharya, S., Dhar, A. & Sharma, P. Microplastics pollution load in Sundarban delta of Bay of Bengal. *J. Hazard. Mater. Adv.* **2022**, 7, 100099.

Nayak, V., Cuhorka, J., Mikulášek, P., 2022. Separation of drugs by commercial nanofiltration membranes and their modelling. *Membranes* 12. https://doi.org/10.3390/membranes12050528

Nazifa, T.H., Kristanti, R.A., Ike, M., Kuroda, M., Hadibarata, T., 2020. Occurrence and distribution of estrogenic chemicals in river waters of Malaysia. Toxicol. Environ. Health Sci. 12, 65–74. https://doi.org/10.1007/s13530-020-00036-8.

Ngigi, A.N., Magu, M.M., Muendo, B.M., 2019. Occurrence of antibiotics residues in hospital wastewater, wastewater treatment plant, and in surface water in Nairobi County, Kenya. Environ. Monit. Assess. 192, 18. https://doi.org/10.1007/s10661-019-7952-8.

Ngumba, E., Gachanja, A., Nyirenda, J., Maldonado, J., Tuhkanen, T.J., 2020. Occurrence of antibiotics and antiretroviral drugs in source-separated urine, groundwater, surface water and wastewater in the peri-urban area of Chunga in Lusaka, Zambia. *Water Sci. Technol.* 46, 278–284. https://doi.org/10.17159/wsa/2020.v46.i2.8243.

Nguyen, H.T., McLachlan, M.S., Tscharke, B., Thai, P., Braeunig, J., Kaserzon, S., et al., 2022. Background release and potential point sources of per- and polyfluoroalkyl substances to

municipal wastewater treatment plants across Australia. Chemosphere 293, 133657. https://doi.org/10.1016/j.chemosphere.2022.133657.

Nguyen, H.T., Thai, P.K., Kaserzon, S.L., O'Brien, J.W., Mueller, J.F., 2024. Nationwide occurrence and discharge mass load of per- and polyfluoroalkyl substances in effluent and biosolids: A snapshot from 75 wastewater treatment plants across Australia. J. Hazard. Mater. 470, 134203. https://doi.org/10.1016/j.jhazmat.2024.134203.

Nickel, J.P., Sacher, F., Fuchs, S., 2021. Up-to-date monitoring data of wastewater and stormwater quality in Germany. Water Res. 202, 117452. https://doi.org/10.1016/j.watres.2021.117452.

Nieto-Juárez, J.I., Torres-Palma, R.A., Botero-Coy, A.M., Hernández, F., 2021. Pharmaceuticals and environmental risk assessment in municipal wastewater treatment plants and rivers from Peru. Environ. Int. 155, 106674. https://doi.org/10.1016/j.envint.2021.106674.

Nikolopoulou, V., Alygizakis, N.A., Nika, M.-C., Oswaldova, M., Oswald, P., Kostakis, M., et al., 2022. Screening of legacy and emerging substances in surface water, sediment, biota and groundwater samples collected in the Siverskyi Donets River Basin employing wide-scope target and suspect screening. Sci. Total Environ. 805, 150253. https://doi.org/10.1016/j.scitotenv.2021.150253.

Nousheen, R., Hashmi, I., Rittschof, D., Capper, A., 2022. Comprehensive analysis of spatial distribution of microplastics in Rawal Lake, Pakistan using trawl net and sieve sampling methods. Chemosphere 308, 136111. https://doi.org/10.1016/j.chemosphere.2022.136111.

Odora, A.T., Aysha, S., Sultan, M.B., Bhuiyan, M.A.R., 2024. Evaluating the sources of microplastic contamination and quantifying its abundance in the Balu River, Dhaka, Bangladesh. Environ. Monit. Assess. 196, 867. https://doi.org/10.1007/s10661-024-13051-w.

Ofrydopoulou, A., Nannou, C., Evgenidou, E., Christodoulou, A., Lambropoulou, D., 2022. Assessment

of a wide array of organic micropollutants of emerging concern in wastewater treatment plants in Greece: Occurrence, removals, mass loading and potential risks. Sci. Total Environ. 802, 149860. https://doi.org/10.1016/j.scitotenv.2021.149860.

Oharisi, O.-o.L., Ncube, S., Nyoni, H., Madikizela, M.L., Olowoyo, O.J., Maseko, B.R., 2023. Occurrence and prevalence of antibiotics in wastewater treatment plants and effluent receiving rivers in South Africa using UHPLC-MS determination. J. Environ. Manag. 345, 118621. https://doi.org/10.1016/j.jenvman.2023.118621.

Oke, S.A., 2024. Contaminant of emerging concerns in Modder River Catchment of Free State: Implication for environmental risk and water sources protection. Water 16.

Oliveira, T.M.A., Mansano, A.S., Holanda, C.A., Pinto, T.S., Reis, J.B., Azevedo, E.B., et al., 2024. Occurrence and environmental risk assessment of contaminants of emerging concern in Brazilian surface waters. Environ. Toxicol. Chem. 43, 2199–2210. https://doi.org/10.1002/etc.5953.

Oni, B.A., Sanni, S.E., 2022. Occurrence of microplastics in borehole drinking water and sediments in Lagos, Nigeria. Environ. Toxicol. Chem. 41, 1721–1731. https://doi.org/10.1002/etc.5350.

Onipe, T., Edokpayi, J.N., Odiyo, J.O., 2021. Geochemical characterization and assessment of fluoride sources in groundwater of Siloam area, Limpopo Province, South Africa. Sci. Rep. 11, 14000. https://doi.org/10.1038/s41598-021-93385-4.

Ormaniec, P., 2024. Occurrence and analysis of microplastics in municipal wastewater, Poland. Environ. Sci. Pollut. Res. 31, 49646–49655. https://doi.org/10.1007/s11356-024-34488-z.

Paige, T., De Silva, T., Buddhadasa, S., Prasad, S., Nugegoda, D., Pettigrove, V., 2024. Background concentrations and spatial distribution of PFAS in surface waters and sediments of the greater

Melbourne area, Australia. Chemosphere 349, 140791. https://doi.org/10.1016/j.chemosphere.2023.140791.

Paray, B.A., Yu, J., Sultana, S., Banik, P., Nur, A.-A.U., Haque, M.R., et al., 2024. Contamination, morphological and chemical characterization, and hazard risk analyses of microplastics in drinking water sourced from groundwater in a developing nation. Front. Environ. Sci. 12, 1379311. https://doi.org/10.3389/fenvs.2024.1379311.

Park, H.-J., Oh, M.-J., Kim, P.-G., Kim, G., Jeong, D.-H., Ju, B.-K., et al., 2020. National reconnaissance survey of microplastics in municipal wastewater treatment plants in Korea. Environ. Sci. Technol. 54, 1503–1512. https://doi.org/10.1021/acs.est.9b04929.

Parto, M., Aazami, J., Shamsi, Z., Zamani, A., Savabieasfahani, M., 2022. Determination of bisphenol-A in plastic bottled water in markets of Zanjan, Iran. Int. J. Environ. Sci. Technol. 19, 3337–3344. https://doi.org/10.1007/s13762-021-03488-8.

Parvin, F., Hassan, M.A., Tareq, S.M., 2022. Risk assessment of microplastic pollution in urban lakes and peripheral rivers of Dhaka, Bangladesh. J. Hazard. Mater. Adv. 8, 100187. https://doi.org/10.1016/j.hazadv.2022.100187.

Pei, S., Li, B., Wang, B., Liu, J., Song, X., 2022. Distribution and ecological risk assessment of pharmaceuticals and personal care products in sediments of North Canal, China. Water 14. https://doi.org/10.3390/w14131999.

Pemberthy M, D., Padilla, Y., Echeverri, A., Peñuela, G.A., 2020. Monitoring pharmaceuticals and personal care products in water and fish from the Gulf of Urabá, Colombia. Heliyon 6. https://doi.org/10.1016/j.heliyon.2020.e04215.

Perraki, M., Skliros, V., Mecaj, P., Vasileiou, E., Salmas, C., Papanikolaou, I., et al., 2024. Identification

of microplastics using μ-Raman spectroscopy in surface and groundwater bodies of SE Attica, Greece. Water 16. https://doi.org/10.3390/w16060843.

Pétré, M.-A., Genereux, D.P., Koropeckyj-Cox, L., Knappe, D.R.U., Duboscq, S., Gilmore, T.E., et al., 2021. Per- and polyfluoroalkyl substance (PFAS) transport from groundwater to streams near a PFAS manufacturing facility in North Carolina, USA. Environ. Sci. Technol. 55, 5848–5856. https://doi.org/10.1021/acs.est.0c07978.

Picó, Y., Alvarez-Ruiz, R., Alfarhan, A.H., El-Sheikh, M.A., Alshahrani, H.O., Barceló, D., 2020. Pharmaceuticals, pesticides, personal care products and microplastics contamination assessment of Al-Hassa irrigation network (Saudi Arabia) and its shallow lakes. Sci. Total Environ. 701, 135021. https://doi.org/10.1016/j.scitotenv.2019.135021.

Picó, Y., Soursou, V., Alfarhan, A.H., El-Sheikh, M.A., Barceló, D., 2021. First evidence of microplastics occurrence in mixed surface and treated wastewater from two major Saudi Arabian cities and assessment of their ecological risk. J. Hazard. Mater. 416, 125747. https://doi.org/10.1016/j.jhazmat.2021.125747.

Pompei, C.M.E., Campos, L.C., da Silva, B.F., Fogo, J.C., Vieira, E.M., 2019. Occurrence of PPCPs in a Brazilian water reservoir and their removal efficiency by ecological filtration. Chemosphere 226, 210–219. https://doi.org/10.1016/j.chemosphere.2019.03.122.

Porras-Rojas, M.A., Charry-Vargas, C., Muñoz-Yustres, J.L., Martínez-Silva, P., Gómez-Méndez, L.D., 2023. Characterization of microplastics and mesoplastics and presence of biofilms, collected in the Gualí Wetland Cundinamarca, Colombia. Microplastics 2, 255–267. https://doi.org/10.3390/microplastics2030021.

Prajapati, S., Beal, M., Maley, J., Brinkmann, M., 2021. Qualitative and quantitative analysis of

microplastics and microfiber contamination in effluents of the City of Saskatoon wastewater treatment plant. Environ. Sci. Pollut. Res. 28, 32545–32553. https://doi.org/10.1007/s11356-021-12898-7.

Praveena, S.M., Shamsul Ariffin, N.I., Nafisyah, A.L., 2022. Microplastics in Malaysian bottled water brands: Occurrence and potential human exposure. Environ. Pollut. 315, 120494. https://doi.org/10.1016/j.envpol.2022.120494.

Pugazhenthiran, N., Murugesan, S., Valdés, H., Selvaraj, M., Sathishkumar, P., Smirniotis, P.G., et al., 2022. Photocatalytic oxidation of ceftiofur sodium under UV–visible irradiation using plasmonic porous Ag-TiO2 nanospheres. J. Ind. Eng. Chem. 105, 384–392. https://doi.org/10.1016/j.jiec.2021.09.038.

Qiao, B., Chen, H., Song, D., Yu, H., Baqar, M., Li, X., et al., 2024. Multimedia distribution and release characteristics of emerging PFAS in wastewater treatment plants in Tianjin, China. J. Hazard. Mater. 475, 134879. https://doi.org/10.1016/j.jhazmat.2024.134879.

Qiao, X., Li, X., Qi, T., Liu, Y., 2023. Identification of priority pollutants in groundwater: A case study in Xiong'an New Region, China. Water 15. https://doi.org/10.3390/w15081565.

Qin, L.-T., Pang, X.-R., Zeng, H.-H., Liang, Y.-P., Mo, L.-Y., Wang, D.-Q., et al., 2020. Ecological and human health risk of sulfonamides in surface water and groundwater of Huixian karst wetland in Guilin, China. Sci. Total Environ. 708, 134552. https://doi.org/10.1016/j.scitotenv.2019.134552.

Quyen, D.T., Masahiro, O., Otaki, Y., Chaminda, T., 2021. Sewage markers as determinants to differentiate origins of emerging organic pollutants in an urban Sri Lankan water drainage network. Water 13. https://doi.org/10.3390/w13202898.

Radwan, E.K., Ibrahim, M.B.M., Adel, A., Farouk, M., 2020. The occurrence and risk assessment of phenolic endocrine-disrupting chemicals in Egypt's drinking and source water. Environ. Sci. Pollut. Res. 27, 1776–1788. https://doi.org/10.1007/s11356-019-06887-0.

Rapp-Wright, H., Regan, F., White, B., Barron, L.P., 2023a. A year-long study of the occurrence and risk of over 140 contaminants of emerging concern in wastewater influent, effluent and receiving waters in the Republic of Ireland. Sci. Total Environ. 860, 160379. https://doi.org/10.1016/j.scitotenv.2022.160379.

Rapp-Wright, H., Rodríguez-Mozaz, S., Álvarez-Muñoz, D., Barceló, D., Regan, F., Barron, L.P., et al., 2023b. International comparison, risk assessment, and prioritisation of 26 endocrine disrupting compounds in three European river catchments in the UK, Ireland, and Spain. Molecules 28. https://doi.org/10.3390/molecules28165994.

Rauert, C., Vardy, S., Daniell, B., Charlton, N., Thomas, K.V., 2022. Tyre additive chemicals, tyre road wear particles and high production polymers in surface water at 5 urban centres in Queensland, Australia. Sci. Total Environ. 852, 158468. https://doi.org/10.1016/j.scitotenv.2022.158468.

Reichert, G., Mizukawa, A., Antonelli, J., de Almeida Brehm Goulart, F., Filippe, T.C., Rodrigues de Azevedo, J.C., 2020. Determination of parabens, triclosan, and lipid regulators in a subtropical urban river: Effects of urban occupation. Water Air Soil Pollut. 231, 133. https://doi.org/10.1007/s11270-020-04508-y.

Reinikainen, J., Perkola, N., Äystö, L., Sorvari, J., 2022. The occurrence, distribution, and risks of PFAS at AFFF-impacted sites in Finland. Sci. Total Environ. 829, 154237. https://doi.org/10.1016/j.scitotenv.2022.154237.

Riaz, R., Junaid, M., Rehman, M.Y.A., Iqbal, T., Khan, J.A., Dong, Y., et al., 2023. Spatial distribution,

compositional profile, sources, ecological and human health risks of legacy and emerging per- and polyfluoroalkyl substances (PFASs) in freshwater reservoirs of Punjab, Pakistan. Sci. Total Environ. 856, 159144. https://doi.org/10.1016/j.scitotenv.2022.159144.

Ridall, A., Farrar, E., Dansby, M., Ingels, J., 2023. Influence of wastewater treatment plants and water input sources on size, shape, and polymer distributions of microplastics in St. Andrew Bay, Florida, USA. Mar. Pollut. Bull. 187, 114552. https://doi.org/10.1016/j.marpolbul.2022.114552.

Riya, K.K., Anisuzzaman, M., Samad Azad, M.A., Ujjaman Nur, A.-A., Banik, P., Paray, B.A., et al., 2024. Characteristics, contamination levels, and ecosystem risk assessment of microplastics in surface water of a highly urbanized river from a developing country. ACS Omega 9, 50922–50932. https://doi.org/10.1021/acsomega.4c01528.

Rodrigues, J.A., Silva, S., Cardoso, V.V., Benoliel, M.J., Cardoso, E., Coelho, M.R., et al., 2021. Screening and seasonal behavior of analgesics, non-steroidal anti-inflammatory drugs, and antibiotics in two urban wastewater treatment plants. Environ. Manag. 68, 411–425. https://doi.org/10.1007/s00267-021-01496-5.

Rodriguez-Mozaz, S., Vaz-Moreira, I., Varela Della Giustina, S., Llorca, M., Barceló, D., Schubert, S., et al., 2020. Antibiotic residues in final effluents of European wastewater treatment plants and their impact on the aquatic environment. Environ. Int. 140, 105733. https://doi.org/10.1016/j.envint.2020.105733.

Rodríguez-Rodríguez, C.E., Ramírez-Morales, D., Masis-Mora, M., Montiel-Mora, J.R., Soto-Garita, C., Araya-Valverde, E., et al., 2023. Occurrence and risk assessment of pharmaceuticals in hospital wastewater in Costa Rica. Chemosphere 339, 139746. https://doi.org/10.1016/j.chemosphere.2023.139746.

Rodríguez-Varela, M., Durán-Álvarez, J.C., Jiménez-Cisneros, B., Zamora, O., Prado, B., 2021. Occurrence of perfluorinated carboxylic acids in Mexico City's wastewater: A monitoring study in the sewerage and a mega wastewater treatment plant. Sci. Total Environ. 774, 145060. https://doi.org/10.1016/j.scitotenv.2021.145060.

Rojas-Luna, R.A., Oquendo-Ruiz, L., García-Alzate, C.A., Arana, V.A., García-Alzate, R., Trilleras, J., 2023. Identification, abundance, and distribution of microplastics in surface water collected from Luruaco Lake, low basin Magdalena River, Colombia. *Water* 15. https://doi.org/10.3390/w15020344.

Romero-Murillo, P., Gallego, J.L., Leignel, V., 2023. Marine pollution and advances in biomonitoring in Cartagena Bay in the Colombian Caribbean. *Toxics* 11. https://doi.org/10.3390/toxics11070631.

Royano, S., de la Torre, A., Navarro, I., Martínez, M.Á., 2023. Pharmaceutically active compounds (PhACs) in surface water: Occurrence, trends and risk assessment in the Tagus River Basin (Spain). Sci. Total Environ. 905, 167422. https://doi.org/10.1016/j.scitotenv.2023.167422.

Rusiniak, P., Kmiecik, E., Wątor, K., Duda, R., Bugno, R., 2021. Pharmaceuticals and personal care products in the urban groundwater – preliminary monitoring (case study: Kraków, Southern Poland). Urban Water J. 18, 364–374. https://doi.org/10.1080/1573062X.2021.1893354.

Rusinque-Quintero, L.L., Montoya-Rojas, G.A., Moyano-Molano, A.L., 2022. Environmental risks due to the presence of microplastics in coastal and marine environments of the Colombian Caribbean. Mar. Pollut. Bull. 185, 114357. https://doi.org/10.1016/j.marpolbul.2022.114357.

Sá, B., Pais, J., Antunes, J., Pequeno, J., Pires, A., Sobral, P., 2022. Seasonal abundance and distribution patterns of microplastics in the Lis River, Portugal. Sustainability 14.

Saad, D., Ramaremisa, G., Ndlovu, M., Chauke, P., Nikiema, J., Chimuka, L., 2024. Microplastic

abundance and sources in surface water samples of the Vaal River, South Africa. Bull. Environ. Contam. Toxicol. 112, 23. https://doi.org/10.1007/s00128-023-03845-y.

Sabino, J.A., de Sá Salomão, A.L., de Oliveira Muniz Cunha, P.M., Coutinho, R., Marques, M., 2021. Occurrence of organic micropollutants in an urbanized sub-basin and ecological risk assessment. Ecotoxicology 30, 130–141. https://doi.org/10.1007/s10646-020-02304-2.

Sabri, N.A., Schmitt, H., Van der Zaan, B., Gerritsen, H.W., Zuidema, T., Rijnaarts, H.H.M., et al., 2020. Prevalence of antibiotics and antibiotic resistance genes in a wastewater effluent-receiving river in the Netherlands. J. Environ. Chem. Eng. 8, 102245. https://doi.org/10.1016/j.jece.2018.03.004.

Sadia, M., Nollen, I., Helmus, R., ter Laak, T.L., Béen, F., Praetorius, A., et al., 2023. Occurrence, fate, and related health risks of PFAS in raw and produced drinking water. Environ. Sci. Technol. 57, 3062–3074. https://doi.org/10.1021/acs.est.2c06015.

Sahar, E., David, I., Gelman, Y., Chikurel, H., Aharoni, A., Messalem, R., et al., 2011. The use of RO to remove emerging micropollutants following CAS/UF or MBR treatment of municipal wastewater. Desalination 273, 142–147. https://doi.org/10.1016/j.desal.2010.11.004.

Salma, U., Nishimura, Y., Tokumura, M., Hossain, A., Watanabe, K., Noro, K., et al., 2025. Occurrence, seasonal variation, and environmental risk of multiclass antibiotics in the urban surface water of the Buriganga River, Bangladesh. Chemosphere 370, 143956. https://doi.org/10.1016/j.chemosphere.2024.143956.

Samandra, S., Johnston, J.M., Jaeger, J.E., Symons, B., Xie, S., Currell, M., et al., 2022a. Microplastic contamination of an unconfined groundwater aquifer in Victoria, Australia. Sci. Total Environ. 802, 149727. https://doi.org/10.1016/j.scitotenv.2021.149727.

Samandra, S., Mescall, O.J., Plaisted, K., Symons, B., Xie, S., Ellis, A.V., et al., 2022b. Assessing exposure of the Australian population to microplastics through bottled water consumption. Sci. Total Environ. 837, 155329. https://doi.org/10.1016/j.scitotenv.2022.155329.

Samrat Hossain, M., Saifullah, A.S.M., Uddin, M.J., Hasibur Rahaman, M., 2024. Assessment of microplastics in coastal ecosystem of Bangladesh. Ecotoxicol. Environ. Saf. 281, 116622. https://doi.org/10.1016/j.ecoenv.2024.116622.

Sánchez-Campos, M., Ponce-Vélez, G., Sanvicente-Añorve, L., Alatorre-Mendieta, M., 2024. Microplastic contamination in three environmental compartments of a coastal lagoon in the southern Gulf of Mexico. Environ. Monit. Assess. 196, 1012. https://doi.org/10.1007/s10661-024-13156-2.

Santos, A.V., Couto, C.F., Lebron, Y.A.R., Moreira, V.R., Foureaux, A.F.S., Reis, E.O., et al., 2020. Occurrence and risk assessment of pharmaceutically active compounds in water supply systems in Brazil. Sci. Total Environ. 746, 141011. https://doi.org/10.1016/j.scitotenv.2020.141011.

Santos, V.S., Anjos, J.S.X., de Medeiros, J.F., Montagner, C.C., 2022. Impact of agricultural runoff and domestic sewage discharge on the spatial–temporal occurrence of emerging contaminants in an urban stream in São Paulo, Brazil. Environ. Monit. Assess. 194, 637. https://doi.org/10.1007/s10661-022-10288-1.

Šauer, P., Švecová, H., Grabicová, K., Gönül Aydın, F., Mackuľak, T., Kodeš, V., et al., 2021. Bisphenols emerging in Norwegian and Czech aquatic environments show transthyretin binding potency and other less-studied endocrine-disrupting activities. Sci. Total Environ. 751, 141801. https://doi.org/10.1016/j.scitotenv.2020.141801.

Säve-Söderbergh, M., Gyllenhammar, I., Schillemans, T., Lindfeldt, E., Vogs, C., Donat-Vargas, C., et

al., 2024. Per- and polyfluoroalkyl substances (PFAS) and fetal growth: A nation-wide register-based study on PFAS in drinking water. Environ. Int. 187, 108727. https://doi.org/10.1016/j.envint.2024.108727.

Saxena, P., Hiwrale, I., Das, S., Shukla, V., Tyagi, L., Pal, S., et al., 2021. Profiling of emerging contaminants and antibiotic resistance in sewage treatment plants: An Indian perspective. J. Hazard. Mater. 408, 124877. https://doi.org/10.1016/j.jhazmat.2020.124877.

Sayed, A.E.-D.H., Hamed, M., Badrey, A.E.A., Ismail, R.F., Osman, Y.A.A., Osman, A.G.M., et al., 2021. Microplastic distribution, abundance, and composition in the sediments, water, and fishes of the Red and Mediterranean seas, Egypt. Mar. Pollut. Bull. 173, 112966. https://doi.org/10.1016/j.marpolbul.2021.112966.

Schilling Costello, M.C., Asad, N., Haris, M., Yousefi, P., Khan, B., Lee, L.S., 2023. Reconnaissance survey of organic contaminants of emerging concern in the Kabul and Swat Rivers of Pakistan. Environ. Toxicol. Chem. 42, 2599–2613. https://doi.org/10.1002/etc.5750.

Schwartz, H., Marushka, L., Chan, H.M., Batal, M., Sadik, T., Ing, A., et al., 2021. Pharmaceuticals in source waters of 95 First Nations in Canada. Can. J. Public Health 112, 133–153. https://doi.org/10.17269/s41997-021-00499-3.

Schwichtenberg, T., Bogdan, D., Carignan, C.C., Reardon, P., Rewerts, J., Wanzek, T., et al., 2020. PFAS and dissolved organic carbon enrichment in surface water foams on a northern U.S. freshwater lake. Environ. Sci. Technol. 54, 14455–14464. https://doi.org/10.1021/acs.est.0c05697.

Segura, Y., Cruz del Álamo, A., Munoz, M., Álvarez-Torrellas, S., García, J., Casas, J.A., et al., 2021. A comparative study among catalytic wet air oxidation, Fenton, and Photo-Fenton technologies for the on-site treatment of hospital wastewater. J. Environ. Manag. 290, 112624.

https://doi.org/10.1016/j.jenvman.2021.112624.

Sekudewicz, I., Dąbrowska, A.M., Syczewski, M.D., 2021. Microplastic pollution in surface water and sediments in the urban section of the Vistula River (Poland). Sci. Total Environ. 762, 143111. https://doi.org/10.1016/j.scitotenv.2020.143111.

Selvam, S., Jesuraja, K., Venkatramanan, S., Roy, P.D., Jeyanthi Kumari, V., 2021. Hazardous microplastic characteristics and its role as a vector of heavy metal in groundwater and surface water of coastal south India. J. Hazard. Mater. 402, 123786. https://doi.org/10.1016/j.jhazmat.2020.123786.

Semerjian, L., Aissaoui, S., Shanableh, A., Okoh, A., Elhadi, R., Mousa, M., et al., 2023. Occurrence, spatial and seasonal variations of emerging contaminants in the aquatic environment of Sharjah, United Arab Emirates. Chemosphere 345, 140426. https://doi.org/10.1016/j.chemosphere.2023.140426.

Serna-Galvis, E.A., Botero-Coy, A.M., Rosero-Moreano, M., Lee, J., Hernández, F., Torres-Palma, R.A., 2022. An initial approach to the presence of pharmaceuticals in wastewater from hospitals in Colombia and their environmental risk. Water 14. https://doi.org/10.3390/w14060950.

Setiti, S., Hamdi, B., Chernai, S., Houma Bachari, F., Bachouche, S., Ghezali, Y., et al., 2021. Seasonal variation of microplastics density in Algerian surface waters (South-Western Mediterranean Sea). Mediterr. Mar. Sci. 22, 317–326. https://doi.org/10.12681/mms.24899.

Shafi, M., Jan, R., Gani, K.M., 2023. Selection of priority emerging contaminants in surface waters of India, Pakistan, Bangladesh, and Sri Lanka. Chemosphere 341, 139976. https://doi.org/10.1016/j.chemosphere.2023.139976.

Sharma, A., Jorvekar, S.B., Bhowmik, S., Mohapatra, P., Borkar, R.M., 2024. Comprehensive assessment

of per and polyfluoroalkyl substances (PFAS) contamination in groundwater of Kamrup, Assam, India: Occurrence, health risks, and metabolomic insights. Environ. Sci. Process. Impacts 26, 1601–1617. https://doi.org/10.1039/D4EM00159A.

Sharma, L., Siedlewicz, G., Pazdro, K., 2021. The toxic effects of antibiotics on freshwater and marine photosynthetic microorganisms: State of the art. Plants 10, 591. https://doi.org/10.3390/plants10030591

Shehab, Z.N., Jamil, N.R., Aris, A.Z., 2020. Occurrence, environmental implications and risk assessment of Bisphenol A in association with colloidal particles in an urban tropical river in Malaysia. Sci. Rep. 10, 20360. https://doi.org/10.1038/s41598-020-77454-8.

Shrestha, P., Jie, N., Wong, T.Y., 2020. Synergistic and antagonistic interactions of triclosan with various antibiotics in bacteria. J. Environ. Sci. Health Part C 38, 187-203. https://doi.org/10.1080/26896583.2020.1781494

Shi, J., Dong, Y., Shi, Y., Yin, T., He, W., An, T., et al., 2022. Groundwater antibiotics and microplastics in a drinking-water source area, northern China: Occurrence, spatial distribution, risk assessment, and correlation. Environ. Res. 210, 112855. https://doi.org/10.1016/j.envres.2022.112855.

Shu, X., Xu, L., Yang, M., Qin, Z., Zhang, Q., Zhang, L., 2023. Spatial distribution characteristics and migration of microplastics in surface water, groundwater and sediment in karst areas: The case of Yulong River in Guilin, Southwest China. Sci. Total Environ. 868, 161578. https://doi.org/10.1016/j.scitotenv.2023.161578.

Silva, S., Cardoso, V.V., Duarte, L., Carneiro, R.N., Almeida, C.M., 2021. Characterization of five Portuguese wastewater treatment plants: Removal efficiency of pharmaceutical active

compounds through conventional treatment processes and environmental risk. Appl. Sci. 11. https://doi.org/10.3390/app11167388.

Silver, M., Phelps, W., Masarik, K., Burke, K., Zhang, C., Schwartz, A., et al., 2023. Prevalence and source tracing of PFAS in shallow groundwater used for drinking water in Wisconsin, USA. Environ. Sci. Technol. 57, 17415–17426. https://doi.org/10.1021/acs.est.3c02826.

Sinkway, T.D., Mehdi, Q., Griffin, E.K., Correia, K., Camacho, C.G., Aufmuth, J., et al., 2024. Crowdsourcing citizens for statewide mapping of per- and polyfluoroalkyl substances (PFAS) in Florida drinking water. Sci. Total Environ. 926, 171932. https://doi.org/10.1016/j.scitotenv.2024.171932.

Ślósarczyk, K., Witkowski, A.J., 2024. Screening of pharmaceuticals and personal care products in the water environment of a region diversified in land use and urban development (Silesian Province, southern Poland). J. Hydrol. 635, 131191. https://doi.org/10.1016/j.jhydrol.2024.131191.

Snigirova, A.O., Mihas, R.V., Khutornoi, S.O., Vinogradov, A.K., Gazyetov, Y.I., Gascooke, J.R., et al., 2024. Microplastic and ichthyoplankton in the Ukrainian waters of the Black Sea. Reg. Stud. Mar. Sci. 80, 103884. https://doi.org/10.1016/j.rsma.2024.103884.

Sol, D., Menéndez-Manjón, A., Arias-García, P., Laca, A., Laca, A., Rancaño, A., et al., 2022. Occurrence of selected emerging contaminants in Southern Europe WWTPs: Comparison of simulations and real data. Processes 10. https://doi.org/10.3390/pr10122491.

Solaun, O., Rodríguez, J.G., Borja, Á., López-García, E., Zonja, B., Postigo, C., et al., 2022. Antibiotics in the Basque coast (N Spain): Occurrence in waste and receiving waters, and risk assessment (2017–2020). Sci. Total Environ. 847, 157563. https://doi.org/10.1016/j.scitotenv.2022.157563.

Sönmez, V.Z., Akarsu, C., Sivri, N., 2023. Impact of coastal wastewater treatment plants on microplastic

pollution in surface seawater and ecological risk assessment. Environ. Pollut. 318, 120922. https://doi.org/10.1016/j.envpol.2022.120922.

Sörengård, M., Bergström, S., McCleaf, P., Wiberg, K., Ahrens, L., 2022. Long-distance transport of per- and polyfluoroalkyl substances (PFAS) in a Swedish drinking water aquifer. Environ. Pollut. 311, 119981. https://doi.org/10.1016/j.envpol.2022.119981.

Sousa, J.C.G., Barbosa, M.O., Ribeiro, A.R.L., Ratola, N., Pereira, M.F.R., Silva, A.M.T., 2020. Distribution of micropollutants in estuarine and sea water along the Portuguese coast. Mar. Pollut. Bull. 154, 111120. https://doi.org/10.1016/j.marpolbul.2020.111120.

Stovall, J.K., Bratton, S.P., 2022. Microplastic pollution in surface waters of urban watersheds in Central Texas, United States: A comparison of sites with and without treated wastewater effluent. Front. Res. Anthropocene 2. https://doi.org/10.3389/frans.2022.857694.

Stroski, K.M., Luong, K.H., Challis, J.K., Chaves-Barquero, L.G., Hanson, M.L., Wong, C.S., 2020. Wastewater sources of per- and polyfluorinated alkyl substances (PFAS) and pharmaceuticals in four Canadian Arctic communities. Sci. Total Environ. 708, 134494. https://doi.org/10.1016/j.scitotenv.2019.134494.

Styszko, K., Proctor, K., Castrignanò, E., Kasprzyk-Hordern, B., 2021. Occurrence of pharmaceutical residues, personal care products, lifestyle chemicals, illicit drugs and metabolites in wastewater and receiving surface waters of Krakow agglomeration in South Poland. Sci. Total Environ. 768, 144360. https://doi.org/10.1016/j.scitotenv.2020.144360.

Sui, Q., Huang, J., Deng, S., Yu, G., Fan, Q., 2010. Occurrence and removal of pharmaceuticals, caffeine and DEET in wastewater treatment plants of Beijing, China. Water Res. 44, 417–426. https://doi.org/10.1016/j.watres.2009.07.010.

Sulistyowati, L., Nurhasanah, Riani, E., Cordova, M.R., 2022. The occurrence and abundance of microplastics in surface water of the midstream and downstream of the Cisadane River, Indonesia. Chemosphere 291, 133071. https://doi.org/10.1016/j.chemosphere.2021.133071.

Sun, M., Zhou, H., Xu, B., Bao, J., 2018. Distribution of perfluorinated compounds in drinking water treatment plant and reductive degradation by UV/SO32− process. Environ. Sci. Pollut. Res. 25, 7443–7453. https://doi.org/10.1007/s11356-017-1024-9.

Suteja, Y., Atmadipoera, A.S., Riani, E., Nurjaya, I.W., Nugroho, D., Cordova, M.R., 2021. Spatial and temporal distribution of microplastic in surface water of tropical estuary: Case study in Benoa Bay, Bali, Indonesia. Mar. Pollut. Bull. 163, 111979. https://doi.org/10.1016/j.marpolbul.2021.111979.

Szabo, D., Marchiandi, J., Samandra, S., Johnston, J.M., Mulder, R.A., Green, M.P., et al., 2023. High-resolution temporal wastewater treatment plant investigation to understand influent mass flux of per- and polyfluoroalkyl substances (PFAS). J. Hazard. Mater. 447, 130854. https://doi.org/10.1016/j.jhazmat.2023.130854.

Tang, S., He, C., Thai, P.K., Heffernan, A., Vijayasarathy, S., Toms, L., et al., 2020. Urinary concentrations of bisphenols in the Australian population and their association with the per capita mass loads in wastewater. Environ. Sci. Technol. 54, 10141–10148. https://doi.org/10.1021/acs.est.0c00921.

Tanui, I.C., Kandie, F., Krauss, M., Piotrowska, A., Finckh, S., Kiprop, A., et al., 2025. Occurrence and potential risk of steroid hormones in selected surface water and wastewater treatment plants in western Kenya. Environ. Pollut. 367, 125623. https://doi.org/10.1016/j.envpol.2024.125623.

Tappert, L., Bunge, M., Hoehne, D., Dlugi, I., Fetters, K., Fischer, B., et al., 2024. Bisphenol A in surface

waters in Germany: Part I. Reassessment of sources and emissions pathways for FlowEQ modeling. Integr. Environ. Assess. Manag. 20, 211–225. https://doi.org/10.1002/ieam.4805.

Tata, T., Belabed, B.E., Bououdina, M., Bellucci, S., 2020. Occurrence and characterization of surface sediment microplastics and litter from North African coasts of the Mediterranean Sea: Preliminary research and first evidence. Sci. Total Environ. 713, 136664. https://doi.org/10.1016/j.scitotenv.2020.136664.

Teixeira, L.C.G.M., das Chaves, J.R., Mendonça, N., Sanson, A.L., Alves, M.C.P., Afonso, R.J.C.F., et al., 2021. Occurrence and removal of drugs and endocrine disruptors in the Bolonha Water Treatment Plant in Belém/PA (Brazil). Environ. Monit. Assess. 193, 246. https://doi.org/10.1007/s10661-021-09025-x.

Terzi, Y., Gedik, K., Eryaşar, A.R., Öztürk, R.Ç., Şahin, A., Yılmaz, F., 2022. Microplastic contamination and characteristics spatially vary in the southern Black Sea beach sediment and sea surface water. Mar. Pollut. Bull. 174, 113228. https://doi.org/10.1016/j.marpolbul.2021.113228.

Thalla, A.K., Vannarath, A.S., 2020. Occurrence and environmental risks of nonsteroidal anti-inflammatory drugs in urban wastewater in the southwest monsoon region of India. Environ. Monit. Assess. 192, 193. https://doi.org/10.1007/s10661-020-8161-1.

Trindade, L.d.S., Gloaguen, T.V., Benevides, T.d.S.F., Valentim, A.C.S., Bomfim, M.R., Gonzaga Santos, J.A., 2023. Microplastics in surface waters of tropical estuaries around a densely populated Brazilian bay. Environ. Pollut. 323, 121224. https://doi.org/10.1016/j.envpol.2023.121224.

Uddin, S., Behbehani, M., Habibi, N., Faizuddin, M., Al-Murad, M., Martinez-Guijarro, K., et al., 2022. Microplastics in Kuwait's wastewater streams. Sustainability 14.

Ugboka, U.G., Ihedioha, J.N., Ekere, N.R., Okechukwu, F.O., 2022. Human health risk assessment of

bisphenol A released from polycarbonate drinking water bottles and carbonated drinks exposed to sunlight in Nigeria. Int. J. Environ. Anal. Chem. 102, 2830–2840. https://doi.org/10.1080/03067319.2020.1759572.

Üstün-Odabaşı, S., Maryam, B., Özdemir, N., Büyükgüngör, H., 2020. Occurrence and seasonal variations of pharmaceuticals and personal care products in drinking water and wastewater treatment plants in Samsun, Turkey. Environ. Earth Sci. 79, 311. https://doi.org/10.1007/s12665-020-09047-7.

Üstün, G.E., Bozdaş, K., Can, T., 2022. Abundance and characteristics of microplastics in an urban wastewater treatment plant in Turkey. Environ. Pollut. 310, 119890. https://doi.org/10.1016/j.envpol.2022.119890.

Uurasjärvi, E., Hartikainen, S., Setälä, O., Lehtiniemi, M., Koistinen, A.J.W.E.R., 2020. Microplastic concentrations, size distribution, and polymer types in the surface waters of a northern European lake. Water Environ. Res. 92, 149–156.

Valentić, L., Kozel, P., Pipan, T., 2022. Microplastic pollution in vulnerable karst environments: Case study from the Slovenian classical karst region. Acta Carsologica 51, 79–92. https://doi.org/10.3986/ac.v51i1.10597.

Vane, C.H., Kim, A.W., Lopes dos Santos, R.A., Moss-Hayes, V., 2022. Contrasting sewage, emerging and persistent organic pollutants in sediment cores from the River Thames estuary, London, England, UK. Mar. Pollut. Bull. 175, 113340. https://doi.org/10.1016/j.marpolbul.2022.113340.

Vanukon, M.S., Dehm, J., Pickering, T., Yabakiva, M., Rico, C., Hewavitharane, C., 2025. First assessment of microplastic concentrations in oysters, water, and sediment in Laucala Bay, Fiji Islands. Int. J. Environ. Sci. Technol. 22, 1327–1342. https://doi.org/10.1007/s13762-024-

05739-w.

Vassalle, L., García-Galán, M.J., Aquino, S.F., Afonso, R.J.d.C.F., Ferrer, I., Passos, F., et al., 2020. Can high rate algal ponds be used as post-treatment of UASB reactors to remove micropollutants? Chemosphere 248, 125969. https://doi.org/10.1016/j.chemosphere.2020.125969.

Vaudreuil, M.-A., Vo Duy, S., Munoz, G., Sauvé, S., 2022. Pharmaceutical pollution of hospital effluents and municipal wastewaters of Eastern Canada. Sci. Total Environ. 846, 157353. https://doi.org/10.1016/j.scitotenv.2022.157353.

Verlicchi, P., Grillini, V., 2020. Surface Water and Groundwater Quality in South Africa and Mozambique—Analysis of the Most Critical Pollutants for Drinking Purposes and Challenges in Water Treatment Selection. Water 12. https://doi.org/10.3390/w12010305.

Von Behren, J., Reynolds, P., Bradley, P.M., Gray, J.L., Kolpin, D.W., Romanok, K.M., et al., 2024. Per- and polyfluoroalkyl substances (PFAS) in drinking water in Southeast Los Angeles: Industrial legacy and environmental justice. Sci. Total Environ. 953, 176067. https://doi.org/10.1016/j.scitotenv.2024.176067.

Wang, C., Huang, P., Qiu, C., Li, J., Hu, S., Sun, L., et al., 2021a. Occurrence, migration and health risk of phthalates in tap water, barreled water and bottled water in Tianjin, China. J. Hazard. Mater. 408, 124891. https://doi.org/10.1016/j.jhazmat.2020.124891.

Wang, C., Ye, D., Li, X., Jia, Y., Zhao, L., Liu, S., et al., 2021b. Occurrence of pharmaceuticals and personal care products in bottled water and assessment of the associated risks. Environ. Int. 155, 106651. https://doi.org/10.1016/j.envint.2021.106651.

Wang, G., Lu, J., Li, W., Ning, J., Zhou, L., Tong, Y., et al., 2021c. Seasonal variation and risk assessment of microplastics in surface water of the Manas River Basin, China. Ecotoxicol. Environ. Saf.

208, 111477. https://doi.org/10.1016/j.ecoenv.2020.111477.

Wang, H., Zhang, C., Zhang, X., Wang, S., Xia, Z., Zeng, G., et al., 2022a. Construction of Fe3O4@β-CD/g-C3N4 nanocomposite catalyst for degradation of PCBs in wastewater through photodegradation and heterogeneous Fenton oxidation. Chem. Eng. J. 429, 132445. https://doi.org/10.1016/j.cej.2021.132445.

Wang, K., Zhuang, T., Su, Z., Chi, M., Wang, H., 2021d. Antibiotic residues in wastewaters from sewage treatment plants and pharmaceutical industries: Occurrence, removal and environmental impacts. Sci. Total Environ. 788, 147811. https://doi.org/10.1016/j.scitotenv.2021.147811.

Wang, Y.-Q., Hu, L.-X., Liu, T., Zhao, J.-H., Yang, Y.-Y., Liu, Y.-S., et al., 2022b. Per- and polyfluoroalkyl substances (PFAS) in drinking water system: Target and non-target screening and removal assessment. Environ. Int. 163, 107219. https://doi.org/10.1016/j.envint.2022.107219.

Wang, Y., Cun, D., Zhang, Z., Pu, D., Li, X., Liang, W., et al., 2022c. Occurrence and risk assessment of triclosan in freshwater lakes in the middle Yangtze River basin (Wuhan, Central China). Water Biology and Security 1, 100063. https://doi.org/10.1016/j.watbs.2022.100063.

Watkinson, A.J., Murby, E.J., Costanzo, S.D., 2007. Removal of antibiotics in conventional and advanced wastewater treatment: Implications for environmental discharge and wastewater recycling. Water Res. 41, 4164–4176. https://doi.org/10.1016/j.watres.2007.04.005.

Wei, Z., Wei, T., Chen, Y., Zhou, R., Zhang, L., Zhong, S., 2024. Seasonal dynamics and typology of microplastic pollution in Huixian karst wetland groundwater: Implications for ecosystem health. J. Environ. Manag. 358, 120882. https://doi.org/10.1016/j.jenvman.2024.120882.

Wicaksono, E.A., Werorilangi, S., Galloway, T.S., Tahir, A., 2021. Distribution and Seasonal Variation

of Microplastics in Tallo River, Makassar, Eastern Indonesia. Toxics 9. https://doi.org/10.3390/toxics9060129.

Wu, B., Li, L.-W., Zu, Y.-X., Nan, J., Chen, X.-Q., Sun, K., et al., 2022. Microplastics contamination in groundwater of a drinking-water source area, northern China. Environ. Res. 214, 114048. https://doi.org/10.1016/j.envres.2022.114048.

Wu, P.-H., Yeh, H.-Y., Chou, P.-H., Hsiao, W.-W., Yu, C.-P., 2021. Algal extracellular organic matter mediated photocatalytic degradation of estrogens. Ecotoxicol. Environ. Saf. 209, 111818. https://doi.org/10.1016/j.ecoenv.2020.111818.

Wu, P., Tang, Y., Dang, M., Wang, S., Jin, H., Liu, Y., et al., 2020. Spatial-temporal distribution of microplastics in surface water and sediments of Maozhou River within Guangdong-Hong Kong-Macao Greater Bay Area. Sci. Total Environ. 717, 135187. https://doi.org/10.1016/j.scitotenv.2019.135187.

Xiong, X., Tappenbeck, T.H., Wu, C., Elser, J.J., 2022. Microplastics in Flathead Lake, a large oligotrophic mountain lake in the USA. Environ. Pollut. 306, 119445. https://doi.org/10.1016/j.envpol.2022.119445.

Xu, N., Jiang, L., Zhang, Y., Shen, Y., Wang, Y., Wang, S., et al., 2023. Microplastic pollution in the offshore sea, rivers and wastewater treatment plants in Jiangsu coastal area in China. Mar. Environ. Res. 188, 105992. https://doi.org/10.1016/j.marenvres.2023.105992.

Xu, X., Xu, Y., Xu, N., Pan, B., Ni, J., 2022. Pharmaceuticals and personal care products (PPCPs) in water, sediment and freshwater mollusks of the Dongting Lake downstream the Three Gorges Dam. Chemosphere 301, 134721. https://doi.org/10.1016/j.chemosphere.2022.134721.

Yadav, H., Sethulekshmi, S., Shriwastav, A., 2022. Estimation of microplastic exposure via the composite

sampling of drinking water, respirable air, and cooked food from Mumbai, India. Environ. Res. 214, 113735. https://doi.org/10.1016/j.envres.2022.113735.

Yang, L., Wang, T., Zhou, Y., Shi, B., Bi, R., Meng, J., 2021. Contamination, source and potential risks of pharmaceuticals and personal products (PPCPs) in Baiyangdian Basin, an intensive human intervention area, China. Sci. Total Environ. 760, 144080. https://doi.org/10.1016/j.scitotenv.2020.144080.

Yang, L., Zhou, Y., Shi, B., Meng, J., He, B., Yang, H., et al., 2020a. Anthropogenic impacts on the contamination of pharmaceuticals and personal care products (PPCPs) in the coastal environments of the Yellow and Bohai seas. Environ. Int. 135, 105306. https://doi.org/10.1016/j.envint.2019.105306.

Yang, Y., Hu, M., Lu, W., Xue, L., Lin, X., Liu, E., 2020b. Occurrence, distribution, and risk assessment of PPCPs in water and sediments of Longgang River in Shenzhen City, south China. Desalination and Water Treatment 189, 196–206. https://doi.org/10.5004/dwt.2020.25608.

Yang, Y., Ji, Y., Gao, Y., Lin, Z., Lin, Y., Lu, Y., et al., 2022. Antibiotics and antimycotics in wastewater treatment plants: Concentrations, removal efficiency, spatial and temporal variations, prediction, and ecological risk assessment. Environ. Res. 215, 114135. https://doi.org/10.1016/j.envres.2022.114135.

Yi, J., Huang, X., Hou, J., Xiong, J., Qian, Z., Liu, S., et al., 2023. Occurrence and distribution of PPCPs in water from two largest urban lakes of China: First perspective from DGT in-situ measurement. Sci. Total Environ. 904, 166656. https://doi.org/10.1016/j.scitotenv.2023.166656.

Yu, X., Wang, Y., Watson, P., Yang, X., Liu, H., 2024. Application of passive sampling device for exploring the occurrence, distribution, and risk of pharmaceuticals and pesticides in surface

water. Sci. Total Environ. 908, 168393. https://doi.org/10.1016/j.scitotenv.2023.168393.

Yu, X., Yu, F., Li, Z., Zhan, J., 2023. Occurrence, distribution, and ecological risk assessment of pharmaceuticals and personal care products in the surface water of the middle and lower reaches of the Yellow River (Henan section). J. Hazard. Mater. 443, 130369. https://doi.org/10.1016/j.jhazmat.2022.130369.

Yuan, D., Corvianawatie, C., Cordova, M.R., Surinati, D., Li, Y., Wang, Z., et al., 2023. Microplastics in the tropical Northwestern Pacific Ocean and the Indonesian seas. J. Sea Res. 194, 102406. https://doi.org/10.1016/j.seares.2023.102406.

Yuan, X., Hu, J., Li, S., Yu, M., 2020. Occurrence, fate, and mass balance of selected pharmaceutical and personal care products (PPCPs) in an urbanized river. Environ. Pollut. 266, 115340. https://doi.org/10.1016/j.envpol.2020.115340.

Yüksel, S., Kabay, N., Yüksel, M., 2013. Removal of bisphenol A (BPA) from water by various nanofiltration (NF) and reverse osmosis (RO) membranes. J. Hazard. Mater. 263, 307–310. https://doi.org/10.1016/j.jhazmat.2013.05.020.

Zafar, R., Bashir, S., Nabi, D., Arshad, M., 2021. Occurrence and quantification of prevalent antibiotics in wastewater samples from Rawalpindi and Islamabad, Pakistan. Sci. Total Environ. 764, 142596. https://doi.org/10.1016/j.scitotenv.2020.142596.

Zainab, S.M., Junaid, M., Rehman, M.Y.A., Lv, M., Yue, L., Xu, N., et al., 2021. First insight into the occurrence, spatial distribution, sources, and risks assessment of antibiotics in groundwater from major urban-rural settings of Pakistan. Sci. Total Environ. 791, 148298. https://doi.org/10.1016/j.scitotenv.2021.148298.

Zeri, C., Adamopoulou, A., Koi, A., Koutsikos, N., Lytras, E., Dimitriou, E., 2021. Rivers and

Wastewater-Treatment Plants as Microplastic Pathways to Eastern Mediterranean Waters: First Records for the Aegean Sea, Greece. Sustainability 13. https://doi.org/10.3390/su13105328.

Zhai, Y., Dai, Y., Guo, J., Zhou, L., Chen, M., Yang, H., et al., 2020. Novel biochar@CoFe2O4/Ag3PO4 photocatalysts for highly efficient degradation of bisphenol A under visible-light irradiation. J. Colloid Interface Sci. 560, 111–121. https://doi.org/10.1016/j.jcis.2019.08.065.

Zhan, S., Huang, H., He, C., Xiong, Y., Li, P., Tian, S., 2023. Controllable synthesis of substitutional and interstitial nitrogen-doped ceria: The effects of doping sites on enhanced catalytic ozonation of organic pollutants. Appl. Catal. B Environ. 321, 122040. https://doi.org/10.1016/j.apcatb.2022.122040.

Zhang, L., Liu, J., Xie, Y., Zhong, S., Yang, B., Lu, D., et al., 2020a. Distribution of microplastics in surface water and sediments of Qin river in Beibu Gulf, China. Sci. Total Environ. 708, 135176. https://doi.org/10.1016/j.scitotenv.2019.135176.

Zhang, P., Liu, S., Tan, X., Liu, Y., Zeng, G., Yin, Z., et al., 2019. Microwave-assisted chemical modification method for surface regulation of biochar and its application for estrogen removal. Process Saf. Environ. Prot. 128, 329–341. https://doi.org/10.1016/j.psep.2019.06.009.

Zhang, W., Zhang, S., Zhao, Q., Qu, L., Ma, D., Wang, J., 2020b. Spatio-temporal distribution of plastic and microplastic debris in the surface water of the Bohai Sea, China. Mar. Pollut. Bull. 158, 111343. https://doi.org/10.1016/j.marpolbul.2020.111343.

Zhang, Y., Dong, R., Ge, F., Hong, M., Chen, Z., Zhou, Y., et al., 2024. Removal of 48 per- and polyfluoroalkyl substances (PFAS) throughout processes in domestic and general industrial wastewater treatment plants: Implications for emerging alternatives risk control. J. Hazard. Mater. 480, 136130. https://doi.org/10.1016/j.jhazmat.2024.136130.

Zheng, G., Yu, B., Wang, Y., Ma, C., Chen, T., 2020. Removal of triclosan during wastewater treatment process and sewage sludge composting—A case study in the middle reaches of the Yellow River. Environ. Int. 134, 105300. https://doi.org/10.1016/j.envint.2019.105300.

Zheng, X., Zhou, C., Wu, F., Xu, H., Zhao, Z., Han, Z., et al., 2023. Enhanced removal of organic, nutrients, and PFCs in the iron-carbon micro-electrolysis constructed wetlands: Mechanism and iron cycle. Chem. Eng. J. 457, 141174. https://doi.org/10.1016/j.cej.2022.141174.

Zhong, S.-F., Yang, B., Lei, H.-J., Xiong, Q., Zhang, Q.-Q., Liu, F., et al., 2022. Transformation products of tetracyclines in three typical municipal wastewater treatment plants. Sci. Total Environ. 830, 154647. https://doi.org/10.1016/j.scitotenv.2022.154647.

Zhou, P., Li, Z., El-Dakhakhni, W., Smyth, S.A., 2022. Prediction of bisphenol A contamination in Canadian municipal wastewater. J. Water Process Eng. 50, 103304. https://doi.org/10.1016/j.jwpe.2022.103304.

Zhou, X.-j., Wang, J., Li, H.-y., Zhang, H.-m., Hua, J., Zhang, D.L., 2021. Microplastic pollution of bottled water in China. J. Water Process Eng. 40, 101884. https://doi.org/10.1016/j.jwpe.2020.101884.

Zhu, N., Li, C., Bu, L., Tang, C., Wang, S., Duan, P., et al., 2020. Bismuth impregnated biochar for efficient estrone degradation: The synergistic effect between biochar and Bi/Bi2O3 for a high photocatalytic performance. J. Hazard. Mater. 384, 121258. https://doi.org/10.1016/j.jhazmat.2019.121258.

Ziajahromi, S., Neale, P.A., Telles Silveira, I., Chua, A., Leusch, F.D.L., 2021. An audit of microplastic abundance throughout three Australian wastewater treatment plants. Chemosphere 263, 128294. https://doi.org/10.1016/j.chemosphere.2020.128294.

Zwart, N., Jonker, W., Broek, R.t., de Boer, J., Somsen, G., Kool, J., et al., 2020. Identification of mutagenic and endocrine disrupting compounds in surface water and wastewater treatment plant effluents using high-resolution effect-directed analysis. Water Res. 168, 115204. https://doi.org/10.1016/j.watres.2019.115204.
